# A (poly)Pro tip for preserving native disulfide connectivity during thiol–maleimide bioconjugation of disulfide-rich peptides

**DOI:** 10.1039/d5sc08821f

**Published:** 2026-03-30

**Authors:** Lylia Azzoug, Ana Novak, Hervé Meudal, Jean-Baptiste Madinier, Stéphane Charpentier, Karine Loth, Séverine Morisset-Lopez, Carlo Pifferi, Vincent Aucagne

**Affiliations:** a Centre de Biophysique Moléculaire, CNRS UPR 4301 Rue Charles Sadron 45071 Orléans France carlo.pifferi@cnrs-orleans.fr vincent.aucagne@cnrs-orleans.fr; b UFR Sciences et Techniques, Université d'Orléans Rue de Chartres 45067 Orléans France

## Abstract

Generation of specific antibodies against peptides by immunization requires their covalent conjugation to protein carriers to override their inherently weak immunogenicity. The vast majority of bioconjugation approaches to achieve peptide–protein constructs rely on thiol–maleimide chemistry and capitalize on a wide array of commercial maleimide-functionalized protein carriers. Disulfide-rich peptides (DRPs) possess a rigid, constrained structure that makes them ideal for designing synthetic mimics of protein regions/domains. For bioconjugation purposes, the introduction of a single spare thiol moiety into a linear peptide antigen is straightforward, while DRPs' disulfide bonds are prone to intramolecular thiophilic attack by the reactive thiolate. This unintended reactivity competes with the desired Michael addition to the maleimide moiety, ultimately disrupting the native disulfide bridging framework. As a result, DRP's tertiary structure will be altered, affording an immunogen that is a poor mimic of the native target. Although a few studies have explored the late-stage introduction of thiol-containing cross-linkers into DRP antigens for their conjugation onto protein carriers, the stability of DRPs' disulfide pattern in the presence of an extra thiol has never been examined. In this study, we systematically evaluated the influence of different spacers in “DRP–spacer–thiol” constructs under thiol–maleimide reaction conditions. Our results highlight how both linker length and flexibility are key to maintaining DRP disulfides unaltered, providing a general approach to achieve DRP bioconjugation by thiol–maleimide chemistry. We have applied our approach to a small DRP predicted to closely mimic a surface-accessible epitope of the full LINGO-1 protein and obtained a very specific antibody response upon immunization; the resulting polyclonal IgG was able to selectively bind the full-length protein in a cellular context, with stringent selectivity across its four homologs.

## Abstract 2

Thiol–maleimide chemistry is the most common strategy to link peptide antigens to carrier proteins. In disulfide-rich peptides, an extra thiol may disrupt native S–S bridges. We show that introducing an optimized spacer can bypass this limitation.

## Graphical abstract

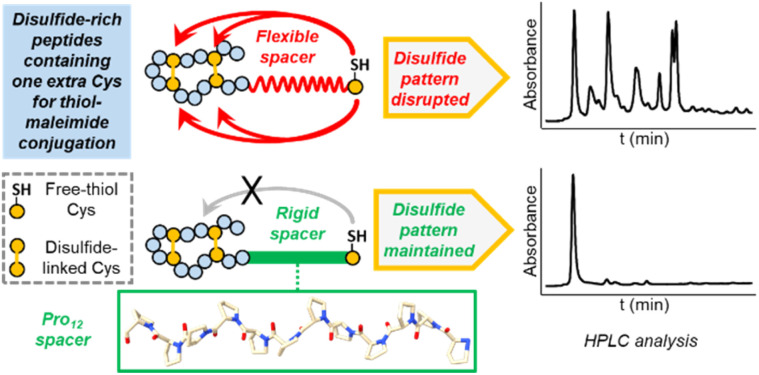

## Introduction

Disulfide-rich peptides^1^ (DRPs) are a widespread class of natural compounds typically containing up to a few dozen amino acid (AA) residues, 10 to 30% of which are cysteines crosslinked through S–S bonds. Contrarily to linear, non-crosslinked peptides of similar sizes, DRPs have highly constrained conformations endowing them with a much superior stability, both upon degradation by proteolytic enzymes and upon denaturation by heat and chaotropic agents. In addition, such conformational bias often endows them with an enhanced ability to interact specifically with their biological targets, due to pre-organization and the reduced entropic penalty upon binding. Naturally-occurring DRPs include, for example, defensins,^2^ major players of innate immunity, and animal venom toxins that act as highly-potent ligands of a wide range of pharmaceutically-relevant receptors.^3,4^ Both continue to attract significant attention as sources for drug development. Considering their favorable pharmaceutical properties, DRP architectures are also largely exploited as scaffolds for the conception of affinity ligands. In such instances, their backbone loops, delimited by cysteine residues, can serve as grafting sites for an exogenous bioactive paratope,^5^ or for randomized peptide sequences that give rise to *de novo* paratopes through combinatorial screening assays.^6–8^ In addition to single-chain DRPs, the family also encompasses insulin- and relaxin-related peptides, which are heterodimers constituted by two peptide chains crosslinked through inter- and intra-chain disulfide bonds. In addition to existing as isolated molecules, DRP segments are also frequently present as parts of larger proteins. They can either form independent protein domains,^9^ such as epidermal growth factor-like^10^ and cystine-knot^11^ domains, or be embedded within larger domains, where they act as fold-stabilizing units—for example, the C-ter and N-ter disulfide-rich “caps” flanking leucine-rich repeat (LRR) domains.^12,13^ Such DRP segments therefore represent valuable molecular motifs to target for imaging or for modulating the activity of their parent proteins.

Given the significance of DRPs in biomedicine and fundamental biology, a wide range of commercially available antibodies—mainly targeting defensins—have been developed to provide biochemical and histochemical tools for studying their modes of action and validating their potential as biomedical agents. In general, antibodies designed to specifically bind peptide or protein targets are laboratory workhorses in contemporary biology, available either in a polyclonal form (consisting of a heterogeneous mixture of antibodies raised in an animal against a given immunogen, which represents the majority of commercially available bioreagents) or in a monoclonal form (a single antibody species). However, the validation of the target specificity and their ability to bind either denatured or folded forms has emerged in the past decade as a major concern.^14,15^

**Scheme 1:**
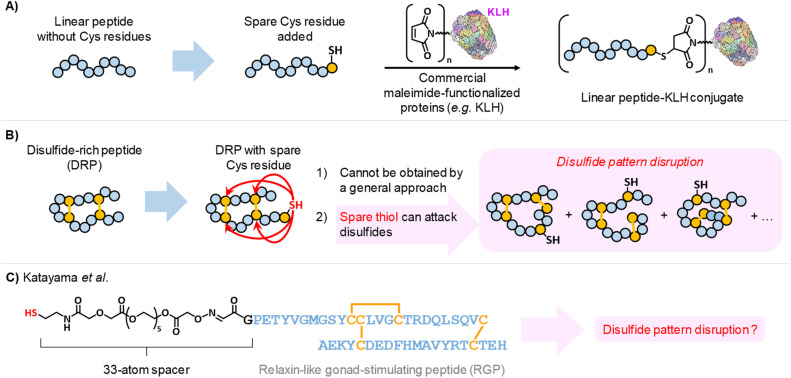
(A) Adding a single Cys residue to a linear peptide enables straightforward, site-selective bioconjugation by thiol–maleimide chemistry. (B) In contrast, introducing an additional Cys residue into a disulfide-rich peptide (DRP) is not more challenging, as the free thiol can attack disulfide bonds and disrupt the disulfide pattern. (C) Katayama *et al.*^33^ equipped a DRP with a 33-atom spacer to reduce the likelihood of intramolecular thiol attack on disulfide bonds, although disulfide pattern disruption was not assessed prior to conjugation to KLH.

Immunization-based approaches for the generation of DRP-specific antibodies can leverage the use of large DRPs, typically ≥100 amino acid (AA) residues, as immunogens.^14,16^ Nevertheless, smaller DRPs often must be covalently linked to larger proteins to compensate for their low immunogenicity.^17^ Indeed, proteins are much more effective than peptides (*e.g.* ≈10–30 AAs) for antibody elicitation because they offer greater structural complexity and epitope diversity, allowing the immune system to recognize multiple antigenic sites. Uptake and processing by antigen-presenting cells are more efficient for larger immunogens, leading to enhanced immune activation. Moreover, larger sequences are more likely to contain both B-cell and T-cell epitopes, ensuring strong helper T-cell activation, which is crucial for antibody class switching and affinity maturation.^18^ Classical approaches for boosting the immunogenicity of short peptides include: (i) increasing the overall size of the immunogen,^19^ (ii) displaying the target epitope in a multivalent fashion,^20^ and (iii) including known CD4^+^ epitopes^21^ to ensure helper T-cell activation. The most widely adopted strategy, which combines all three features, relies on the preparation of an immunogen construct consisting in multiple copies of the peptide antigen covalently linked to a high-molecular-weight carrier protein bearing multiple CD4^+^ epitopes, such as keyhole limpet hemocyanin (KLH), bovine serum albumin (BSA), or ovalbumin (OVA). The method of choice for preparing such peptide–carrier protein conjugates is the thiol–maleimide Michael addition reaction,^22,23^ and many companies now offer on-demand immunization services based on this chemistry. This involves adding a spare thiol onto the target peptide antigen – most often from an additional Cys residue – which then reacts with maleimide groups on the protein carrier (Scheme 1A). In addition to its exquisite chemo-selectivity and fast kinetics, typically requiring only a few hours at sub-millimolar concentration and near-neutral pH, another advantage of thiol–maleimide coupling is the commercial availability of a wide range of maleimide-functionalized carrier proteins.

However, while introducing a spare thiol into a peptide that lacks cysteine residues is straightforward, it becomes far more challenging when the peptide already contains cysteines engaged in disulfide bonds (Scheme 1B). The two primary challenges are (i) designing a protecting group strategy that ensures correct disulfide bond formation while still yielding the desired free thiol, and (ii) preventing the DRP disulfide bonds from being attacked by the spare thiol in its thiolate form, which can cause disulfide shuffling. This extremely fast thiolate/disulfide equilibrium reaction has been largely exploited for generating dynamic combinatorial libraries^24^ or thermodynamically-controlled oxidative folding of DRPs,^25^ but is highly unwanted in the case of DRP immunogen preparation. Indeed, for DRPs equipped with a spare thiol, intramolecular thiophilic attack can compete with the desired Michael addition to the maleimide moiety on the protein carrier, resulting in a heterogeneous mixture of regioisomers (Scheme 1B). Such disruption of the disulfide bond pattern during bioconjugation is highly detrimental to the generation of DRP-specific antibodies and must be avoided, particularly when antibodies that recognize the native, disulfide-constrained form of the DRP are required. This is particularly critical for techniques that rely on native protein structures, such as immunohistochemistry, co-immunoprecipitation, or cell-based ELISA, but less so for western blotting, where proteins are typically reduced and denatured before SDS-PAGE.

As an alternative to thiol–maleimide conjugation, DRP-based immunogens have been generated *via* the expression of a chimeric protein in *E. coli* (*e.g.* fusion of a defensin with a glutathione *S*-transferase, GST^26^). However, there are limitations that may compromise the structural homogeneity of the resulting construct. These include difficulties in achieving controlled disulfide bond formation and proper folding of the DRP component during fusion protein expression, as well as the lack of verification of its correct three-dimensional structure. Another approach is DRP conjugation to carrier proteins using non-selective crosslinking agents that target surface exposed amino or carboxyl groups.^27^ Although this strategy can yield larger immunogens displaying multiple DRP copies, conjugation at random sites may mask critical epitopes and impair immune recognition. Additionally, random attachment can alter the native conformation and reduce the likelihood of generating antibodies that recognize the DRP in its native context. Although alternative strategies not based on thiol–maleimide chemistry—such as the use of non-canonical amino acids for chemoselective ligation or enzymatic conjugation approaches—have demonstrated their efficacy for DRP conjugation and could, in principle, be employed,^28–30^ thiol–maleimide coupling remains the method of choice for preparing peptide–carrier protein immunogens in both academic and commercial settings, largely due to the commercial availability of maleimide-derivatized carrier proteins and the lack of readily available carrier proteins functionalized with other chemoselectively reacting groups.††Thiol–maleimide conjugates can undergo retro-Michael reactions in physiological environments, with these processes typically occurring on characteristic timescales of tens of hours in model systems.^23^ A number of elegant strategies have been proposed to prevent such de-conjugation^23,68–70^ for applications in which a long-lasting stability of the injected conjugate is crucial, for example, antibody conjugated with highly cytotoxic drugs (ADC). In contrast, protein-based immunogens are accessed by the immune system on very short timescales: antigen can reach draining lymph nodes within minutes after injection, cognate B cells can acquire antigen within minutes, and lymph node processing is detectable within approximately 60–90 minutes,^71,72^ indicating that critical early sampling occurs on a minutes-to-hours timescale. This is probably why thiol–maleimide chemistry remains the reference site-selective approach for the generation of immunogens, employed in countless vaccination studies over several decades, and is the standard approach in commercial immunization services; in addition, a broad range of carrier proteins pre-functionalized with maleimides is commercially available at affordable costs. This choice is further supported by the intrinsic advantages of maleimide reagents, including their rapid reaction kinetics with thiols, high chemoselectivity, and consistently high conjugation efficiencies under mild conditions.

To take advantage of thiol–maleimide chemistry while avoiding thiolate–disulfide exchange, short DRP segments lacking disulfide bonds have been used for conjugation to carrier proteins.^31^ Many commercially available anti-defensin antibodies have been raised using such immunogens. While such antibodies may recognize linearized versions of the DRP (*e.g.* in western blots, under reducing conditions), their ability to bind to the conformationally-constrained native form remains uncertain, and some studies have reported a lack of binding. For example, antibodies raised against peptides from either A or B chains of a heterodimeric DRP relaxin-like gonad-stimulating peptide (RGP) were unable to recognize the native RGP.^32^ These results emphasize the importance of designing immunogens that present the full DRP in its native three-dimensional structure, defined by its native disulfide bond connectivity.

A major advance in the chemoselective preparation of DRP–carrier protein conjugates for raising DRP-specific polyclonal antibodies was reported in 2016 by Katayama and co-workers.^32^ They devised an elegant strategy in which a spare thiol moiety was introduced into chemically synthesized *Patiria pectinifera* starfish RGP through late-stage oxime ligation (Scheme 1C):^32,33^ a chemoselective reaction proceeding under acidic conditions and thus preventing thiolate generation and disulfide shuffling. The spare thiol was separated from the DRP core by a long and flexible 33-atom oligo(ethylene glycol) linker. Thiol–maleimide chemistry was then used to prepare the corresponding KLH-based immunogen, with the spacer designed to minimize intramolecular attack of RGP disulfides by reducing the effective molarity of the reactive thiolate. The strategy yielded specific antibodies against native RGP. However, preservation of the original disulfide bond pattern in the “DRP–spacer–thiol” constructs was not assessed, likely because the KLH conjugation and immunization were outsourced to a private company.

**Scheme 2:**
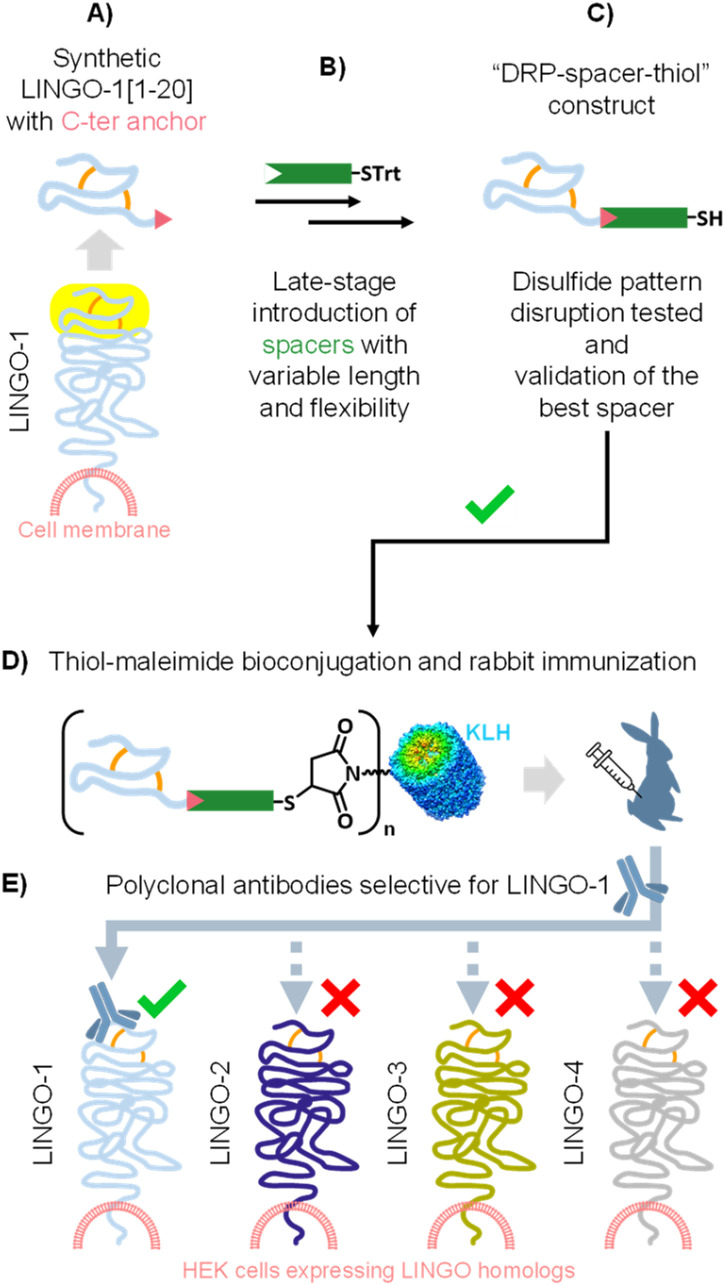
Overall workflow for this study.

Given our long-standing interest in the chemical synthesis, structural characterization, conjugation, and biological evaluation of various DRPs,^14,34–39^ we were intrigued by the broad applicability of Katayama's approach for generating DRP-protein conjugates as immunogens for *in vivo* antibody production. We were therefore particularly interested in verifying that the disulfide pattern of our DRP was maintained unaltered under thiol–maleimide bioconjugation conditions. In this report, we have systematically monitored the occurrence of disulfide pattern disruption of “DRP–spacer–thiol” constructs equipped with variable linkers in terms of rigidity and length. To do this, we have selected a small DRP displaying four Cys residues located at the extracellular N-terminus of the LINGO-1 transmembrane signaling protein (Scheme 2A).^40^

First, we have implemented a robust strategy for the late-stage introduction of thiol-containing spacers of varying length and flexibility onto this small DRP (Scheme 2B). Then, we systematically evaluated a series of “DRP–spacer–thiol” constructs by incubating them in thiol–maleimide reaction buffer, and found that flexible spacers, even up to 141-atom in length, could not fully prevent disulfide pattern disruption in LINGO-1[1–20]. In sharp contrast, a rigid, proline-based 54-atom spacer allowed maintenance of ≈100% of the LINGO-1[1–20] native disulfide pattern (Scheme 2C). We then conjugated our best-performing “DRP–spacer–thiol” construct to KLH and successfully generated specific anti- LINGO-1[1–20] antibodies by rabbit immunization (Scheme 2D). Importantly, the resulting rabbit polyclonal IgG effectively recognized LINGO-1 in a cellular context and showed specificity across its homologs (Scheme 2E).

## Results and discussion

### NMR studies on the LINGO-1[1–20] N-cap region

**Fig. 1:**
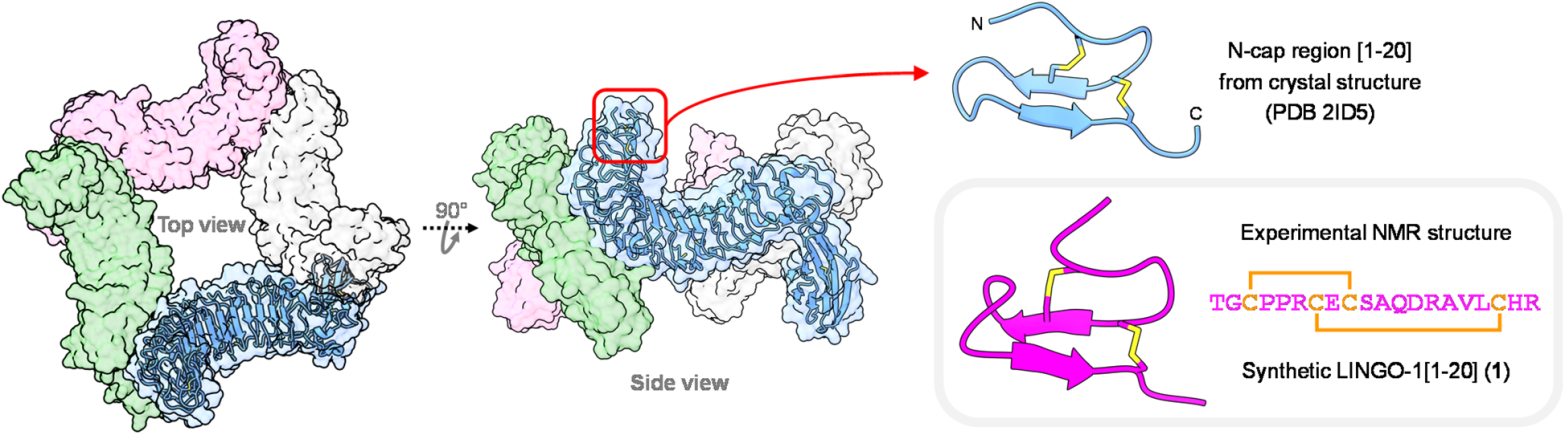
Crystal structure of tetrameric ectodomain of LINGO-1 (left). Structure of the LINGO-1 N-cap [1–20] region, displaying a DRP motif (top right, cornflower blue). 3D NMR solution structure obtained for synthetic LINGO-1[1–20], 1 (box, bottom right, magenta).

LINGO-1 (Leucine-rich repeat and Ig domain-containing Nogo receptor-interacting protein) is a transmembrane cell-surface glycoprotein of 579 amino acids (AAs) with a large extracellular portion (520 AAs ectodomain) displaying 12 leucine-rich repeat (LRR) motifs flanked with N- and C-terminal disulfide-rich capping segments (N-cap and C-cap) and an immunoglobulin domain.^41^ LINGO-1 is highly expressed in the brain and spinal cord, in both oligodendrocytes and neurons, and its inhibitory function with respect to axonal myelination and functional recovery has made it an important pharmacological target to address myelination disorders such as multiple sclerosis and spinal cord injury.^42–45^ From a structural biology perspective, LINGO-1 ectodomain adopts a stable, ring-shaped tetrameric organization (Fig. 1),^40^ observed both in crystals and in solution,^46^ where the Ig domain of one unit makes contacts with the LRR domain of the adjacent one.

In this study, we have focused our attention on the N-cap of the LINGO-1 LRR domain, which displays a 20-mer DRP sequence, LINGO-1[1–20], containing four Cys residues that are engaged in disulfide bonds (Cys3–Cys9 and Cys7–Cys18), and is devoid of other post-translational modifications (Fig. 1). The expected accessibility of this small DRP segment in a cellular context can likely be exploited for detection purposes. LRR domain N- and C-caps contribute to the stability and structural integrity of adjacent repeats and of the whole domain.^47,48^ This notion led us to hypothesize that an isolated cap-derived segment such as LINGO1[1–20] could be, in turn, also less stable when the downstream LRR context is absent, and thus be particularly challenging, constituting an appropriate difficult model DRP to test our methodology.

The N-cap of all four LINGO homologs displays the same Cys pattern and exhibits sequence identities ranging from 45% to 75%. This adds an additional challenge for our strategy: eliciting a highly specific IgG response against LINGO-1[1–20] could enable selective targeting of LINGO-1 over its homologs, thereby reinforcing the strength of our approach.

Synthetic LINGO-1[1–20] (1, Fig. 1) was prepared by standard Fmoc-based solid-phase peptide synthesis (SPPS) using *S*-Trt/*S*-Acm orthogonally protected cysteine pairs, followed by a directed disulfide bond formation protocol (Scheme S1).^49^ We chose this protection-group-directed strategy rather than classical thermodynamically controlled oxidative folding to ensure the formation of the native cysteine connectivity. In thermodynamically controlled oxidative folding, disulfide formation and reshuffling are promoted in a redox buffer (typically glutathione/glutathione disulfide) under mildly basic conditions, allowing the most stable disulfide connectivity isomer—often corresponding to the native structure—to predominate. However, for LINGO-1[1–20], which is not an isolated domain but only a portion of it, we anticipated that such an approach could be biased due to the absence of the adjacent leucine-rich repeats. This hypothesis was later confirmed: an isolated peptide containing the native disulfide bonds was unstable under oxidative conditions and underwent disulfide reshuffling, leading to alternative connectivity isomers. A control experiment with a single-domain DRP, king penguin β-defensin AvBD103b,^39^ containing three disulfide bridges, showed no significant changes in the HPLC chromatogram under similar conditions (see SI, p. S31).‡‡The evaluation of disulfide pattern stability under oxidative conditions was performed on azido derivative 4, not on LINGO-1[1–20] 1.

Initial structure calculations were performed using so-called ambiguous disulfide restraints, in which cysteine residues were defined in their deprotonated state and sulfur–sulfur distances were restrained to be compatible with disulfide bond formation, while allowing all possible cysteine pairing combinations. Under these conditions, the number of experimental restraints was sufficient to define a well-converged global fold of the peptide, but did not lead to systematic convergence of the structure calculation toward a unique three-dimensional structure with a fully defined disulfide connectivity.

Nevertheless, the overall fold was preserved across the calculated ensemble, and in a subset of structures, the Cys3–Cys9 disulfide bond formed spontaneously. Importantly, in all structures, the folding consistently positioned the cysteine side chains in spatial arrangements compatible with the formation of both Cys3–Cys9 and Cys7–Cys18 disulfide bridges, indicating that the native disulfide pattern would be stabilized in the calculated structure upon increasing the number of structural restraints (see Fig. S9).

Based on these observations, subsequent structure calculations were performed by explicitly imposing covalent restraints between the relevant cysteine side chains to reflect the experimentally defined disulfide connectivity. This strategy is consistent with the synthetic protocol, which was designed to selectively enforce the native disulfide pattern, and with the analytical characterization of the final compound, which confirmed the presence of a single, homogeneous species and not a mixture of different isomers differing in their disulfide connectivity.

Fig. 1 shows a representative solution NMR structure of 1 (see SI, pp. S12–S19), revealing the characteristic antiparallel β-sheet architecture observed for the LINGO-1[1–20] region in X-ray crystallography structures of the full-length ectodomain.^40^ A flexible turn connects the two β-strands, whereas a more rigid turn containing two consecutive Pro residues is located between Cys3 and Cys7. Overall, structural comparison with the corresponding N-terminal region of native LINGO-1 shows that the isolated DRP accurately mimics the protein fold, with a backbone RMSD of 1.93 ± 0.78 Å over residues 1–20.

After validating that the LINGO-1[1–20] DRP is properly structured to mimic the protein's N-terminal region, making it a suitable candidate for raising antibodies that specifically target this region, we next designed DRP–spacer–thiol constructs incorporating spacers of varying length and flexibility. These were then evaluated for their ability to prevent disulfide pattern disruption under thiol-Michael reaction conditions.

### Design, synthesis and stability evaluation of “DRP–spacer–thiol” systems

**Scheme 3:**
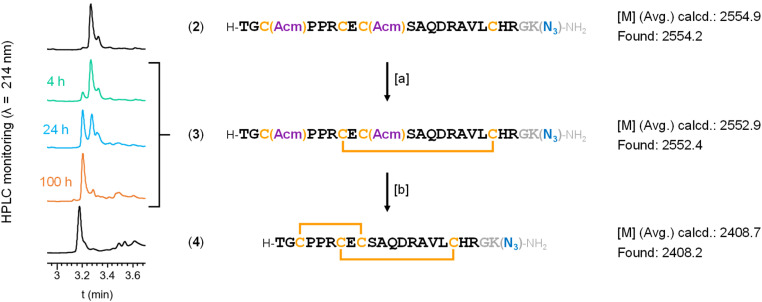
Synthesis of C-terminal amidated LINGO-1[1–20]-Gly-Anl 4. Reagents and conditions: [a] DMSO/H_2_O – 1 : 9, peptide conc. 0.32 mM*, 100 h, r.t.; [b] AcOH/160 mM aq. HCl – 93.6 : 6.4 containing 62.5 mM of I_2_, peptide conc. 1 mM*, 2 h, r.t. Abbreviations: Anl = 6-Azido-l-norleucine, represented in the scheme as “K(N_3_)”; DMSO = dimethyl sulfoxide; r.t. = room temperature. *Steps [a] and [b] were carried out on crude material, with concentrations calculated from the crude mass as if it corresponded to the pure compound.

For the synthesis of our DRP–spacer–thiol constructs, we avoided N-terminal conjugation, as in the synthetic strategy used by Katayama,^32,33^ because this region is likely the most accessible part of the LINGO-1 transmembrane protein for antibody recognition. The two key synthons consisted of (i) the [1–20] DRP in its “oxidized”, two-disulfide-crosslinked form, equipped with a suitable chemical handle at its C-terminal end, and (ii) a set of spacers with the complementary chemical handle on one end and a trityl (Trt)-protected thiol on the other. The Trt group fully prevents thiol–disulfide exchange and can be removed by mild treatment without additional purification. Among possible chemoselective ligation reactions options to covalently link DRP and spacer–thiol modules, we selected the Cu(i)-catalyzed azide–alkyne cycloaddition (CuAAC), because azide/terminal-alkyne handles are readily introduced by SPPS from commercially available building blocks, and because these functional groups are conveniently unreactive in the absence of a copper catalyst. Thus, we incorporated an azidonorleucine (Anl) residue at the C-terminus, separated from the DRP sequence by an additional Gly residue (Scheme 3). Linear, partially-protected sequence 2 (Scheme 3) was synthesized by automated Fmoc-SPPS, and the resulting crude solubilized in a 10% dimethyl sulfoxide (DMSO) solution in water to induce disulfide formation between Cys7 and Cys18.^50^ Conversion into intermediate 3 was monitored by HPLC and LC-MS, reaching completion after 100 hours of stirring at room temperature. The following step was also carried out on the crude and involved acetamidomethyl (Acm) protecting group removal from Cys3 and Cys9, with concomitant disulfide formation, mediated by iodine.^51^ After 2 hours of reaction, the crude was treated with ice-cold diethyl ether to induce precipitation, and compound 4 was obtained in a satisfactory 20% overall yield (calculated from the initial resin loading) after reverse-phase HPLC purification.

**Scheme 4:**
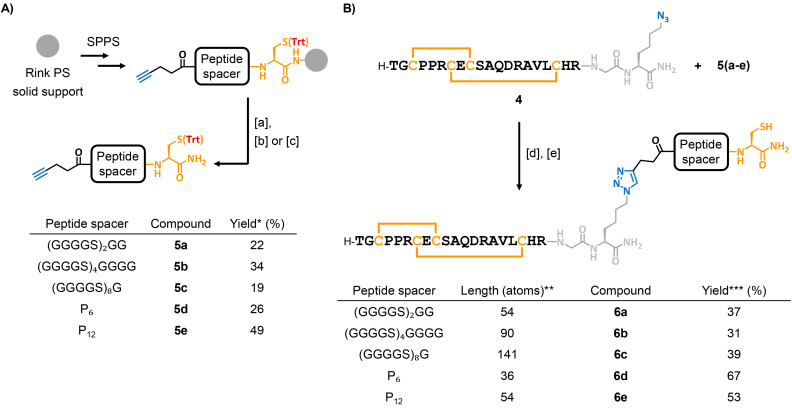
Synthesis of (A) spacers 5a–e, and (B) DRP–spacer–thiol constructs 6a–e. Reagents and conditions: [a] TFA/H_2_O/*i*Pr_3_SiH/phenol – 88 : 5 : 2 : 5, 2 h, 0 °C to r.t.; [b] Trt-OH, HFIP, then, DIPEA. (5a–c); [c] Trt-OH, HFIP (5d–e); [d] CuBr–Me_2_S, THPTA, DIPEA, DMSO, 37 °C; [e] TFA/H_2_O/iPr_3_SiH/phenol/DODT – 83 : 5 : 2 : 5 : 5, 1 h, 0 °C to r.t. Abbreviations: TFA = trifluoroacetic acid; r.t. = room temperature; Trt-OH = triphenylmethanol; HFIP = 1,1,1,3,3,3-hexafluoro-2-propanol; DIPEA = *N*,*N*-diisopropylethylamine; THPTA = *tris*(3-hydroxypropyltriazolylmethyl)amine; DMSO = dimethyl sulfoxide. *Overall yields calculated from resin loading. **Spacer length in atoms separating the spare thiol and the first amino acid of the DRP. ***Yields calculated for step [d]; step [e] was considered quantitative.

For the spacer design, we chose peptide-based linkers, which provide greater modularity in length and structural properties than oligo(ethylene glycol) linkers used in Katayama's approach. Peptide-based spacers are readily synthesized by automated SPPS, with thiol and alkyne groups conveniently introduced through automated coupling of a Cys residue and an alkynoic acid, respectively. We first synthesized the tridecapeptide spacer 5a (Scheme 4), composed of two Gly–Gly–Gly–Gly–Ser repeats, commonly used as flexible linkers in recombinant fusion proteins.^52,53^ This design places 54 atoms between the thiol and the first amino acid of the DRP, *versus* 33 in Katayama's spacer; both linkers display high conformational flexibility.^33^ We synthesized 5a using a standard Rink linker-functionalized polystyrene SPPS resin, incorporating a Trt-protected Cys residue at the C-terminus (Scheme 4A). The spacer sequence was terminated by coupling 4-pentynoic acid at the N-terminus, thereby providing an alkyne handle for subsequent CuAAC reaction with 4. To prevent side reactions, the spacer's Cys residue was kept protected until the thiol–maleimide conjugation stage. Therefore, after peptide cleavage and concomitant deprotection of acid-labile protecting groups, a follow-up treatment with trityl alcohol in hexafluoroisopropanol^54^ chemoselectively afforded Trt-protected spacer 5a. We note that we observed the occurrence of isomeric compounds that we attributed to *N*→*O* acyl shift on serine residues, which likely took place during post-SPPS acidic treatment.^55^ An additional basic treatment after the tritylation step was therefore carried out to revert the ester bonds into native amides (Scheme S2).^56^

**Fig. 2:**
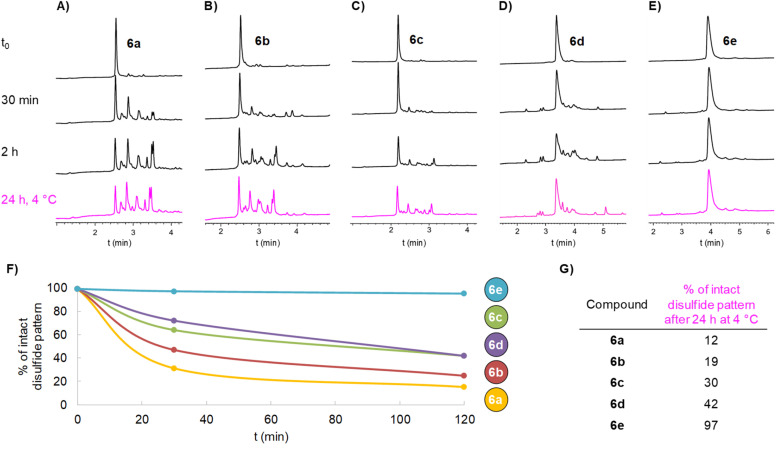
(A–E) RP-HPLC monitoring at 214 nm of disulfide pattern disruption assays for constructs 6a–e at different time points, depicted in black for room temperature assays (*t* = 30 minutes and 2 hours) and magenta for 4 °C assays (*t* = 24 hours). (F) Plot showing percentages of intact disulfide patterns for constructs 6a–e in assays carried out at room temperature; values were obtained by integration of starting material HPLC peaks (214 nm) *versus* newly appeared peaks that were assigned to isomers by LC-MS. (G) Percentage of intact disulfide patterns for constructs 6a–e in assays carried out at 4 °C.

With purified alkyne-functionalized 5a and azido-DRP 4 in hand, we set out to obtain the corresponding “DRP–spacer–thiol” construct by CuAAC reaction. After an initial optimization phase (Table S4), the reaction conditions that gave us the best results involved using an excess of CuBr–Me_2_S as a source of Cu(i), *N*,*N*-diisopropylethylamine as an organic base, tris(3-hydroxypropyltriazolylmethyl)amine (THPTA) as a copper ligand, and DMSO as the solvent (Scheme 4). The purified triazole-ligated product was treated under acidic conditions to unmask the Cys thiol, then precipitated by dilution into diethyl ether to afford 6a. This DRP–spacer–thiol construct was then subjected to disulfide pattern disruption assays (Fig. 2).

Potential disulfide pattern disruption was assessed by incubating aliquots of 6a (50 µM)§§Disulfide pattern disruption assays were conducted under dilute conditions to minimize compound usage, since dilution does not alter the rate of intramolecular reactions. To further validate these observations, the assay was repeated for the best-performing DRP–spacer–thiol construct, 6e, at 1.75 mM, a concentration 2.4-fold higher than that used for conjugation to KLH (0.72 mM, see the SI, p. S80). The results obtained under these conditions were consistent with those observed in assays performed under more dilute conditions. in a classical buffer used for thiol–maleimide coupling: 20 mM phosphate buffer at pH 6.6, containing 150 mM NaCl and 35 mM EDTA,^57^ but in the absence of maleimide reagents. Strictly deoxygenated conditions were used to prevent air-mediated intermolecular disulfide formation. We chose two typical conditions for thiol–maleimide coupling: either 2 hours at room temperature (20 °C), or 24 hours at 4 °C.^57^ The extent of disulfide pattern disruption was monitored by HPLC (Fig. 2A) and MS analyses. The proportion of intact disulfide pattern at different incubation times (Fig. 2F and G) was determined by integrating the UV signal at 214 nm of the starting compound peak relative to newly appeared peaks, which were assigned to disulfide shuffled by-products by LC-MS, having identical molecular mass as 6a.

To our surprise, 6a showed a strong tendency for intramolecular disulfide exchange. Among all disulfide isomers, the species with the native disulfide pattern accounted for only 15% of the mixture after 2 h at 20 °C,¶¶A compound containing 5 cysteines engaged in two intramolecular disulfide bonds can give rise to 14 distinct shuffled isomers. and this dropped to 12% after 24 h at 4 °C. No intermolecular disulfide-linked dimers could be detected by LC-MS, indicating the absence of both intermolecular disulfide shuffling at the tested concentration and oxygen-mediated formation of intermolecular thiol–disulfide bonds, which could otherwise give rise to false positive peaks in the HPLC chromatogram.

We hypothesized that the 54-atom spacer in 6a was too short to sufficiently reduce the effective molarity of thiolate/disulfide interactions and thus failed to slow the intramolecular reaction. To test this, we synthesized two additional DRP–spacer–thiol constructs with longer spacers (Scheme 4). Compounds 6b and 6c, containing 90 and 141 atoms, respectively, between the thiol group and the first amino acid of the DRP, were prepared using the protocols optimized for 6a with comparable efficiency. As expected, disulfide shuffling kinetics slowed with increasing spacer length; however, even the construct displaying the longest spacer, 6c (about twice the size of the DRP itself), was not left unaltered. Most of the construct underwent disulfide shuffling upon incubation: only 42% 6c remained after 2 h at 20 °C, and 30% after 24 h at 4 °C.

As a highly simplified model, an ideal flexible spacer allows the thiolate group to reach any position within a sphere whose radius equals the spacer's fully extended length and is centered at its attachment point on the DRP, thereby encompassing nearby disulfides that can be attacked. The accessible volume increases with the cube of spacer length. The spacer of 6c (141 atoms) is ≈2.6-fold longer compared to that of 6a (54 atoms); in this minimalist model, this would translate into an ≈18-fold increase in accessible volume, corresponding to an 18-fold decrease in effective molarity, and thus an 18-fold reduction in the intramolecular thiolate/disulfide exchange rate. However, our experimental results show a far more modest decrease. This likely indicates an underlying conformational bias, perhaps due to the peptide's tendency to adopt packed conformations mediated either by intra-linker, or linker-DRP interactions, even though such bias is not suggested by the extensive literature on (GGGGS)_*n*_ spacers. In a simplified model of an ideal fully-rigid linker, the thiolate would be restricted to the surface—rather than the volume—of the sphere defined above. We reasoned that this could, in principle, abolish intramolecular disulfide shuffling, provided that the rigid linker is longer than the distance between the attachment point and the disulfides. To test this hypothesis, we turned to rigid linkers as an alternative to flexible ones.

Reviewing the molecular biology literature on fusion proteins,^53^ we found that several peptide sequences have been exploited for their rigidity. In particular, α-helix-forming linkers with a (EAAAK)_*n*_ sequence have been frequently used in the construction of recombinant fusion proteins.^58,59^ As suggested by George and Heringa,^60^ many natural linkers between domains of large multidomain proteins also adopt such α-helical structures. Another class of rigid linkers, used to a much lesser extent in fusion proteins,^61^ are proline-rich sequences, typically (XP)_*n*_, where X can be any amino acid (preferably Ala, Lys, or Glu), which adopt either polyproline I (PPI) or polyproline II (PPII) helix conformations. Such helical peptide linkers are likely only partially rigid and may switch to other conformations.^62^ An interesting alternative are linkers composed solely of repeated proline residues (oligoprolines, Pro_*n*_), which form highly stable PPII helices and are often used as “molecular rulers” or “molecular scaffolds”.^63^ Strikingly, Wennemers and co-workers demonstrated in 2014 how a stretch of six Pro residues is sufficient to form a stable PPII helix, amenable to X-ray crystallography.^64^

We synthesized two additional linkers containing either six (5d) or twelve (5e) consecutive Pro residues, and incorporated them into DRP–spacer–SH constructs 6d and 6e, respectively. In contrast to oligo-GGGGS linkers, oligoprolines behaved well during all steps and showed excellent solubility in both aqueous and organic solvents (*e.g.* MeCN or HFIP). Gratifyingly, under thiol–maleimide conditions, the shortest oligoproline-based spacer in 6d (P_6_), spanning 36 atoms between the DRP and the thiol, gave results comparable to those obtained with the longest flexible linker of our previous series (141 atoms, 6c). Strikingly, in 6e (P_12_ spacer), the disulfide pattern remained nearly intact after 2 hours at room temperature, or 24 hours at 4 °C (Fig. 2E–G), highlighting the extraordinary potential of such a short and simple linker for DRP conjugation.

**Fig. 3:**
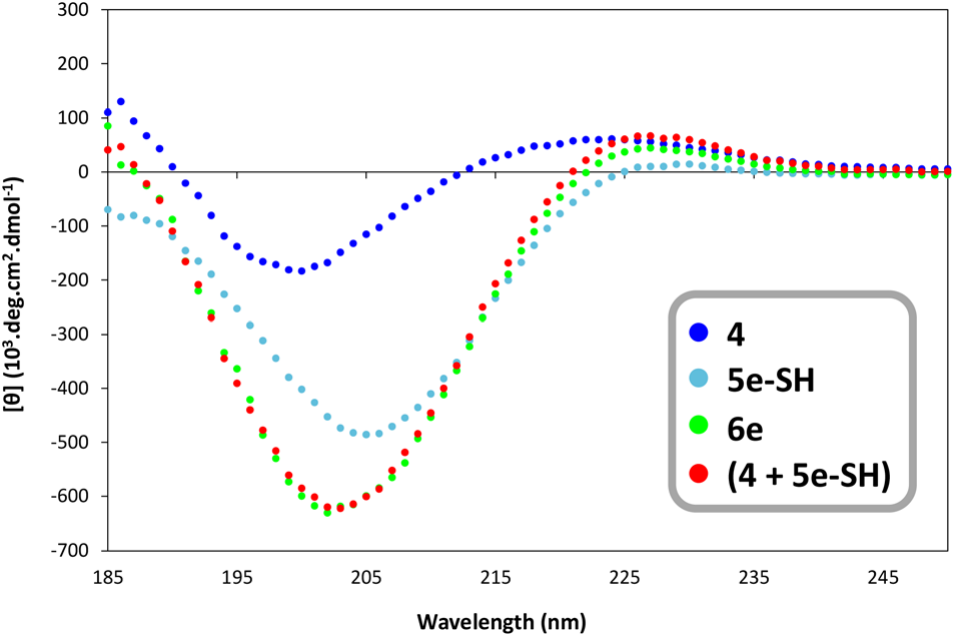
Circular dichroism spectra of compounds 4, 5e-SH, and 6e (50 mM concentration) recorded between 185 and 280 nm at 20 °C. *Sum of experimental values obtained for 4 and 5e-SH.

Having validated 6e as a DRP–spacer–thiol construct of choice, we next performed circular dichroism studies to confirm that the expected rigid PPII-type secondary structure of the oligo-proline spacer was maintained upon conjugation to the DRP and to ensure that its presence did not negatively affect the three-dimensional structure of the DRP antigen. As a first step, we carried out independent measurements on azido-DRP 4 and spacer 5e-SH, corresponding to 5e after trityl group deprotection (Fig. 3).

The CD spectrum of 5e-SH showed the characteristic signature of an oligoproline PPII helix, with a weak positive peak in the 225–235 nm region and a strong negative peak at ≈205 nm (Fig. 3), consistent with literature data.^65^ The red trace in Fig. 3 corresponds to the sum of the experimental spectra obtained for 4 and 5a-SH, representing the theoretical signal expected if both the DRP and oligoproline spacer structures remain unaltered upon covalent attachment. Strikingly, the CD spectrum of construct 6e (green trace) was almost superimposable with this sum, indicating that the PPII helix of the spacer was retained even after chemical conjugation to the DRP.

### *In vivo* immunization and cellular characterization of polyclonal antibodies

**Fig. 4:**
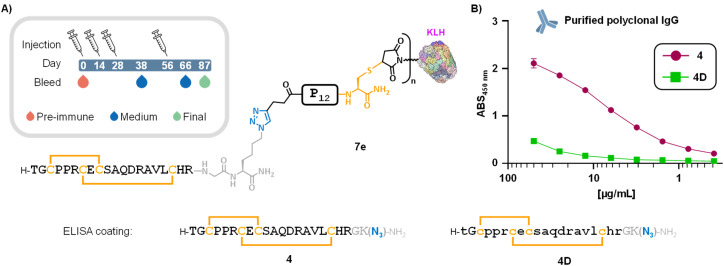
(A) Structure of immunogen 7e, synthesized from construct 6e, and immunization schedule (box). (B) ELISA titration curves of protein A-purified polyclonal IgG for plates coated with 4, and negative control 4D (d-amino acids are indicated by lowercase letters).

We next prepared KLH-based immunogens from 6e for subsequent immunization assays. KLH conjugate 7e (Fig. 4A) was obtained by reacting commercial maleimide-functionalized KLH under a standard thiol–maleimide protocol.||||Such standard conjugation protocol is performed at 0.7 µM of peptide, significantly higher than the concentration used in our disulfide shuffling assays (50 µM). Before embarking in the large-scale conjugation, we have checked the stability of construct 6e at 1.75 mM concentration in the absence of KLH and did not observe any significant trace of by-products after 2 hours of incubation, similarly to our 50 µM disulfide shuffling assays (Fig. 2E). For stoichiometry calculations, we assumed a mean molecular weight of 6 MDa and a mean of 240 maleimide handles per KLH molecule (according to the product specifications), corresponding to 40 nmol of maleimide groups per mg of maleimide-KLH. Following the manufacturer's protocol and using the same buffer reported in our incubation assays, a 9-fold excess of construct 6e (7.7 mg, 1.77 µmol, 8.9 equiv.) per maleimide residue was reacted with 5 mg of maleimide-KLH (Scheme S4). After 2 hours of incubation at room temperature, the reaction mixture was diluted with PBS buffer and subjected to several cycles of centrifugal filtration (30 kDa cutoff Amicon®), enabling buffer exchange to PBS and removal of excess 6e. A total of 470 µg of conjugate 7e were then used in a standard rabbit immunization protocol (Fig. 4A, box).

ELISA titration curves of post-immunization sera showed a progressive increase in antibody responses over time on plates coated with DRP 4 (Fig. S49A). Comparison of sera collected at different time points (D38, D66, and D87) with pre-immune serum (D0) revealed a clear rise in absorbance values at 450 nm, indicating a successful and time-dependent humoral response. Final bleed serum was purified by a protein A matrix (Eurogentec), yielding a 41.8 mL sample of polyclonal IgG at 10 mg mL^−1^ concentration. ELISA assays with polyclonal IgG showed a clear dose–response curve for wells coated with 4 (Fig. 4B). To assess the specificity of the immune response, we included compound 4D (Fig. 4B) as a comparator, in which the LINGO-1[1–20] segment was synthesized from d-amino acids (and achiral Gly), yielding the mirror-image version of the DRP moiety. Owing to its identical sequence and fold presented in the opposite stereochemical configuration, this mirror-image peptide provides a more stringent and structurally faithful negative control than the conventionally used “scrambled” peptides. The negligible binding observed for 4D (Fig. 4B and S49D) confirmed the specificity of the antibody response elicited by immunogen 7e.

Post-immunization serum also bound to wells coated with the P_12_ linker (Fig. S49C). This outcome was expected, as the immune system can generate responses to any foreign epitope within the immunogen, including synthetic linkers, and of course the carrier protein. Although both the carrier protein^66^ and the cross-linker^67^ components can suppress hapten immunogenicity at the immunization stage, the presence of anti-P_12_ and anti-KLH IgG antibodies in our polyclonal pool does not affect the specificity or utility of the generated antibodies.**It is worth mentioning that proline residues constitute ≈6% of amino acids in the human proteome, but only ≈4% of these occurring in stretches of three or more consecutive residues.^73^

**Fig. 5:**
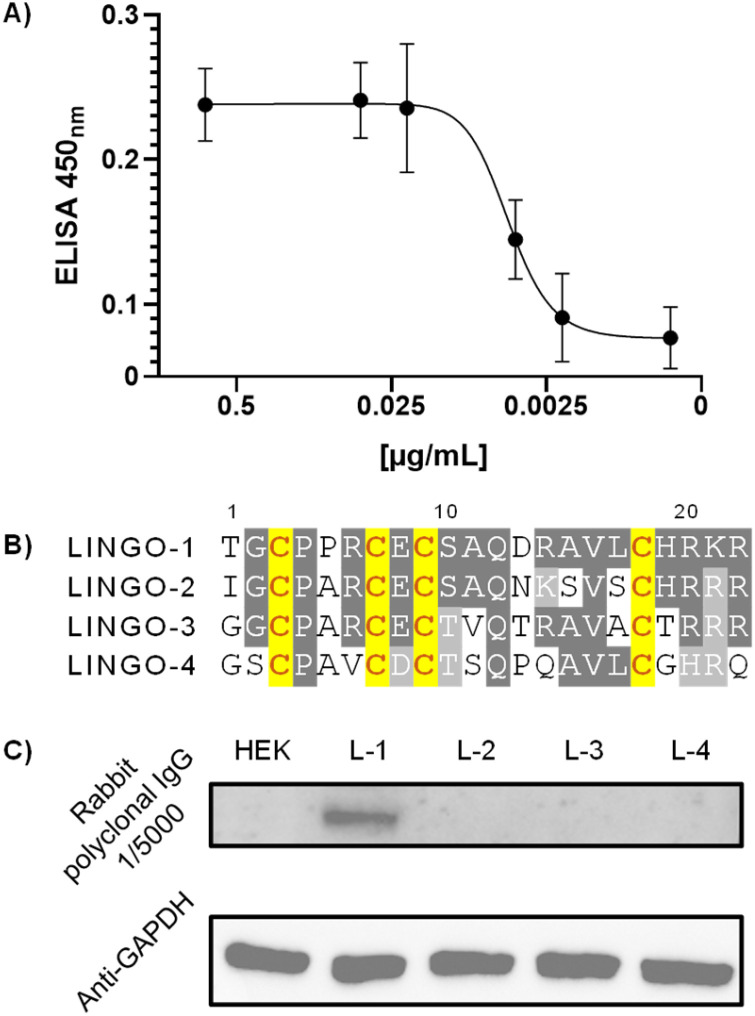
(A) ELISA cell assay showing the interaction between decreasing concentrations of polyclonal IgG and HEK cells expressing LINGO-1. Absorbance values at 450 nm were background-corrected by subtracting the signal from non-transfected HEK cells. (B) Superposition of the [1–20] region for LINGO-1-to-4 homologs shows that all Cys residues are conserved; additional conserved residues are highlighted in dark grey, whereas similar residues—defined by comparable hydrophilicity (*e.g.*, Ser *vs.* Thr) or charge (*e.g.*, Lys, Arg, or His)—are shown in light grey. (C) Western blot detail in which polyclonal IgG was used for staining HEK cells and LINGO homologs 1–4. Anti-GAPDH staining is shown at the bottom as a loading control.

To further assess the quality and utility of our anti-LINGO-1[1–20] polyclonal IgGs, we evaluated their ability to bind LINGO-1 in a cellular context. HEK-293 cells were transiently transfected with a construct expressing LINGO-1, and ELISA cell assays were performed using non-transfected HEK as a negative control (Fig. 5A). The results showed a dose–response curve with absorbance values reaching saturation at ≈25 ng mL^−1^ of polyclonal IgG, indicating that the generated polyclonal IgG efficiently recognizes the full length, folded LINGO-1 protein.

We next evaluated the ability of our polyclonal IgG to detect LINGO-1 by western blotting. Proteins extracted from cell lysates were heated under reducing conditions, then separated by SDS-PAGE and subsequently transferred to polyvinylidene fluoride membranes. Remarkably, our polyclonal IgG detected LINGO-1 at a five-fold higher dilution than the commercial polyclonal IgG antibody AF3086††††In a previous study,^45^ screening of all commercially available IgGs identified polyclonal IgG AF3086 as the most suitable for co-immunoprecipitation studies. (R&D Systems), while also yielding stronger overall staining intensity (Fig. S52 and S53). This indicates that our polyclonal IgG is also able to recognize a reduced and denatured form of the protein.

Finally, we evaluated the specificity of the polyclonal IgG against the four LINGO homologs. Concerning the LINGO-1[1–20] region, sequence similarity for homologs -2, -3 and -4 consists of 75%, 70% and 45% identity, respectively (Fig. 5B). Notably, although only the structure of LINGO-1 has been reported, all four homologs share the same Cys pattern, suggesting a conserved structural fold in this N-cap region (see SI, pp. S19–S22 for homology modelling). HEK-293 cells were transiently transfected to express LINGO homologs 1–4, and expression of all four homologs was confirmed by probing for the C-terminal HA-tag included in each construct (Fig. S54). Western blotting with our polyclonal IgG detected an immunoreactive species between 75 and 100 kDa, corresponding to LINGO-1, with no cross-reactivity for LINGO-2, -3, or -4 (Fig. 5C and S52). Overall, rabbit polyclonal IgG generated by immunization with construct 7e and purified *via* protein A affinity chromatography demonstrated excellent sensitivity in western blot assays and a strong homolog specificity, supporting its utility for the detection of LINGO-1 in overexpression systems and potentially in more complex biological samples.

## Conclusions

Disulfide-rich peptides (DRPs) are a broad class of conformationally-stabilized compounds with important biological functions. They can be harnessed as structural mimics of protein regions and used to elicit antibodies by serving as immunogens. However, small-size DRPs must be conjugated to large carrier proteins to elicit an effective immune response, owing to their inherently low immunogenicity. Thiol–maleimide chemistry is one of the most widely used methods for generating such peptide–protein conjugates, supported by the availability of commercial maleimide-functionalized carriers such as KLH. However, introducing a spare thiol into a DRP is challenging, as it can disrupt the native disulfide pattern and yield heterogeneous immunogens that fail to accurately mimic the intended epitope. In this work, we have established a robust strategy for the late-stage introduction of thiol-containing linkers on DRPs. This enables the generation of “DRP–spacer–thiol” constructs that preserve the original disulfide bridging pattern under thiol–maleimide reaction conditions and are thus suitable for subsequent bioconjugation with maleimide-functionalized protein carriers. We focused on LINGO-1[1–20] as a model DRP, a small peptide with solvent-exposed disulfide bridges that are particularly prone to nucleophilic attack. Using our approach, we generated an effective immunogen that successfully elicited an IgG immune response following a standard immunization protocol.

ELISA assays showed that protein A-purified polyclonal IgG selectively bound the parent LINGO-1 protein on the surface of transfected cells. Western blot assays demonstrated that our polyclonal IgG exhibited superior sensitivity compared to a commercial IgG and showed strong specificity for the LINGO-1 homolog, distinguishing it from homologs -2, -3, and -4, which share the same cysteine pattern and have sequence similarities ranging from 45% to 75%. In conclusion, these results establish a robust strategy for generating DRP-specific antibodies while preserving native disulfide patterns. These findings indicate that our approach can be easily adapted to a variety of DRPs, offering a valuable tool for the research community.

## Author contributions

V. A. conceived the project and acquired funding; V. A. and C. P. coordinated the project; S. M. L. supervised the cellular biology experiments; H. M. performed NMR data acquisition and structure calculation; K. L. supervised NMR structure determination and analysed the NMR structure; L. A., C. P., and J.-B. M. performed the synthesis, purification and characterization of synthetic compounds; L. A. and A. N. performed ELISA assays; L. A. performed circular dichroism experiments; A. N. performed cellular biology experiments; C. P. wrote the first draft and prepared figures with inputs from all authors; L. A., H. M., K. L., S. C., S. M. L., C. P. and V. A. interpreted results, reviewed and edited the manuscript.

## Conflicts of interest

The authors declare no competing interests.

## Supplementary Material

SC-017-D5SC08821F-s001

SC-017-D5SC08821F-s002

SC-017-D5SC08821F-s003

SC-017-D5SC08821F-s004

SC-017-D5SC08821F-s005

## Data Availability

A comprehensive dataset supporting this article has been uploaded as part of the supplementary information (SI). Supplementary information: HPLC traces and MS spectra, as well as atom coordinates of the peptide 3D structure determined by NMR or homology modeling. Raw data (FID NMR, chromatograms, and MS spectra) are available from the authors upon request. See DOI: https://doi.org/10.1039/d5sc08821f.
