# Supplementary material for: A (poly)Pro tip for preserving native disulfide connectivity during thiol–maleimide bioconjugation of disulfide-rich peptides
Source: Chem Sci. 2026 Mar 30;17(21):10559–70. doi: 10.1039/d5sc08821f (PMC13085933; doi:10.1039/d5sc08821f)
Supplement: SC-017-D5SC08821F-s005 [file SC-017-D5SC08821F-s005.pdf]

Supplementary Information (SI) for Chemical Science.
This journal is © The Royal Society of Chemistry 2026

A (poly)Pro tip for preserving native disulfide connectivity during thiol-maleimide bioconjugation of disulfide-rich peptides

Lylia Azzoug,[a] Ana Novak,[a] Hervé Meudal,[a] Jean-Baptiste Madinier,[a] Stéphane Charpentier,[a,b] Karine Loth,[a,b] Séverine Morisset-Lopez,[a] Carlo Pifferi[a,*] and Vincent Aucagne[a,*]

[a]Centre de Biophysique Moléculaire, CNRS UPR 4301, Rue Charles Sadron, 45071 Orléans, France
[b]UFR Sciences et techniques, Université d'Orléans, rue de Chartres, 45067 Orléans, France

E-mail: carlo.pifferi@cnrs-orleans.fr
E-mail: vincent.aucagne@cnrs-orleans.fr

SUPPORTING INFORMATION

Table of contents:

1- General information..3
2- General procedures for solid phase peptide synthesis..4
2-1 Protocol PS1: Peptide elongation..4
2-2 Protocol PS2: Peptide cleavage..4
2-3 Protocol PS3: Tritylation of peptide spacers **5a**-**5c**..4
2-4 Protocol PS4: Tritylation of peptide spacers **5d** and **5e**..4
2-5 Protocol PS5: Copper(I)-catalyzed azide-alkyne cycloaddition reaction..5
2-6 Protocol PS6: Trityl deprotection..5
3- Synthesis and purification of LINGO-1[1-20] (**1**)..6
3-1 SPPS and cleavage of precursor **S1**..6
3-2 Formation of the first SS bridge..8
3-3 Formation of the second SS bridge..10
4- NMR structural characterization of LINGO-1[1-20] peptide **1**..12
4-1 NMR spectroscopy and chemical shift assignment..12
4-2 Structure calculations..15
4-3 Analysis of the selected structures of LINGO-1[1-20]..17
4-4 Homology modeling..19
5- Synthesis and purification of azido peptide LINGO-1[1-20]GK(N_3) (**4**)..23
5-1 SPPS and cleavage of precursor **2**..23
5-2 Formation of the first SS bridge..24

5-3 Formation of the second SS bridge 26
5-4 Stability under oxidative folding conditions 31
6- Synthesis of peptide spacers **5a**-**5e** 32
6-1 Synthesis of peptide spacer **5a** 32
6-2 Synthesis of peptide spacer **5b** 36
6-3 Synthesis of peptide spacer **5c** 40
6-4 Synthesis of peptide spacer **5d** 43
6-5 Synthesis of peptide spacer **5e** 47
6-5 Evidences of *N*-*O* acyl shift products in linkers **5a**-**5c**, and optimization of conditions to reverse it. 51
7- Synthesis of "DRP-spacer-thiol" constructs **6a**-**6e** 54
7-1 Synthesis of **6a** 56
7-2 Synthesis of **6b** 59
7-3 Synthesis of **6c** 64
7-4 Synthesis of **6d** 68
7-5 Synthesis of **6e** 72
8- Structural characterization of the LINGO-1[1-20]-P_{12}C **6e** construct by circular dichroism 76
9- Disulfide pattern disruption evaluation 79
10- Synthesis of KLH conjugate **7e** and immunization 80
10-1 Conjugation of LINGO-1[1-20]-P_{12}C(SH) to KLH 80
10-2 Immunization 81
11- Evaluation of post-immunization serum and polyclonal IgG 81
11-1 Peptide-based ELISA assays 81
11-3 Western blot assays 89
12- References 92

1- General information

All reagents and solvents were used without further purification. High loading Rink-MBHA polystyrene resin (loading: 0.81 mmol/g) was purchased from Sigma Aldrich (St-Quentin-Fallavier, France). Protected amino acids were purchased from Bachem (Bubendorf, Switzerland). HATU was purchased from Matrix Innovation (Québec, Canada). DIPEA was purchased from Carlo Erba (Val-de-Reuil, France). Peptide synthesis grade DMF was obtained from Iris biotech GmbH (Marktredwitz, Germany). All other chemicals were from Sigma Aldrich and solvents from Carlo Erba. Ultrapure water was obtained using a Milli-Q water system from Millipore (Molsheim, France). Polypropylene syringes fitted with polypropylene frits were obtained from Torviq (Niles, MI, USA) and were equipped with PTFE stopcocks bought from Biotage (Upsala, Sweden). HPLC analyses were carried out on a Hitachi Chromaster system equipped with a 5160 pump, a 5430 diode array detector and a 5260 auto sampler and semi-preparative purifications were carried out on a Hitachi LaChrom Elite system equipped with a L-2130 pump, a L-2455 diode array detector and a L-2200 auto sampler. Chromolith High Resolution RP-18e (150 Å, 10 × 4.6 mm, 3 mL/min flow rate) columns were used for analysis, Nucleosil C18 (300 Å, 5 µm, 250 × 10 mm, 3 mL/min flow rate) for purification. Solvents A and B are 0.1 % TFA in H_2O and 0.1 % TFA in acetonitrile (MeCN), respectively. Each gradient was followed by a washing step to elute any compound not eluted during the gradient (e.g. up to 95% B over 0.5 min, then isocratic 95% B for 0.5 min for the HR Chromolith). LC-MS analyses were carried out on an Agilent 1260 Infinity HPLC system, coupled with an Agilent 6120 mass spectrometer (ESI + mode), and fitted with an Aeris Widepore XB-C18 (200 Å, 3.6 µm, 150 × 2.1 mm, 0.5 mL/min flow rate, 60 °C) column. Solvents A' and B' were 0.1 % formic acid in H_2O and 0.1 % formic acid in MeCN, respectively. Gradient: 3% Solv. B' during 0.6 min then 3-50% Solv. B' over 10.8 min. Low resolution MS of pure compounds were obtained using this system. When needed, the multiply-charged envelope was deconvoluted using the charge deconvolution tool in Agilent OpenLab CDS ChemStation software to obtain the average [M] value.

When needed, solvent deoxygenation was performed through four consecutive vacuum/argon cycles.

For yield calculations purposes, the quantities of purified peptides were determined by weighting, considering a molecular mass including trifluoroacetate counter-ions (one per Arg, His, Lys and N-terminal amine of the peptide sequence) but not water content.

2- General procedures for solid phase peptide synthesis

2-1 Protocol PS1: Peptide elongation

Automated Fmoc-based solid phase peptide syntheses (SPPS) were carried out on a Prelude synthesizer from Protein Technologies Inc., using a Rink amide linker-functionalized polystyrene resin. The side-chain protecting groups used were Arg(Pbf), Asp(O*t*Bu), Cys(Trt), Gln(Trt), Glu(O*t*Bu), His(Trt), Ser(*t*Bu) and Thr(*t*Bu). Syntheses were performed on a 0.05 mmol-per-reactor scale. 4-Pentynoic acid and protected amino acids (0.5 mmol, 10 equiv.) in *N,N*-dimethylformamide (DMF) (1 mL) were coupled for 30 minutes using (dimethylamino)-*N*,*N*-dimethyl(3H-[1,2,3]triazolo[4,5-b]pyridin-3-yloxy)methaniminium hexafluorophosphate (HATU) (180 mg, 0.475 mmol, 9.5 equiv.) in DMF (1 mL), *N,N*-diisopropylethylamine (DIPEA) (174 µL, 1 mmol, 20 equiv.) in *N*-methylpyrrolidone (NMP) (1 mL) and additional NMP (1 mL). Capping of possible unreacted amine groups was achieved by treatment with acetic anhydride (286 µL, 3.02 mmol, 60 equiv.), DIPEA (136 µL, 0.8 mmol, 15.5 equiv.) and hydroxybenzotriazole hydrate (HOBt) (12 mg, 0.088 mmol, 1.8 equiv.) in NMP (5 mL) for 7 min. Fmoc group deprotection was carried out by three successive treatments with 20% *v/v* piperidine in NMP (4 mL) for 3 min.

2-2 Protocol PS2: Peptide cleavage

The peptidyl resin was thoroughly washed with dichloromethane (CH_2Cl_2), dried under vacuum, and then treated with trifluoroacetic acid (TFA)/H_2O/*i*Pr_3SiH/phenol/3,6-dioxa-1,8-octanedithiol (DODT), 83:5:2:5:5 *v/v/v/w/v* for 2 h. The peptide was precipitated by dilution into an ice-cold diethyl ether/petroleum ether 1:1 mixture, recovered by centrifugation and washed with diethyl ether.

2-3 Protocol PS3: Tritylation of peptide spacers 5a-5c

The peptide spacer (10 µmol) and trityl alcohol (3.12 mg, 12 µmol, 1.2 equiv.) were weighed in a vial and solubilized in 1 mL 1,1,1,3,3,3-hexafluoro-2-propanol (HFIP). The reaction mixture was stirred at room temperature and monitored by HPLC and LC-MS until completion, or until no further evolution was observed. Then, 100 µL of DIPEA were added to reverse Ser *N*→*O* acyl shift. After overnight stirring at room temperature, the solvent was evaporated under a stream of air, and dimethyl sulfoxide (DMSO) (5 mL) was added to the crude product. The resulting fine suspension was centrifuged, the supernatant collected, and the pellet resuspended in a mixture of 6 M guanidinium chloride (Gu·HCl)/MeCN/acetic acid (AcOH) (75:20:5 *v/v/v*) and the resulting solution and the collected supernatant were purified by semi-preparative reverse-phase HPLC.

2-4 Protocol PS4: Tritylation of peptide spacers 5d and 5e

The peptide spacer (10 µmol) and trityl alcohol (3.12 mg, 12 µmol, 1.2 equiv.) were weighed in a vial and solubilized in 500 µL HFIP. The reaction mixture was stirred at room temperature and monitored by HPLC-MS until completion or maximum conversion. The solvent was evaporated under a stream of air, and the crude product was solubilized in a 7:3 water/MeCN mixture containing 0.1% TFA. The resulting fine suspension was filtered and purified by reverse-phase preparative HPLC.

2-5 Protocol PS5: Copper(I)-catalyzed azide-alkyne cycloaddition reaction

Under argon atmosphere, a solution of copper(I) bromide-dimethyl sulfide complex ($CuBr\cdot Me_2S$, 0.69 mg, 3.35 µmol, 5 equiv.) and *tris*(3-hydroxypropyltriazolylmethyl)amine (THPTA) (2.91 mg, 6.7 µmol, 10 equiv.) in 200 µL of deoxygenated DMSO was prepared. This solution was added to a 2 mL microcentrifuge tube sealed with a rubber septum containing the azide-functionalized peptide **4** (2 mg, 0.67 µmol) and the alkyne spacer (1 µmol, 1.5 equiv.). Finally, DIPEA (0.57 µL, 3.35 µmol, 5 equiv.) was added, and the resulting suspension was stirred for 2 hours at 37 °C. The reaction mixture was monitored by HPLC-MS. Upon total conversion of the azide-functionalized peptide, a solution of ethylenediaminetetraacetic acid disodium salt dihydrate (EDTA, 2.49 mg, 6.7 µmol, 10 equiv.) in 200 µL of water was added, and the reaction purified by semi-preparative reverse-phase HPLC.

2-6 Protocol PS6: Trityl deprotection

Trityl-protected peptides were treated with a solution of TFA/H_2O/*i*Pr_3SiH/phenol/DODT (83/5/2/5/5, *v/v/w/v/v*) for 1 hour at room temperature. Then, peptides were precipitated by dilution into an ice-cold 1:1 diethyl ether/petroleum mixture. After centrifugation, the resulting pellet was washed twice with diethyl ether. Peptides were used for disulfide pattern disruption assays or KLH conjugation without any further purification.

3- Synthesis and purification of LINGO-1[1-20] (1)

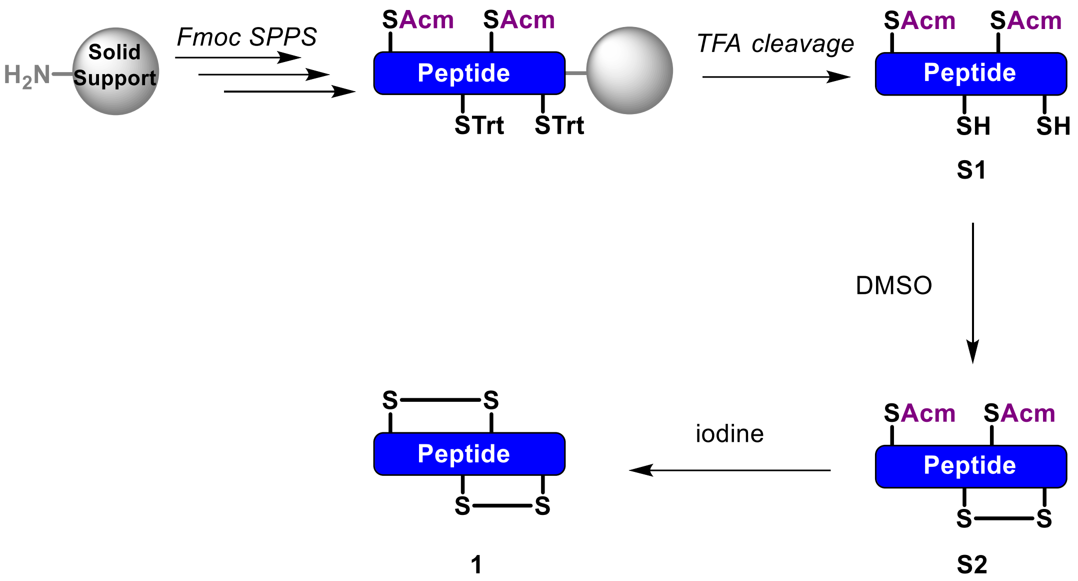

Scheme S1. General protocol for the controlled formation of disulfide bridges using orthogonal protecting groups.

3-1 SPPS and cleavage of precursor S1

Sequence: H-TG**C(Acm)**PPRCE**C(Acm)**SAQDRAVLCHR-NH$_2$ (**S1**)

The peptide was synthesized using protocol PS1. Cleavage was performed following protocol PS2.

ESI-MS: [M] average mass calculated for $C_{100}H_{168}N_{40}O_{31}S_4$: 2343.7, found: 2343.2 (deconvoluted).

HPLC purification: The crude peptide was used without any further purification.

HPLC analysis: t_R = 2.61 min. (Chromolith, gradient: 10-30% Solv. B over 5 min.).

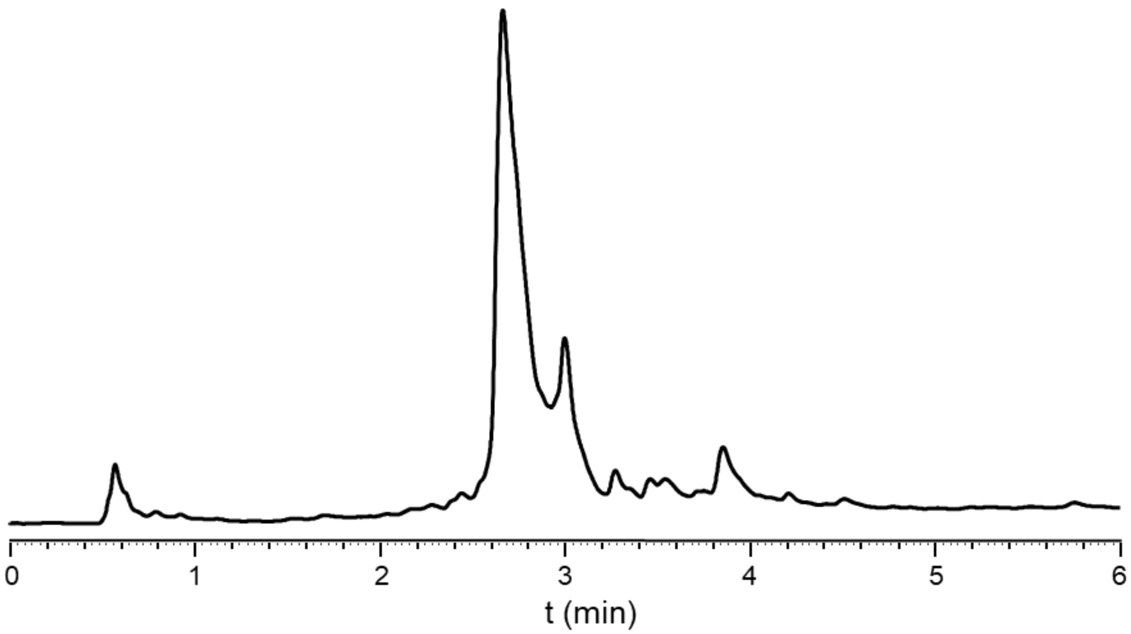

Figure S1. HPLC trace of crude compound **S1** (λ = 214 nm).

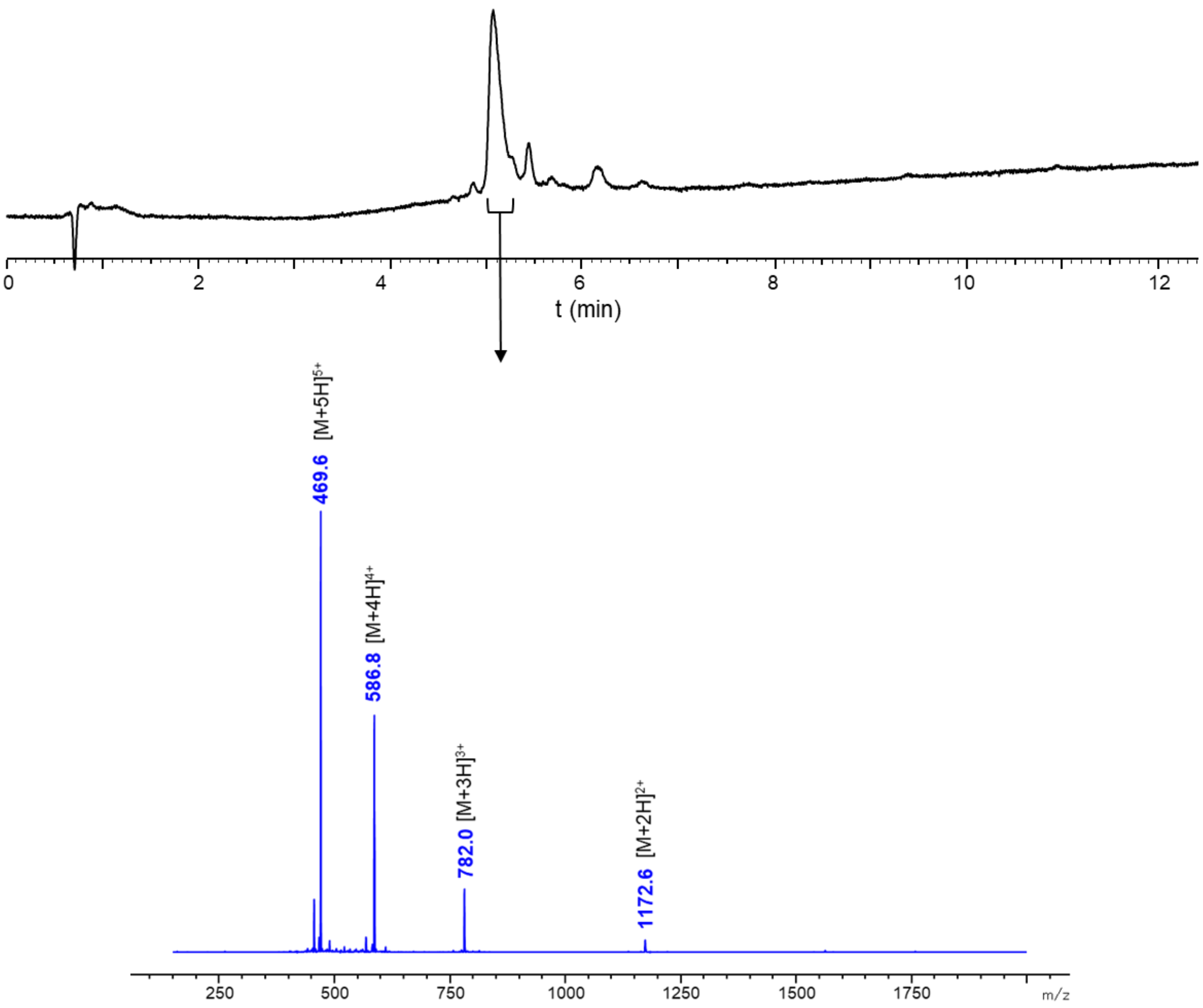

Figure S2. LC-MS analysis of crude compound **S1**: A) HPLC trace (λ = 214 nm) and B) ESI-MS spectrum corresponding to the time window indicated as a black arrow (sum of spectra, 4.68-4.92 min).

3-2 Formation of the first SS bridge

Sequence: H-TG**C(Acm)**PPRCE**C(Acm)**SAQDRAVLCHR-NH_2 (**S2**)

To a stirred solution of LINGO-1[1-20] crude peptide **S1** (43 mg, 14.8 µmol) in 38.7 mL of H_2O, 4.3 mL of DMSO were added dropwise, reaching a final concentration of about 1 mg/mL. The reaction mixture was stirred at room temperature and monitored by HPLC. After 96 hours, the reaction mixture was diluted with water and freeze-dried. Four freeze-drying cycles were performed to completely remove DMSO and obtain a white fluffy powder which was purified by semi-preparative reverse-phase HPLC.

ESI-MS: [M] average mass calculated for $C_{100}H_{166}N_{40}O_{31}S_4$: 2341.7, found: 2340.9 (deconvoluted).

HPLC purification: Nucleosil C18, gradient: 15-25% B over 20 min.; 33% yield (calculated from the 43 mg of weighted peptide starting material, not considering its purity).

HPLC analysis: t_R = 2.86 min. (Chromolith, gradient: 10-30% Solv. B over 5 min.).

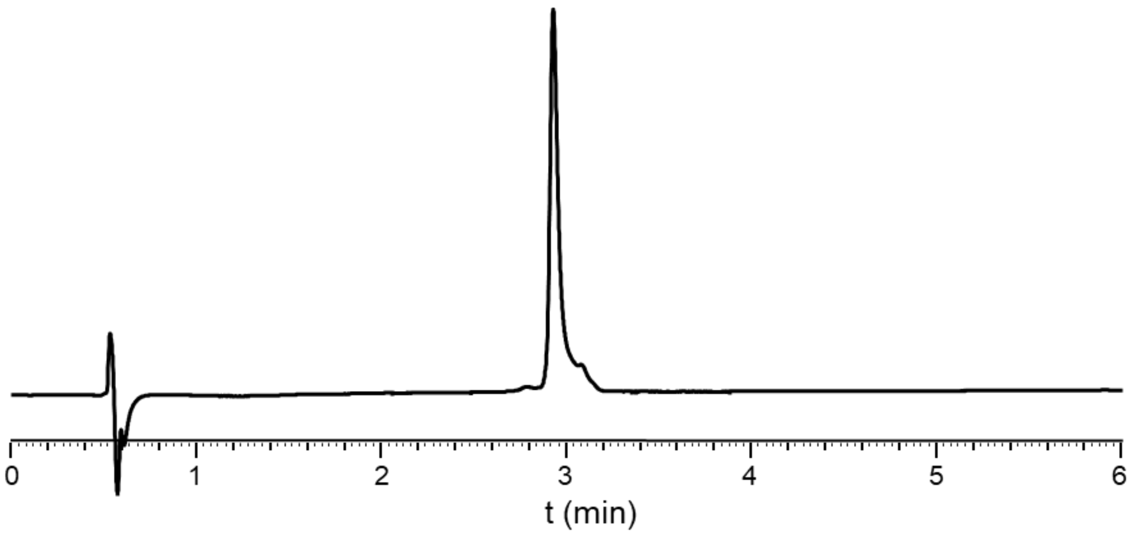

Figure S3. HPLC traces of purified compound **S2** (λ = 214 nm).

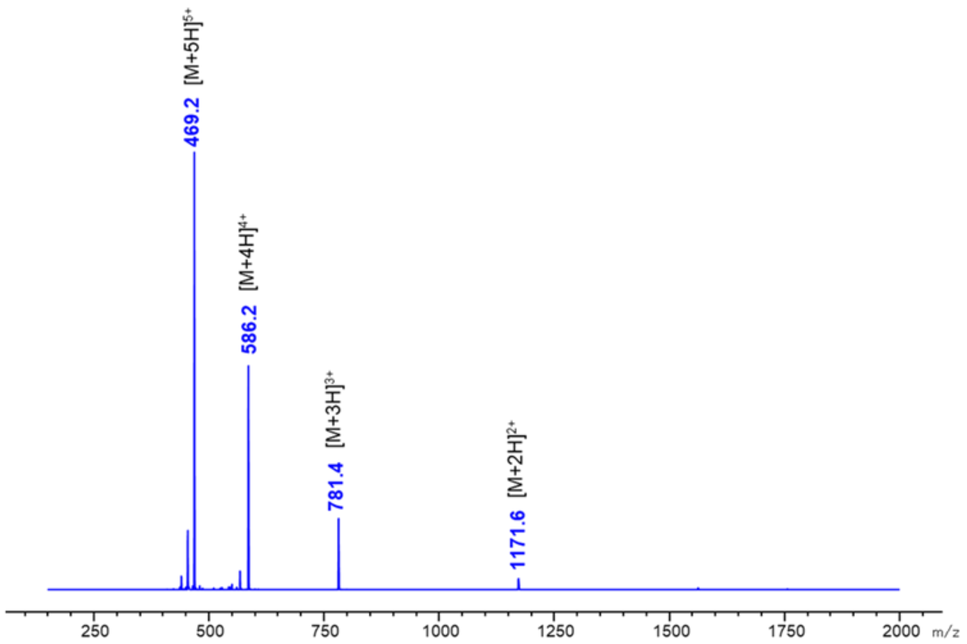

Figure S4. ESI-MS spectrum of purified compound **S2** .

3-3 Formation of the second SS bridge

Sequence: H-TGCPPRCECSAQDRAVLCHR-NH_2 (**1**)

Peptide **S2** (2.75 µmol) was solubilized in 19.7 mL glacial AcOH. Then, 7.7 mL of a 20 mM iodine solution in AcOH, and 1.5 mL of 60 mM aqueous HCl were added. The reaction mixture was stirred at room temperature for 20 minutes, then quenched by adding 260 mL of ice-cooled ether (9 volumes with respect to the reaction volume), and kept for 15 min. at -80 °C to maximize precipitation. The resulting suspension was centrifuged at 4300 rpm for 5 min., the supernatant decanted off, and the resulting pellet crushed and washed with diethyl ether (2 x 50 mL). The resulting yellowish solid was taken into an 8:2 water/MeCN mixture containing 0.1% TFA and purified by semi-preparative reverse-phase HPLC.

ESI-MS: [M] average mass calculated for $C_{94}H_{154}N_{38}O_{29}S_4$: 2197.5, found: 2197.0 (deconvoluted).

HPLC purification: Nucleosil C18, gradient: 10-20% Solv. B over 20 min.; 46% yield.

HPLC analysis: t_R = 2.58 min. (Chromolith, gradient: 10-30% Solv. B over 5 min.).

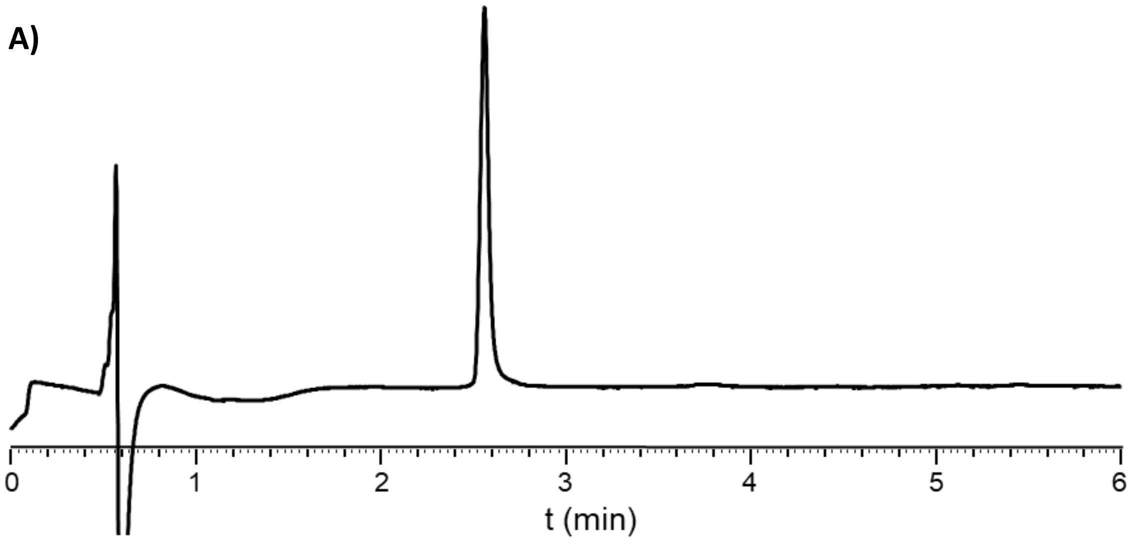

B)

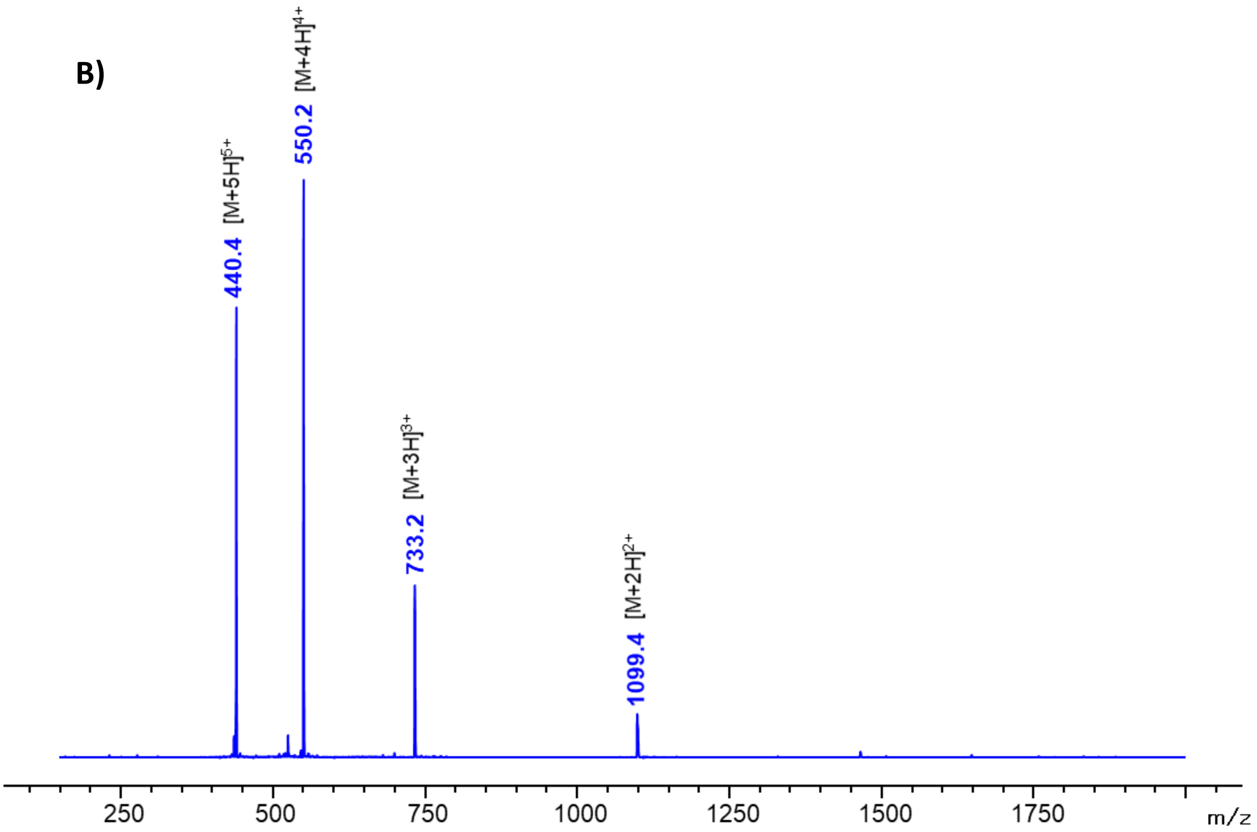

Figure S5. HPLC trace (A, λ = 214 nm) and ESI-MS spectrum (B) of purified compound **1**.

4- NMR structural characterization of LINGO-1[1-20] peptide 1

NMR Sample:

LINGO-1[1-20] (**1**) was dissolved in H_2O:D_2O (9:1 ratio) at a concentration of 1 mM (pH adjusted to 4.5).

4-1 NMR spectroscopy and chemical shift assignment

1D-^{1}H (Figure S6), 2D ^{1}H NOESY, 2D ^{1}H TOCSY, a sofast-HMQC[1] (^{15}N natural abundance) and a ^{13}C-HSQC (^{13}C natural abundance) were performed at 298K on an Avance III HD BRUKER 700 MHz spectrometer equipped with a cryoprobe. NMR data were processed using Bruker's Topspin 3.2™ and analyzed with CCPNMR (version 2.2.2).[2]

The ^{1}H NMR and the sofast-HMQC spectra of the protein revealed a good dispersion of the amide chemical shifts indicative of a structured peptide. Following standard procedures, the analysis of the set of 2D-TOCSY and NOESY spectra combined with the use of the ^{15}N-HMQC and ^{13}C-HSQC allowed the assignment of almost all the observable ^{1}H, ^{13}C and ^{15}N chemical shifts (see Table S1 and Figure S7 and S8).

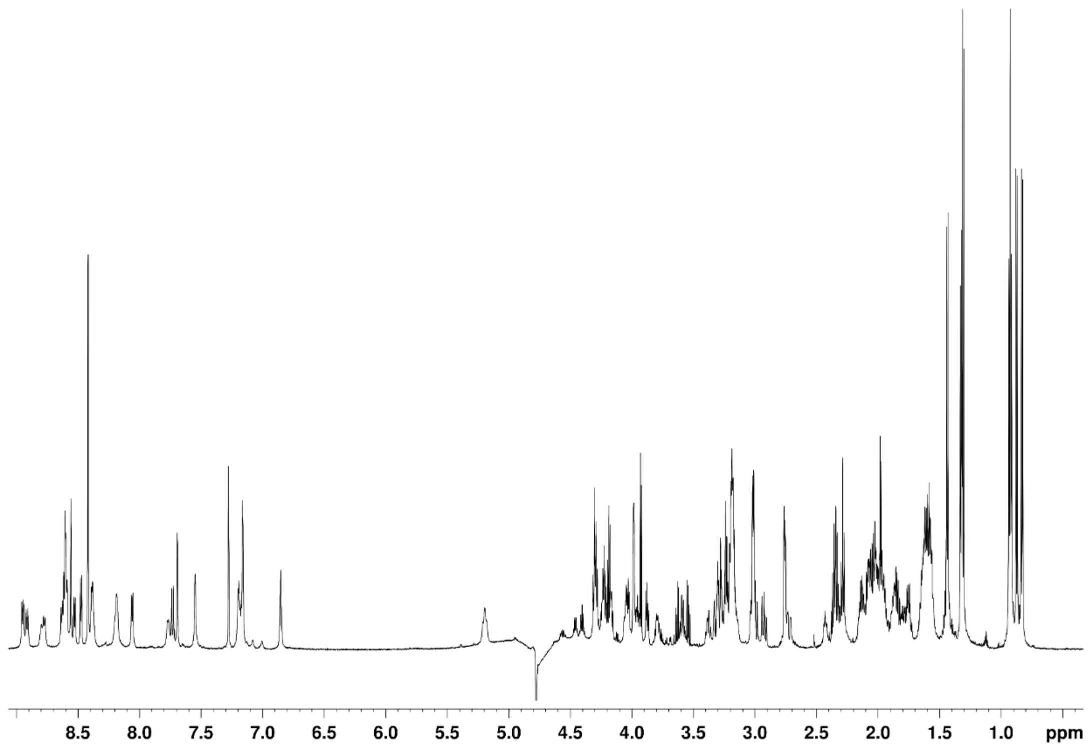

Figure S6. 1D- ^{1}H spectrum of LINGO-1[1-20] **1**.

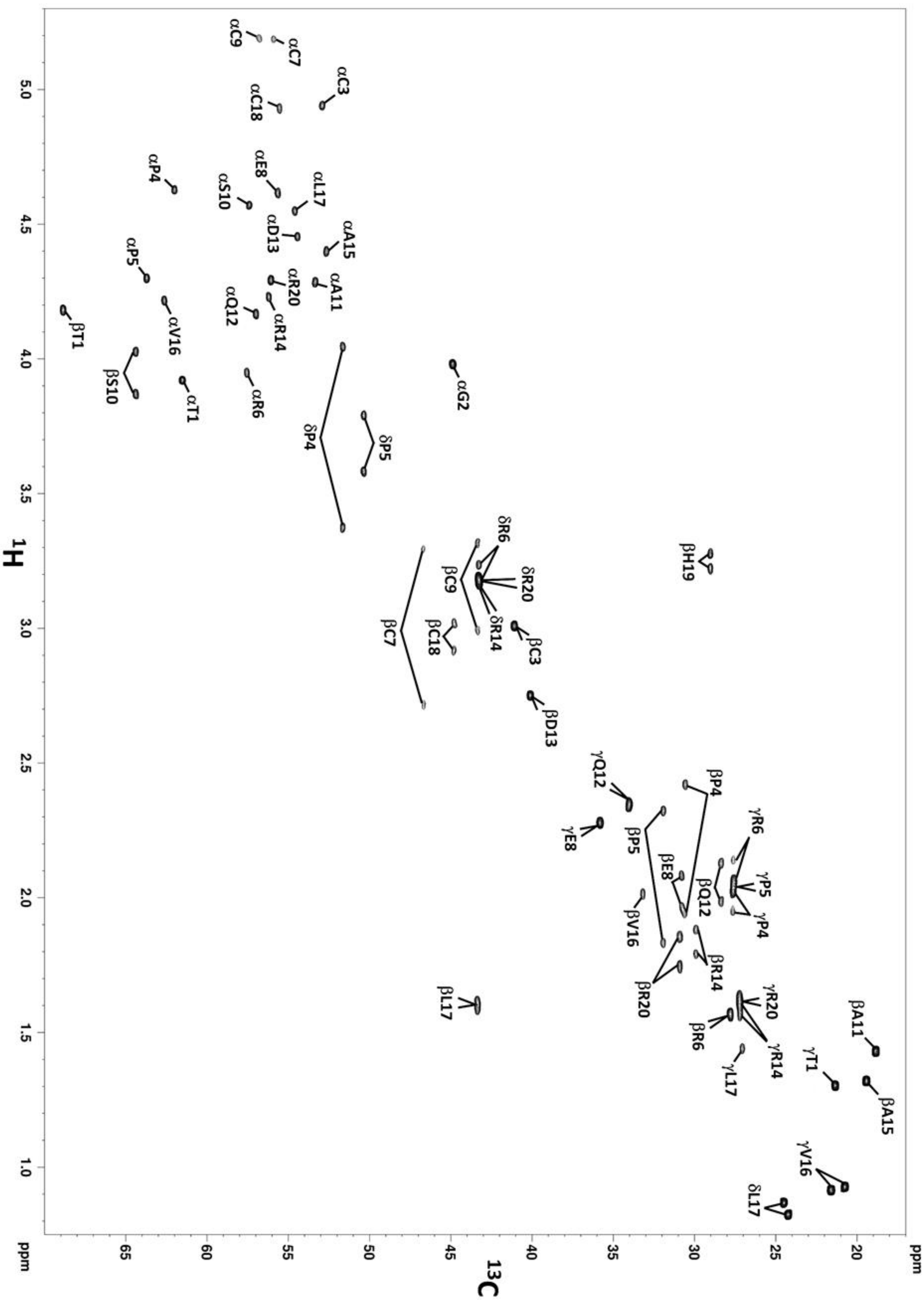

Figure S7. ^{13}C-HSQC of LINGO-1[1-20] **1**.

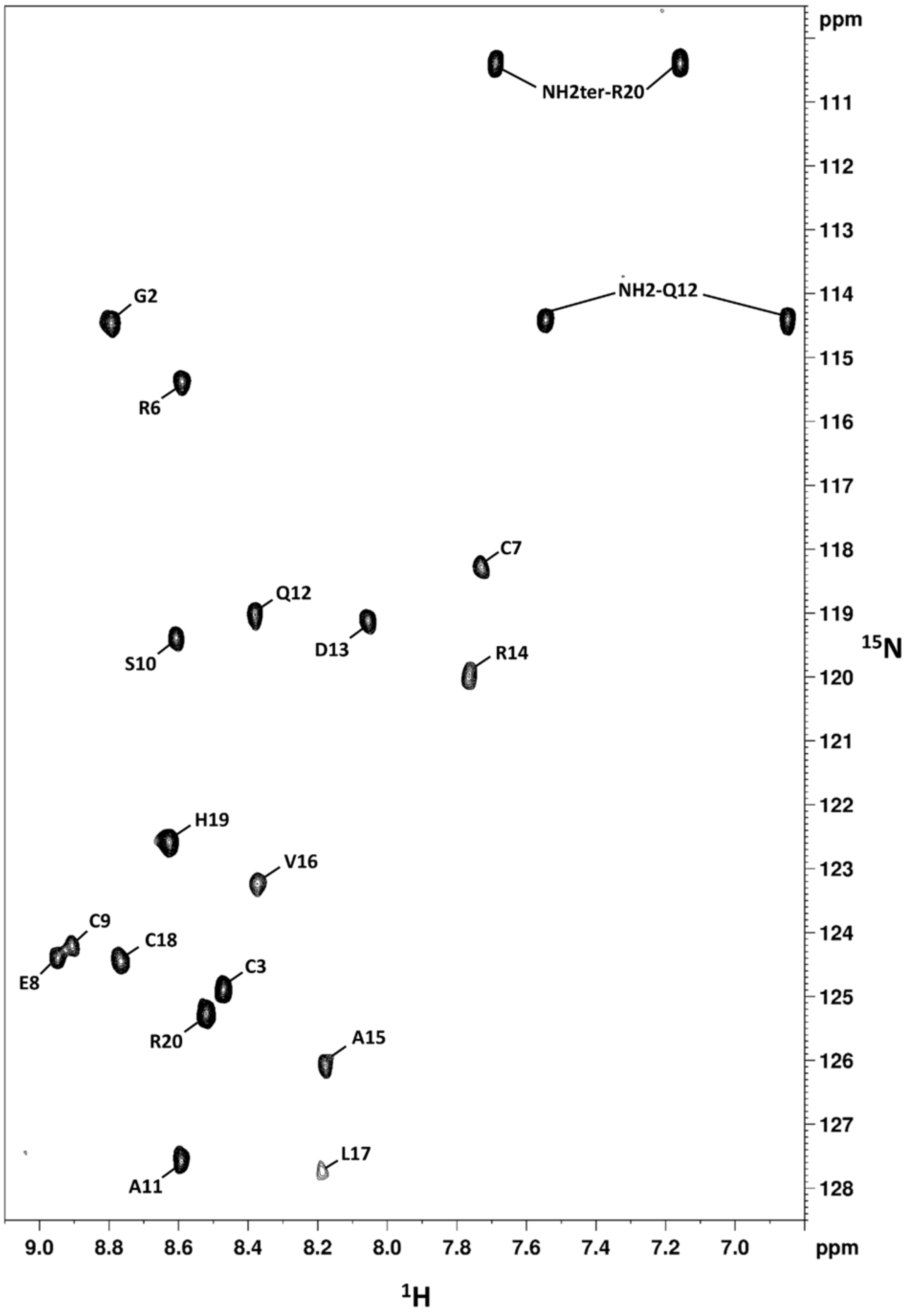

Figure S8. ^{15}N-SOFAST-HMQC of LINGO-1[1-20] **1**.

Table S1. Chemical shifts of LINGO-1[1-20]. Aar is the three-letter code for Arg-NH_2.

	HN	N	Ha	Ca	Others
1 Thr			3.92	61.57	H^{b} 4.17 H^{g2*} 1.31 C^{b} 68.91, C^{g2} 21.38
2 Gly	8.79	114.48	3.98<br>3.98	44.92	
3 Cys	8.47	124.91	4.94	52.95	H^{ba} 3.01 H^{bb} 3.01 C^{b} 41.13
4 Pro			4.63	62.06	H^{ba} 1.94 H^{bb} 2.42 H^{ga} 1.94 H^{gb} 2.02 H^{da} 3.37 H^{db} 4.04, C^{b} 30.64, C^{g} 27.66
5 Pro			4.30	63.76	H^{ba} 1.83 H^{bb} 2.32 H^{ga} 2.05 H^{gb} 2.05 H^{da} 3.58 H^{db} 3.79, C^{b} 31.97, C^{g} 27.63, C^{d} 50.41
6 Arg	8.59	115.39	3.95	57.59	H^{ba} 1.57 H^{bb} 1.57 H^{ga} 2.06 H^{gb} 2.13 H^{da} 3.18 H^{db} 3.23, H^{e} 7.19 C^{b} 27.83, C^{g} 27.65, C^{d} 43.32
7 Cys	7.72	118.29	5.19	55.97	H^{ba} 2.72 H^{bb} 3.29 C^{b} 46.73
8 Glu	8.94	124.41	4.62	55.69	H^{ba} 1.97 H^{bb} 2.08 H^{ga} 2.28 H^{gb} 2.28 C^{b} 30.82, C^{g} 35.86, C^{d} 51.69
9 Cys	8.90	124.22	5.19	56.84	H^{ba} 3.00 H^{bb} 3.31 C^{b} 43.41
0 Ser	8.60	119.40	4.57	57.44	H^{ba} 3.87 H^{bb} 4.03 C^{b} 64.44
1 Ala	8.60	127.60	4.29	53.39	H^{b*} 1.43 C^{b} 18.88
2 Gln	8.38	119.04	4.17	57.04	N^{e2} 114.43, H^{ba} 1.99 H^{bb} 2.13 H^{ga} 2.34 H^{gb} 2.34 H^{e2a} 6.85, H^{e2b} 7.54 C^{b} 28.40, C^{g} 34.07
3 Asp	8.05	119.13	4.45	54.48	H^{ba} 2.75 H^{bb} 2.75 C^{b} 40.15
4 Arg	7.76	120.00	4.23	56.24	H^{ba} 1.79 H^{bb} 1.88 H^{ga} 1.57 H^{gb} 1.61 H^{da} 3.17 H^{db} 3.17, H^{e} 7.16 C^{b} 29.96, C^{g} 27.25, C^{d} 43.32
5 Ala	8.18	126.11	4.40	52.70	H^{b*} 1.32 C^{b} 19.46
6 Val	8.37	123.25	4.22	62.68	H^{b} 2.01 H^{ga*} 0.92 H^{gb*} 0.92 C^{b} 33.23, C^{ga} 21.63, C^{gb} 20.79
7 Leu	8.19	127.75	4.55	54.64	H^{ba} 1.60 H^{bb} 1.60 H^{g} 1.44 H^{da*} 0.82 H^{db*} 0.87 C^{b} 43.39, C^{g} 27.09, C^{da} 24.28, C^{db} 24.53
8 Cys	8.77	124.46	4.93	55.59	H^{ba} 2.92 H^{bb} 3.01 C^{b} 44.83
9 His	8.63	122.60	4.75		H^{ba} 3.23 H^{bb} 3.28 H^{d2} 7.27 H^{e1} 8.55 C^{b} 29.07, C^{d2} 120.41, C^{e1} 136.51
0 Aar	8.52	125.28	4.29	56.12	N^{t} 110.41, H^{nt1} 7.69 H^{nt2} 7.16 H^{ba} 1.75 H^{bb} 1.86 H^{ga} 1.61, H^{gb} 1.63 H^{da} 3.18 H^{db} 3.18 H^{e} 7.19 C^{b} 30.95, C^{g} 27.26, C^{d} 43.32

4-2 Structure calculations

Structures were calculated using CNS[3,4] through the automatic assignment software ARIA2 (version 2.3)[5] with NOE derived distances, 3 hydrogen bonds in accordance with the observation of typical long or medium distance NOE cross peaks network for β-sheets – H^N/H^N, H^N/H^α, H^α/H^α (E8H^N-N17O, N17H^N-E8O and S10H^N-A15O). In a first set of structure calculations, disulfide bonds were treated using ambiguous restraints. Cysteine residues were defined in their deprotonated form, and sulfur–sulfur distance restraints compatible with disulfide bond formation were applied while allowing all possible cysteine pairing combinations. This strategy was used to assess whether the experimental restraints were sufficient to drive the system toward a unique disulfide connectivity without imposing a priori covalent links. Based on the results of these initial calculations, a second set of structure calculations was

performed in which the native disulfide bridges (Cys3–Cys9 and Cys7–Cys18) were explicitly imposed as covalent bonds (Figure S9). The arginine carboxamide (R-NH_2 / Aar) located at the C-terminal position of LINGO-1[1-20] is considered as nonstandard residues in CNS. Topology libraries (topalldg5.3.pro and topalldg5.3.pep) were modified as described in the ARIA 2.3 tutorials. The ARIA2 protocol used simulated annealing with torsion angle and Cartesian space dynamics with the default parameters. The iterative process was repeated until the assignment of the NOE cross peaks was complete. The last run for each peptide was performed with 500 initial structures and 250 structures were refined in water. Ten structures were selected on the basis of total energies and restraint violation statistics (see Table S2), to represent the structure of the peptides in solution. The quality of final structures was evaluated using PROCHECK-NMR.[6] Coordinates of the 10 NMR structures are available in text format as a separated file (.pdb file extension).

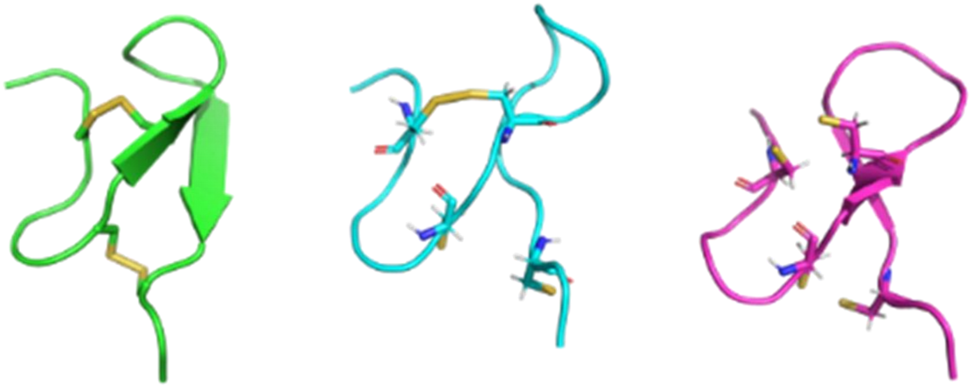

Figure S9. Illustration of the impact of disulfide bond restraints on the solution structure of LINGO-1[1-20]. Left: Representative structure from calculations in which the native disulfide bridges (Cys3–Cys9 and Cys7–Cys18) are explicitly imposed as covalent bonds. Middle and left : representative structure obtained from calculations using ambiguous disulfide restraints, in which cysteine residues are defined as deprotonated and sulfur–sulfur distance restraints compatible with disulfide bond formation are applied while allowing all possible cysteine pairing combinations. In all cases, the global fold is conserved, and cysteine side chains are positioned in geometries compatible with the native disulfide connectivity.

Table S2. NMR restraints and structural statistics for LINGO-1[1-20]. [a]: imposed C3-C9 and C7-C18 ; [b]: determined by PROCHECK on the standard residues of the sequences (N-terminus, C-terminus, proline and glycine are excluded) / values are given as mean ± standard deviation (n=10) ; [c]: values are given as mean ± standard deviation (n=10).

**NMR restraints**	
**Distance restraints**	
Total NOE	***283***
Unambiguous	200
Ambiguous	83
Hydrogen bonds	3
**Disulfide bridges[a]**	2
**Structural Statistics**	
**Average violations per structure**	
NOEs > 0.5 Å	0
Hydrogen bonds > 0.3 Å	0
Average pairwise rmsd (Å)	0.50
**Ramachandran Analysis[b]**	
Most favored region	53.3%
Additional allowed regions	38.7%
Generously allowed	8%
Disallowed	0%
**Energies (kcal.mol^{-1})[c]**	
Electrostatic	-584 +/- 22
Van der Walls	-136 +/- 5
Total energy	-519 +/- 24

4-3 Analysis of the selected structures of LINGO-1[1-20]

Analysis of the selected structures was performed using PDBsum(11). The structure representations were prepared with PYMOL.[7]

The NMR structures of the synthesized LINGO-1[1-20] peptide **1** reveal a typical β-hairpin characterized by a short antiparallel β-sheet composed of two β-strands (E8-S10) precisely matching the β-sheet observed in the XR structure of the Ncap of LINGO-1. Additionally, the N-terminal region folds back onto the β-hairpin due to the presence of a disulfide bridge, forming a rigid β-turn, containing 2 consecutive prolines, between residues P4 and C7 (Figure S10).

Overall, this structural arrangement strongly correlates with the Ncap structure obtained via X-ray crystallography (RMSD = 1.93 ± 0.78 Å), confirming that the synthesized peptide accurately mimics the native structural motif. However, the loop connecting the two β-strands demonstrates orientation variations, suggesting that this region exhibits inherent flexibility and potential dynamic behavior, likely contributing to functional adaptability (see Figure S9).

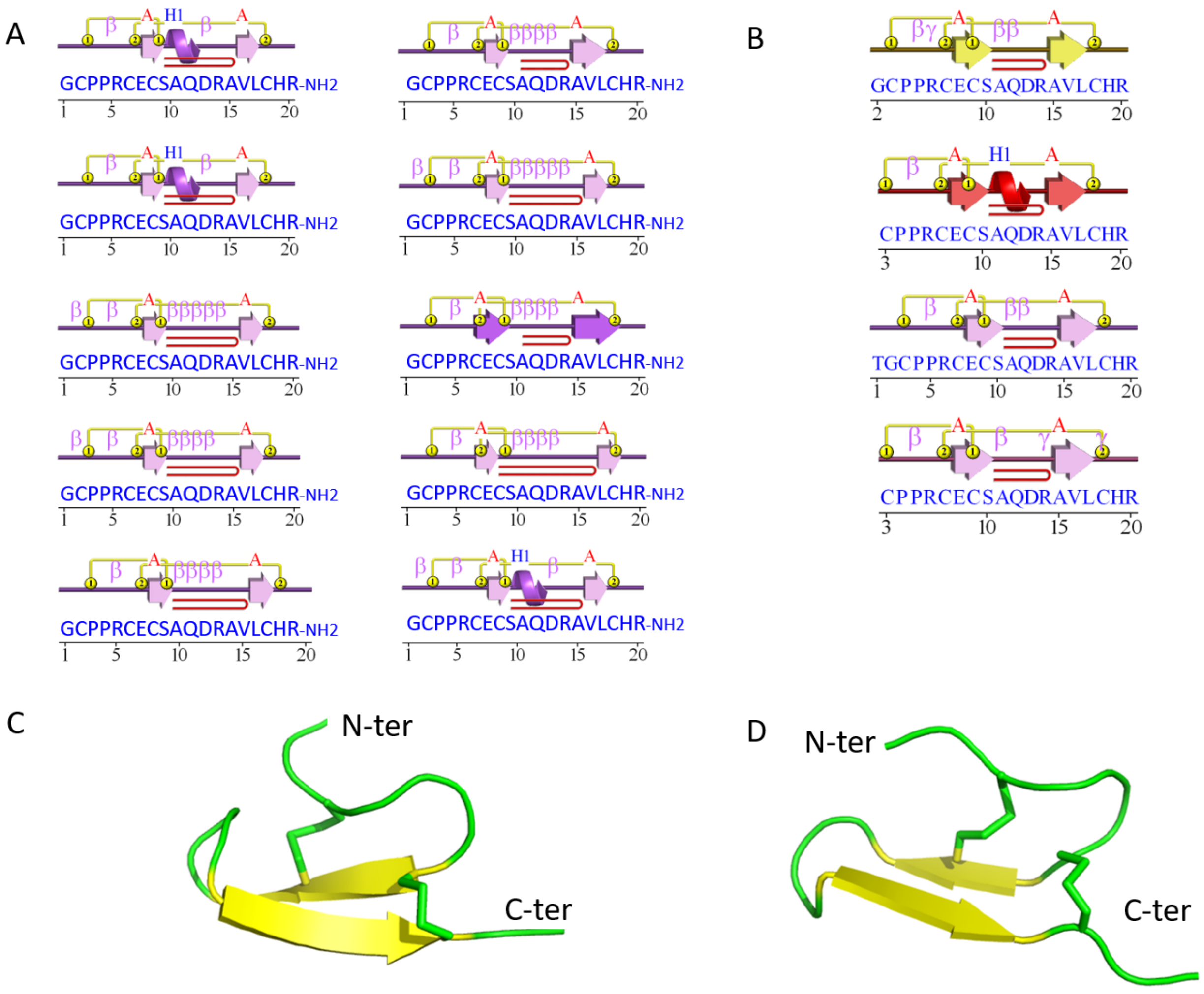

Figure S10. Structural representations of secondary structures and corresponding three-dimensional folds of LINGO-1[1-20] **1**. A/ Secondary structure elements of the 10 selected NMR structures. B/ Secondary elements of residues 1-20 (chains A, B, C, D from top to bottom) extracted from PDB 25ID. β-strands, α-helices, and disulfide bonds are represented as arrows, cylinders and yellow lines, respectively. β-turns are indicated by pink β and the presence of a β-hairpin is indicated. C/ Cartoon representation of the 3D NMR structure of LINGO-1[1-20] D/ Cartoon representation of the Ncap of LINGO-1 XR structure (PDB code 2I5D, chain A)]. β-strands are shown as yellow arrows.

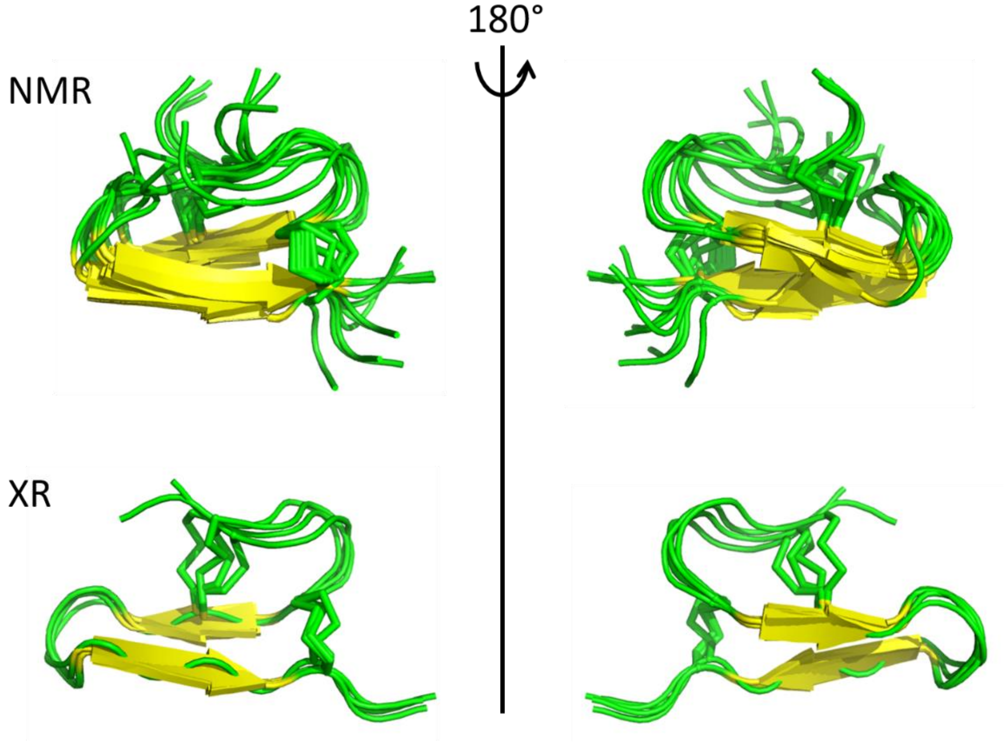

Figure S11. Top: Overlay of the cartoon representation of the 10 selected 3D NMR structures of LINGO-1[1-20] **1** (RMSD = (1.63 ± 1.31) Å); Bottom: Overlay of the cartoon representation of the Ncap [1-20] (chain A, B, C and D – RMSD =(0.87 ± 0.37) Å) of LINGO-1 XR structure (PDB code 2I5D). β-strands are represented in yellow.

4-4 Homology modeling

LINGO-2[1-35], LINGO-3[1-35] and LINGO-4[1-35] were generated through the Robetta webserver with the comparative modelling method[8] using PDB 2I5D as template. The [1-20] part and amidated manually before being analyzed using PDBsum.[9] The structural models and surfaces representations were prepared with PYMOL[7] and ChimeraX.[10] Coordinates of the 3 models are available in text format as separated files (.pdb file extensions).

All peptides adopt a conserved β-hairpin structure consisting of two antiparallel β-strands connected by a loop (Figure S12). This structural motif matches closely with the native structure of LINGO-1[1-20]. Structurally, the mutated residues compared to LINGO-1[1-20] are positioned in distinct regions of the β-hairpin motif. In LINGO-2[1-20], the mutations are located in the N-terminal region and within the second β-strand, without affecting the β-turn or the loop. In LINGO-3[1-20], mutations are distributed across both β-strands and the β-turn, with one substitution occurring within the central part of the loop. In LINGO-4[1-20], the mutations are concentrated mainly in the β-turn and in the C-terminal region immediately following the second β-strand. These structural positions suggest that the substitutions in LINGO-3 and LINGO-4 may influence the overall stability or dynamics of the β-hairpin, whereas those in LINGO-2 are more likely to affect peripheral interactions or surface properties without significantly altering the core structural scaffold.

Comparative analyses of the electrostatic surface potentials (Figure S13) demonstrate significant differences among the four peptides, particularly around mutated regions. LINGO-2[1-20] exhibits regions of increased negative potential compared to LINGO-1, while LINGO-3[1-20] and LINGO-4[1-20] display more pronounced positive potential regions, suggesting variations in their interaction properties and specificity.

The hydrophobicity surface potentials (Figure S14) further reveal distinctive surface characteristics across the peptides. LINGO-2[1-20] and LINGO-4[1-20] possess larger hydrophobic patches compared to LINGO-1[1-20], potentially affecting their binding interactions and solubility. Conversely, LINGO-3[1-20] shows a higher degree of hydrophilicity in regions proximal to the β-turn, suggesting altered dynamics or interaction capabilities in aqueous environments.

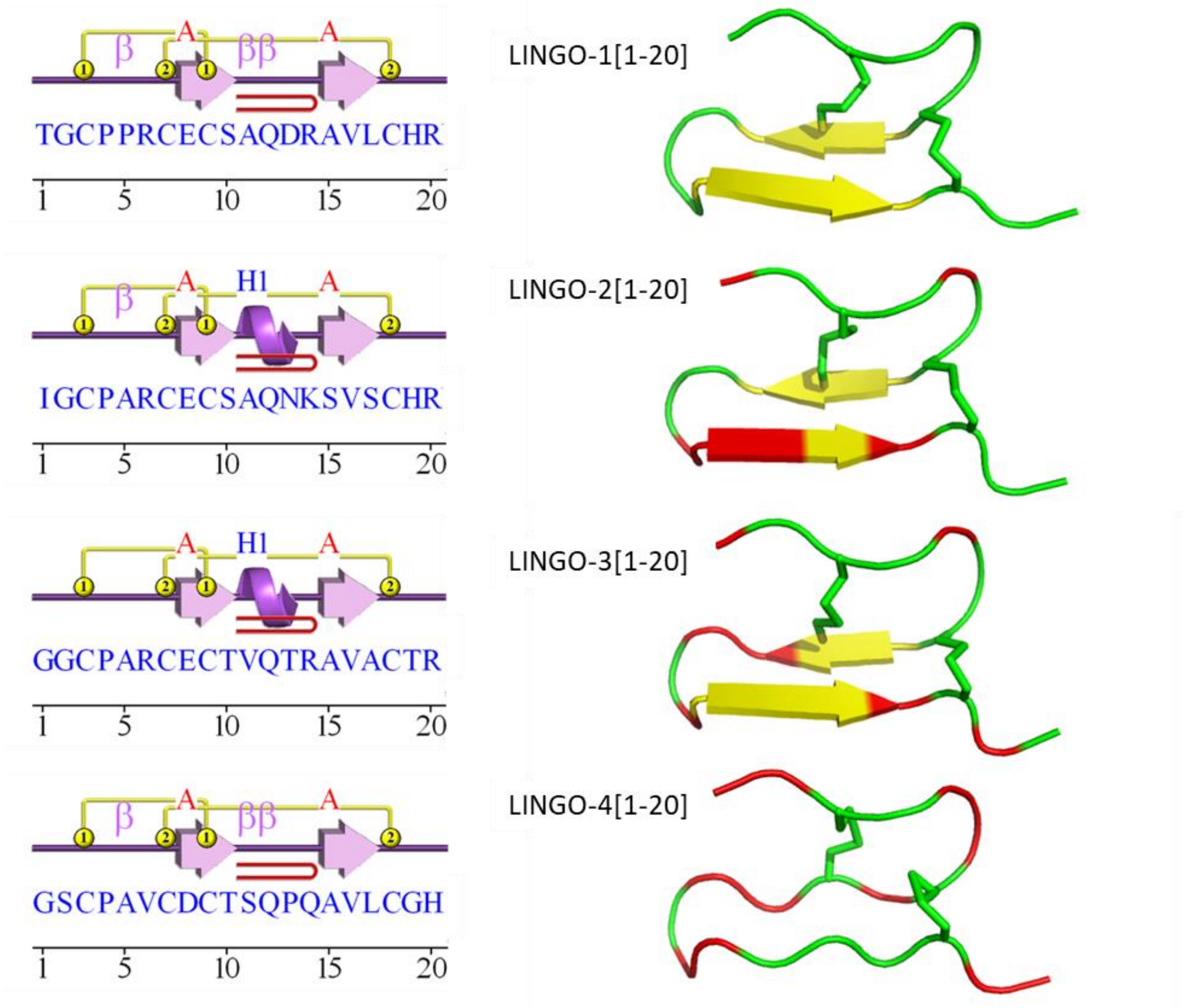

Figure S12. Structural representations of secondary structures and corresponding three-dimensional folds of LINGO-1[1-20], LINGO-2[1-20], LINGO-3[1-20] and LINGO-4[1-20] . Left/ Secondary structure elements. β-strands, α-helices, and disulfide bonds are represented as arrows, cylinders and yellow lines, respectively. β-turns are indicated by pink β and the presence of a β-hairpin is indicated in red. Right/ Cartoon representation of the models. β-strands are shown as yellow arrows. Mutated residues compared to LINGO-1[1-20] are colored in red.

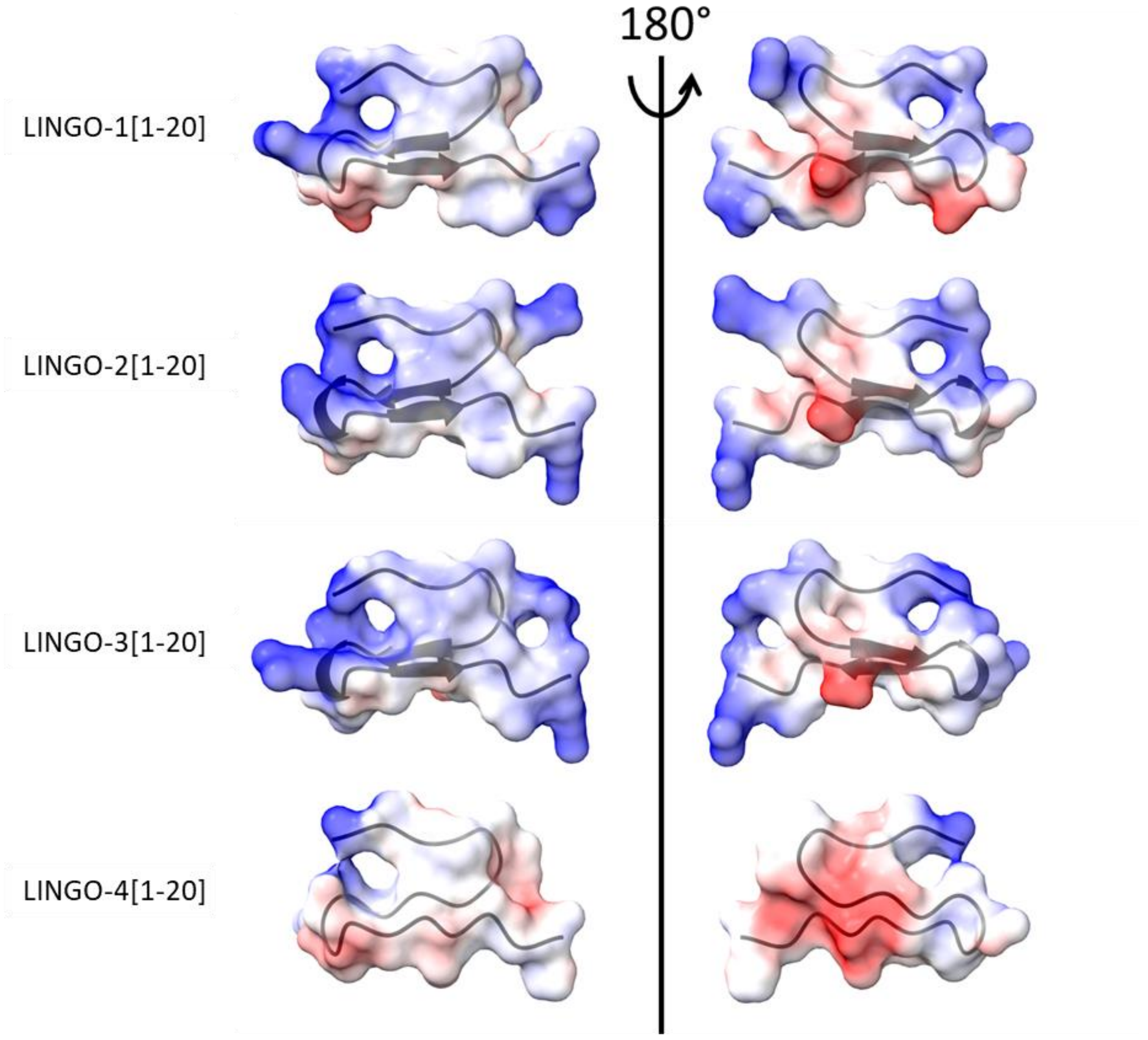

Figure S13. Comparison of electrostatic surface potential for LINGO-1[1-20], LINGO-2[1-20], LINGO-3[1-20] and LINGO-4[1-20], with blue indicating positively charged regions and red indicating negatively charged regions (range –6,6). The surface transparency was set to 50%, allowing the underlying cartoon representation (black) to be seen.

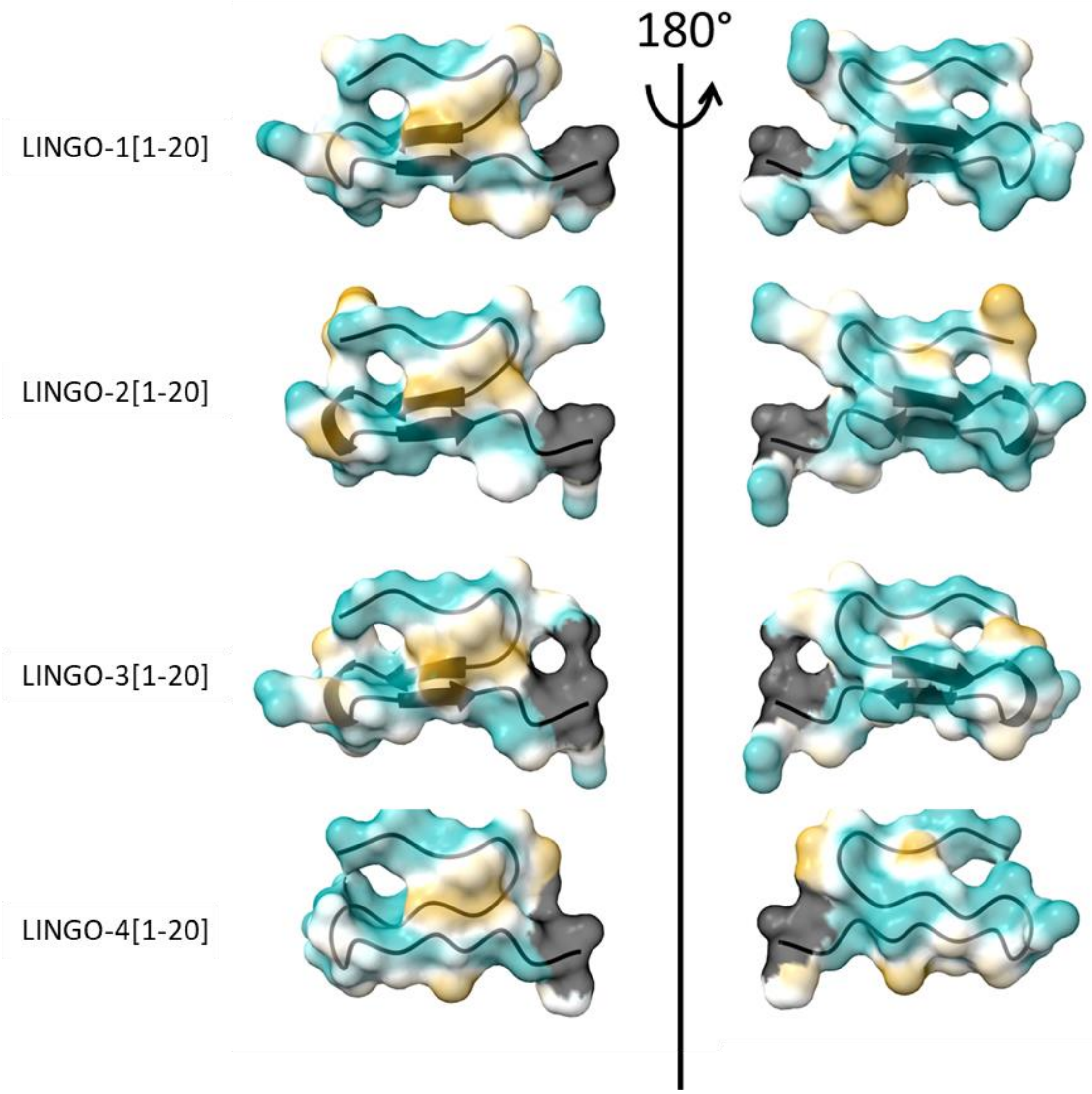

Figure S14. Comparison of hydrophobicity surface potential for LINGO-1[1-20], LINGO-2[1-20], LINGO-3[1-20] and LINGO-4[1-20], where hydrophobic residues are shown in gold and hydrophilic residues in cyan (range -12,12). The surface transparency was set to 50%, allowing the underlying cartoon representation (black) to be seen. Black surface is the C-terminal amide.

5- Synthesis and purification of azido peptide LINGO-1[1-20]GK(N_3) (4)

5-1 SPPS and cleavage of precursor 2

Sequence: H-TG**C(Acm)**PPRCE**C(Acm)**SAQDRAVLCHRGK(N_3)-NH_2 (**2**)

The peptide was synthesized using protocol PS1. ^{3}C(Acm) and ^{9}C(Acm) were introduced using Fmoc-Cys(Acm)-OH as building block. K(N_3) was introduced using Fmoc-Lys(N_3)-OH as building block (3 equiv.), with 2.95 equiv. of HATU and 6 equiv. of DIPEA for 3 h. Cleavage was performed following protocol PS2.

Elongation yield: 84%. Determined by the ratio between the quantity of fluorenylpiperidine released during initial Fmoc deprotection and the quantity released during the Fmoc deprotection of the C-terminal Thr residue (UV titration at 301 nm, ε = 7800 $L{\cdot}mol^{-1}{\cdot}cm^{-1}$).

ESI-MS: [M] average mass calculated for $C_{100}H_{168}N_{40}O_{31}S_4$: 2554.9, found: 2554.2 (deconvoluted).

HPLC purification: The crude peptide was used without any further purification.

HPLC analysis: t_R = 3.92 min. (Chromolith, gradient: 5-50% Solv. B over 7 min.).

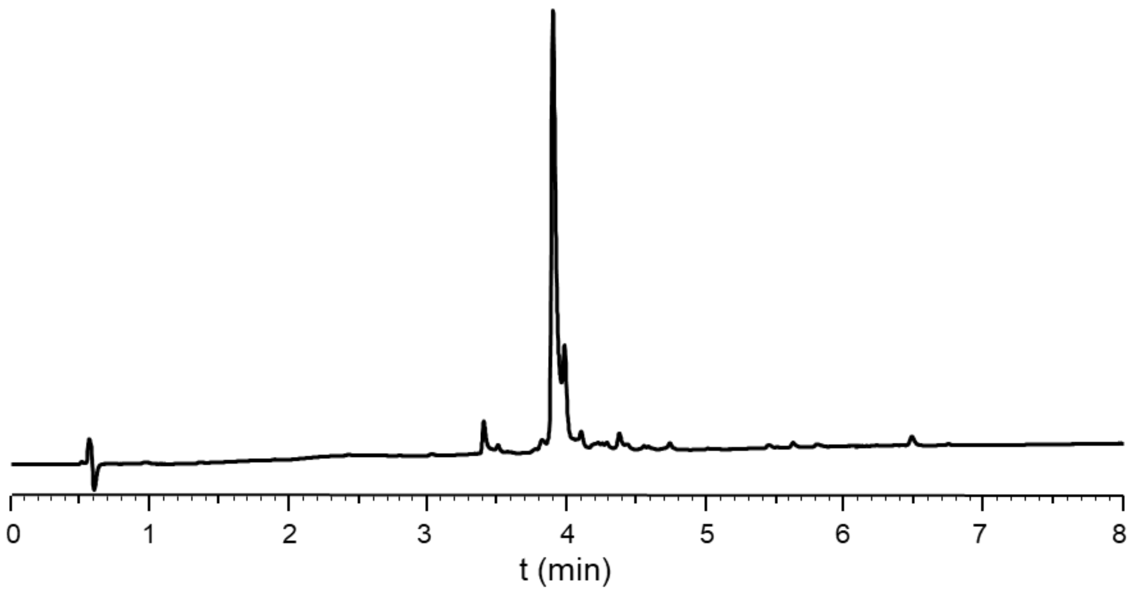

Figure S15. HPLC trace of crude compound **2** (λ = 214 nm).

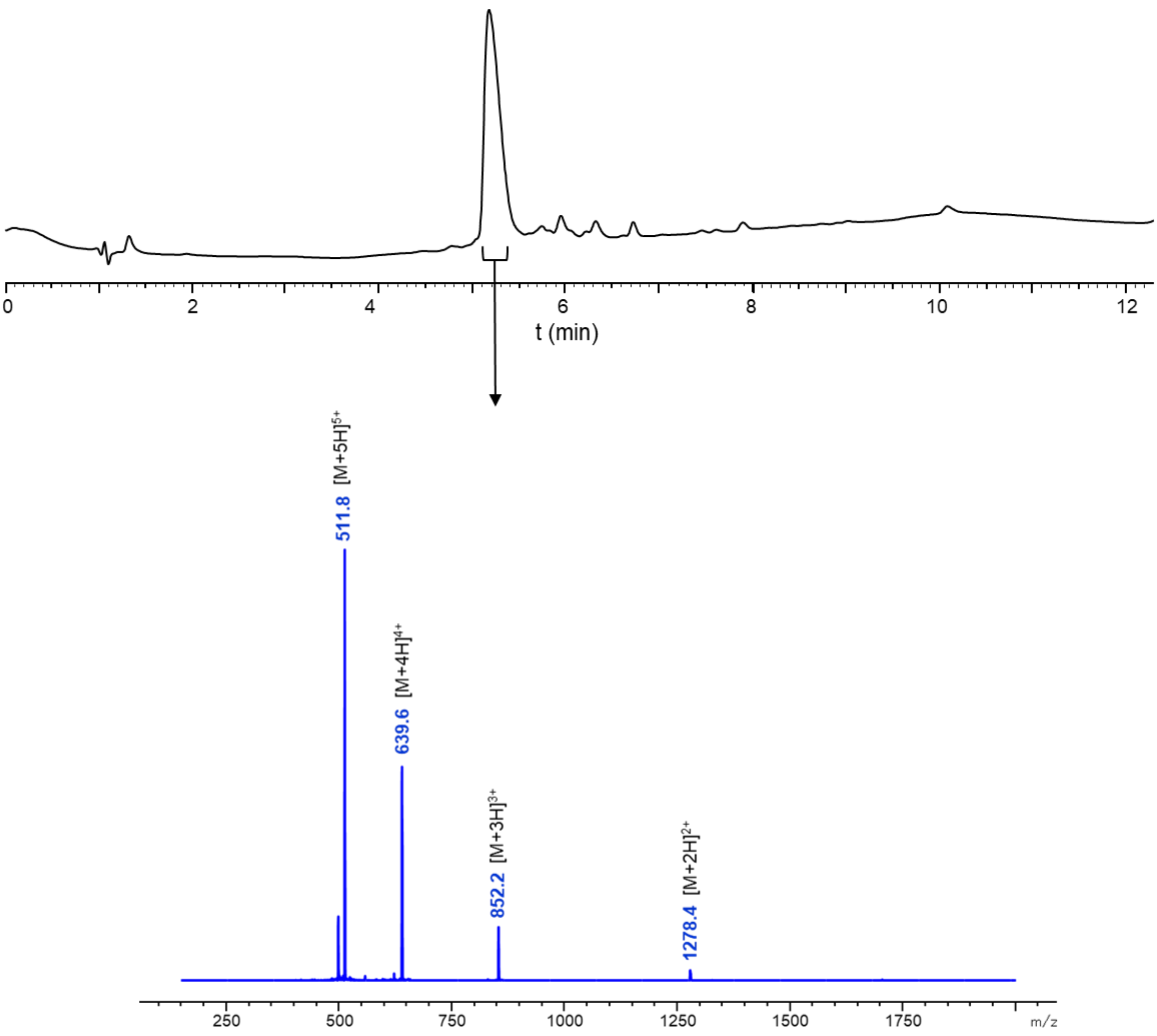

Figure S16. LC-MS analysis of crude compound **2**: A) HPLC trace (λ = 214 nm) and B) ESI-MS spectrum corresponding to the time window indicated as a black arrow (sum of spectra, 5.22-5.56 min).

5-2 Formation of the first SS bridge

Sequence: H-TG**C(Acm)**PPRCE**C(Acm)**SAQDRAVLCHRGK(N_3)-NH_2 (**3**)

To a stirred solution of crude LINGO-1[1-20] peptide **2** (96.4 mg, 30.8 µmol) in 86.4 mL of H_2O, 9.6 mL of DMSO were added dropwise (0.31 mM final peptide concentration). The reaction was stirred at room temperature and monitored by HPLC; after 100 hours, the mixture was diluted with 100 mL water and freeze-dried. Dilution with 100 mL water then freeze-drying was repeated four times to completely remove DMSO and obtain a white fluffy powder.

ESI-MS: [M] average mass calculated for $C_{100}H_{166}N_{40}O_{31}S_4$: 2552.9, found: 2552.4 (deconvoluted).

HPLC purification: The crude peptide was used without any further purification.

HPLC analysis: t_R = 3.19 min. (Chromolith, gradient: 5-50% Solv. B over 5 min.).

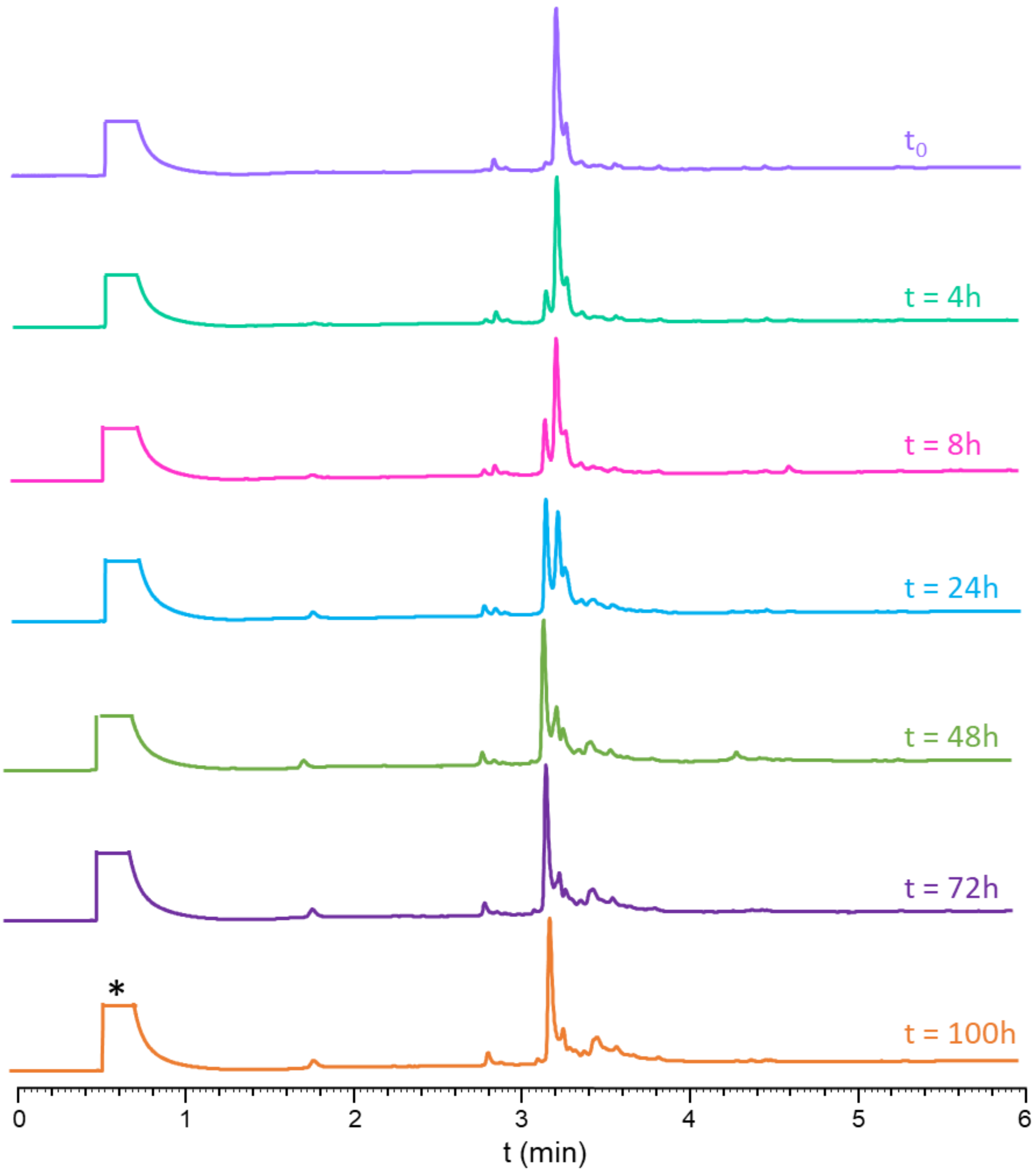

*DMSO

Figure S17. HPLC traces of time-course monitoring of the formation of the first disulfide bridge yielding compound **3** (λ = 214 nm).

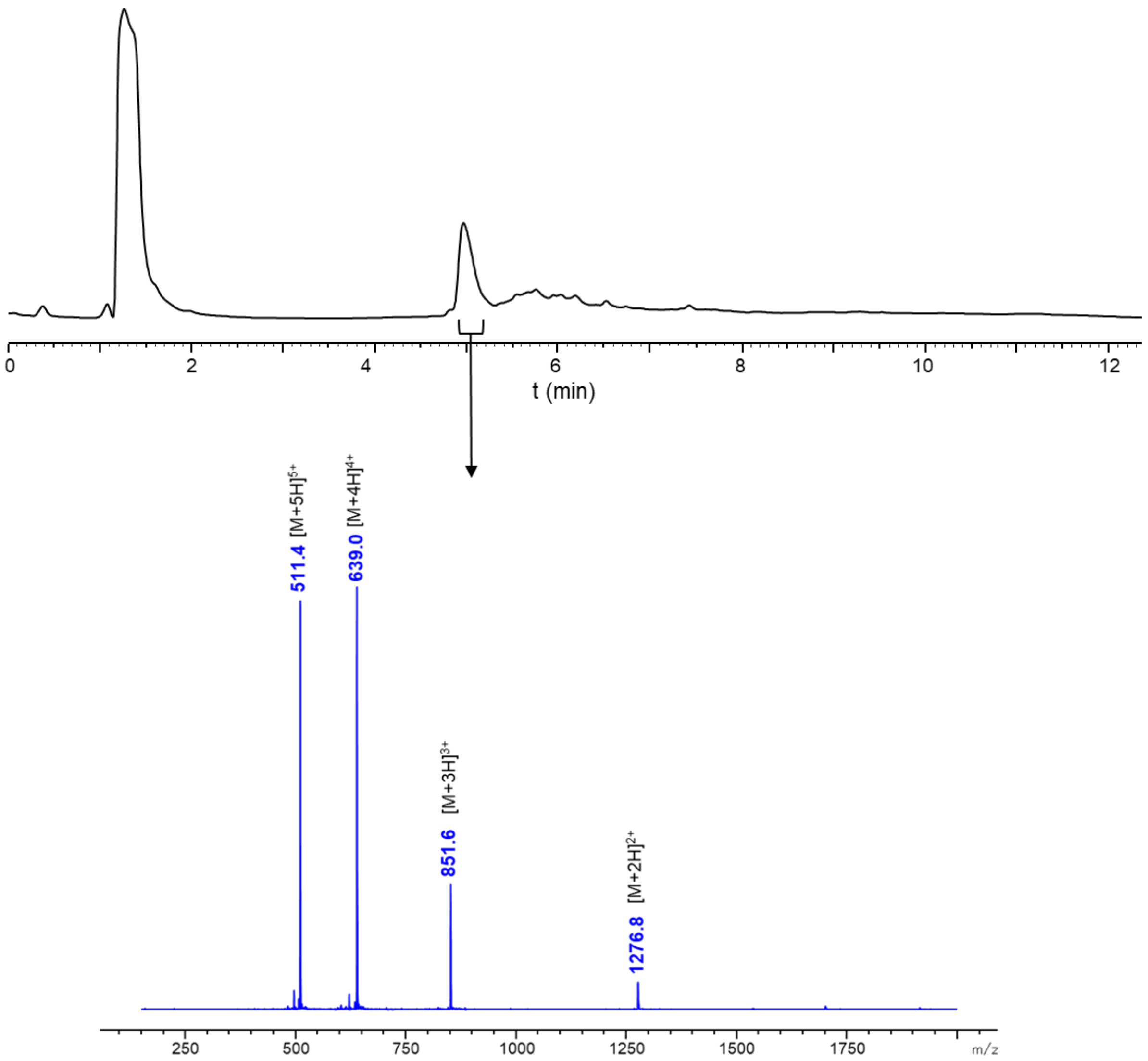

Figure S18. LC-MS analysis of crude compound **3**: A) HPLC trace (λ = 214 nm) and B) ESI-MS spectrum corresponding to the time window indicated as a black arrow (sum of spectra, 5.02-5.26 min).

5-3 Formation of the second SS bridge

Sequence: H-TG**C**PPR**C**E**C**SAQDRAVL**C**HRGK(N_3)-NH_2 (**4**)

Crude peptide **2** (3.9 µmol) was solubilized in glacial AcOH (2.1 mL). To this solution, 1.6 mL of a 25 mM solution of iodine in AcOH, and 0.25 mL of 160 mM aqueous HCl were added, yielding a final peptide concentration of 1 mM. After 2 hours, the reaction was quenched by adding ice-cooled ether (9 volume equivalents) and kept 15 min. at -80 °C to maximize precipitation. The resulting suspension was centrifuged at 4300 rpm for 5 min., the supernatant decanted off, and the resulting pellet crushed and washed with diethyl ether (2 x 50 mL). After careful decantation of the ether, the yellowish solid was analyzed by LC-MS, confirming complete conversion. The crude product was solubilized in 1.8 mL of a water/MeCN/TFA 8:2:0.1 mixture then subjected to direct purification.

ESI-MS: [M] average mass calculated for $C_{94}H_{154}N_{38}O_{29}S_4$: 2408.7, found: 2408.2 (deconvoluted).

HPLC purification: Nucleosil C18, gradient: 10-25% Solv. B over 40 min.; 20% overall yield based on initial resin loading.

HPLC analysis: t_R = 3.18 min. (Chromolith, gradient: 5-50% Solv. B over 5 min.).

A)

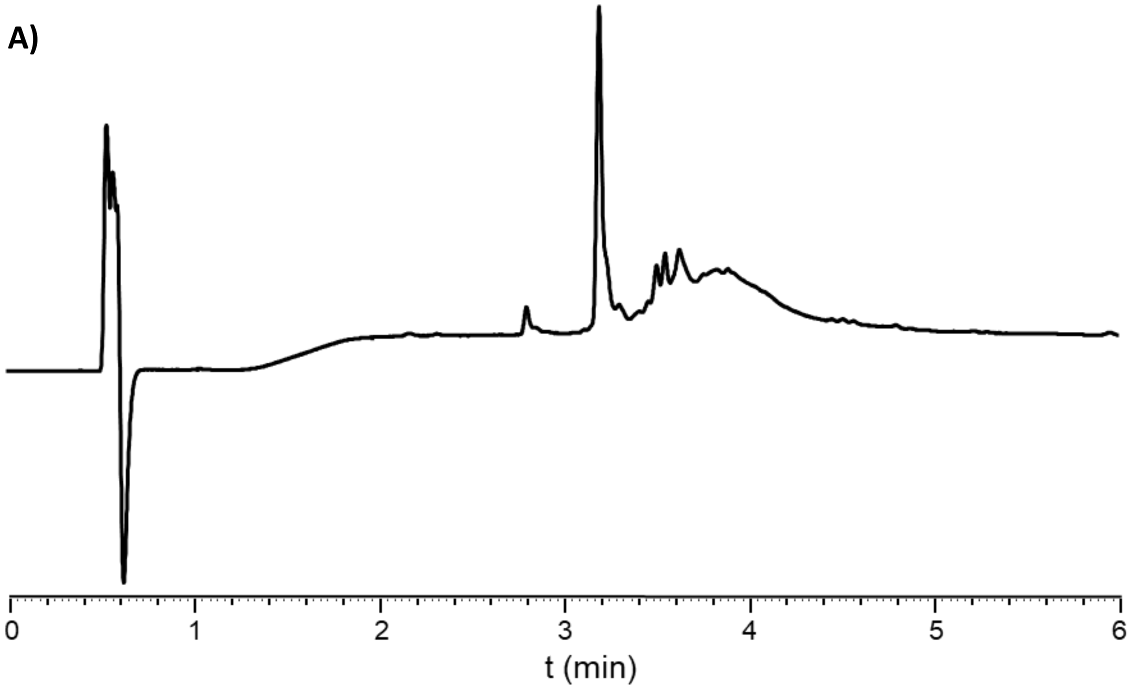

B)

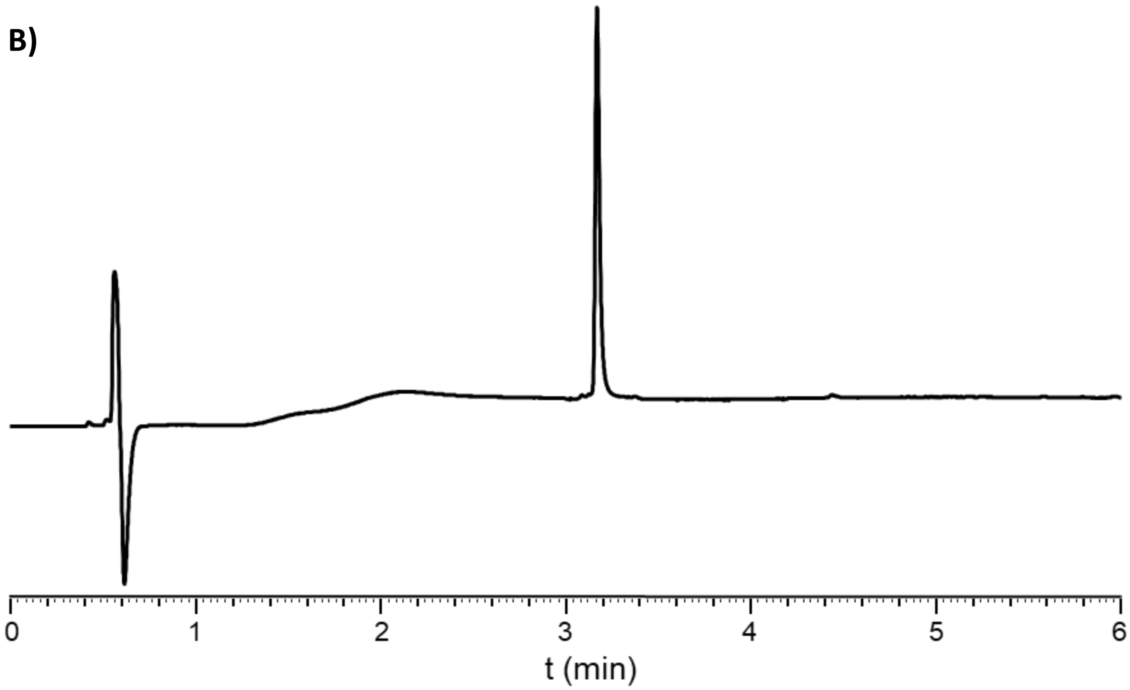

C)

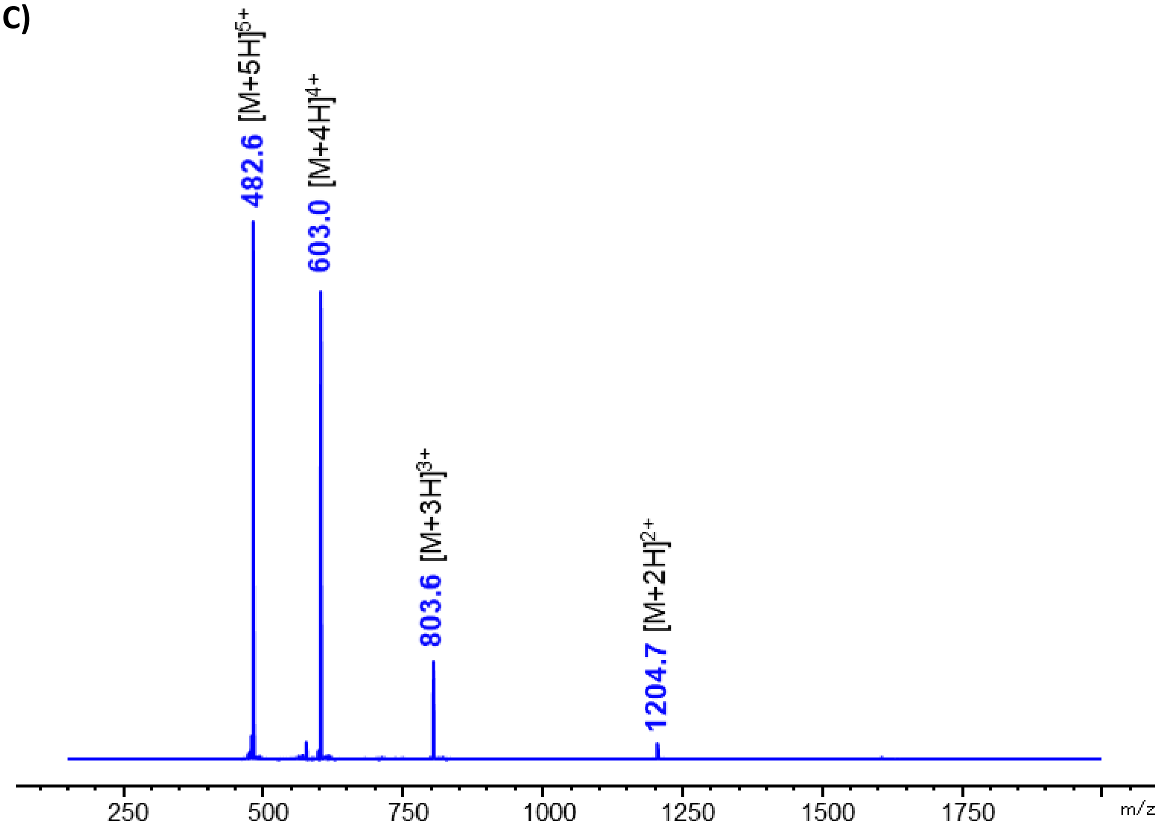

Figure S19. HPLC traces of (A) crude and (B) purified compound **4** (λ = 214 nm). (C) ESI-MS spectrum of purified compound **4**.

Optimization of I_2-mediated disulfide formation

For the transformation of *bis*-Acm peptide **3** into disulfide **4**, a series of experiments were conducted to reduce the total volume of the reaction mixture and, consequently, the amount of diethyl ether required for precipitation. In this aim, we varied the peptide concentration and the equivalents of iodine used, while keeping in line with recommendations of Wade and coll.[11] to maximize precipitation efficiency (5 to maximum 10% water in the aquesous AcOH reaction mixture / minimum six volumes of ether). As shown by HPLC chromatograms (Fig. S16), increasing the peptide concentration did only slightly increase the proportion of by-products (peak B, corresponding to a mixture of dimers and trimers), and enabled to reduce the volume of diethyl ether used for precipitation by nearly 16-fold as compared to the initial conditions optimized by Wade and coll. for insulin-like peptides (conditions of entry 1).[11] Optimal conversion was achieved using a 1 mM solution of peptide with 10 equivalents of iodine at room temperature for 2 hours.

Table S3. Optimization of the disulfide formation induced by iodine. Volumes reported for 1 µmol of peptide **3**.

Entry	[peptide] mM	Equiv. I_2	25 mM I_2/AcOH (mL)	AcOH (mL)	160 mM HCl (mL)	Diethyl ether (mL)
**1**	0.095	70	2.8	7.20	0.56	95
**2**	0.188	30	1.2	3.80	0.33	32
**3**	0.5	20	0.8	1.075	0.125	12
**4**	1	10	0.4	0.5375	0.0625	6

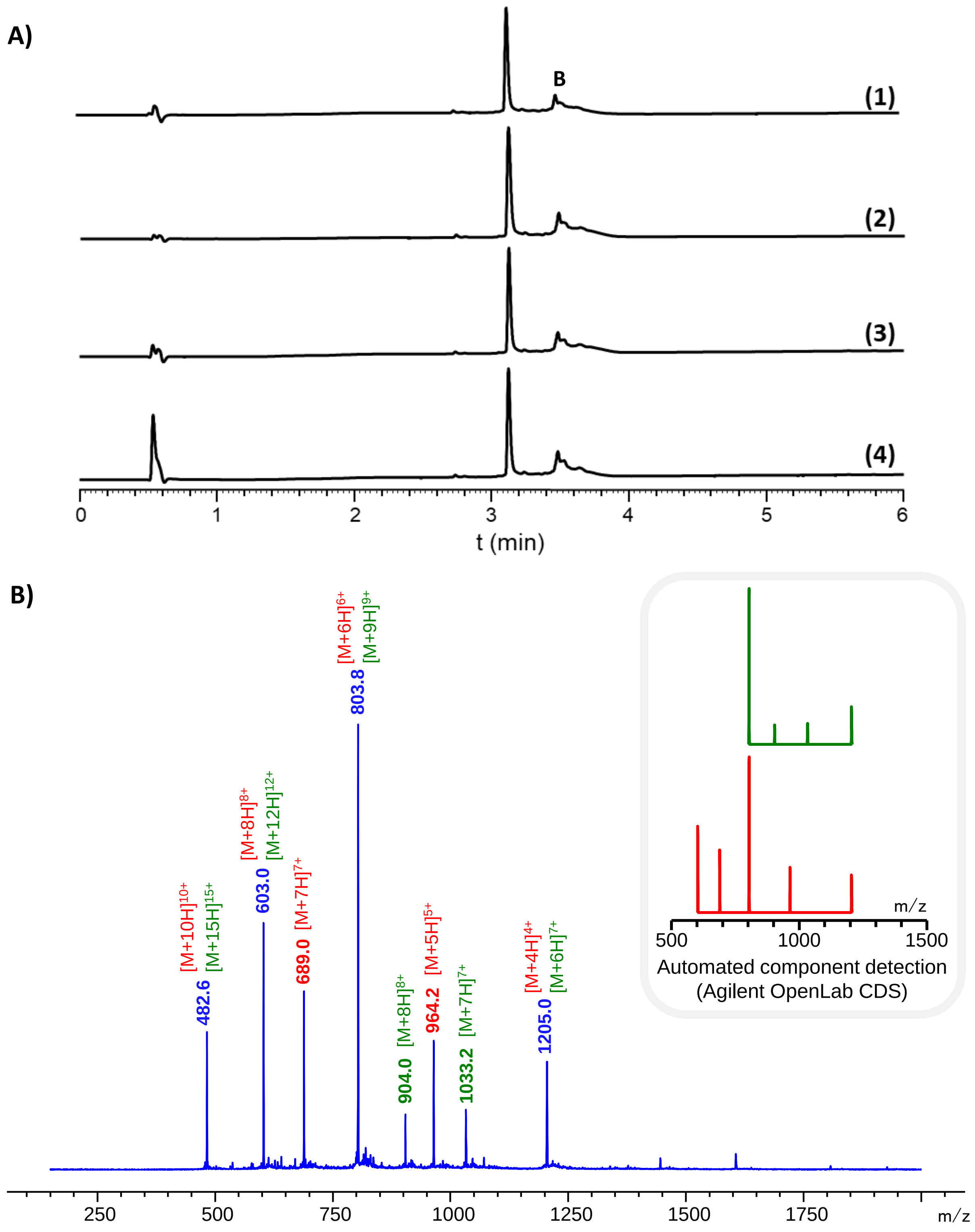

Figure S20: (A) HPLC traces (λ = 214 nm) of crude **4** using conditions described in table S3. (B) Example of an MS spectrum for peak B, containing signals attributed to dimer (red) and trimer (green) forms. Dimer: [M] average mass calculated for $C_{188}H_{308}N_{76}O_{58}S_8$: 4817.5, found: 4816.5 (deconvoluted); trimer: [M] average mass calculated for $C_{282}H_{462}N_{114}O_{87}S_{12}$: 7226.2, found: 7224.8 (deconvoluted).

5-4 Stability under oxidative folding conditions

A model single-domain DRP, the β-defensin AvBD103b was synthesized as previously described.[12]

Sequence: H-SFGLCRLRRGFCARGRCRFPSIPIGRCSRFVQCCRRVW-OH

ESI-MS: [M] average mass calculated for $C_{194}H_{308}N_{70}O_{43}S_6$: 4501.4, found: 4500.7 (deconvoluted).

The disulfide-rich peptide was incubated at room temperature under argon in a typical oxidative folding redox buffer (10 µM final peptide concentration): 100 mM TRIS, 1 mM EDTA, 1 mM glutathione (100 equiv.), 0.1 mM glutathione disulfide (10 equiv.), final pH = 8.5, deoxygenated through vacuum/argon cycles. The reaction mixture was analyzed over time through RP-HPLC (Chromolith, gradient: 20-50% over 5 minutes for LINGO1 peptide **4**, 10-40 over 5 minutes for AvBD103b).

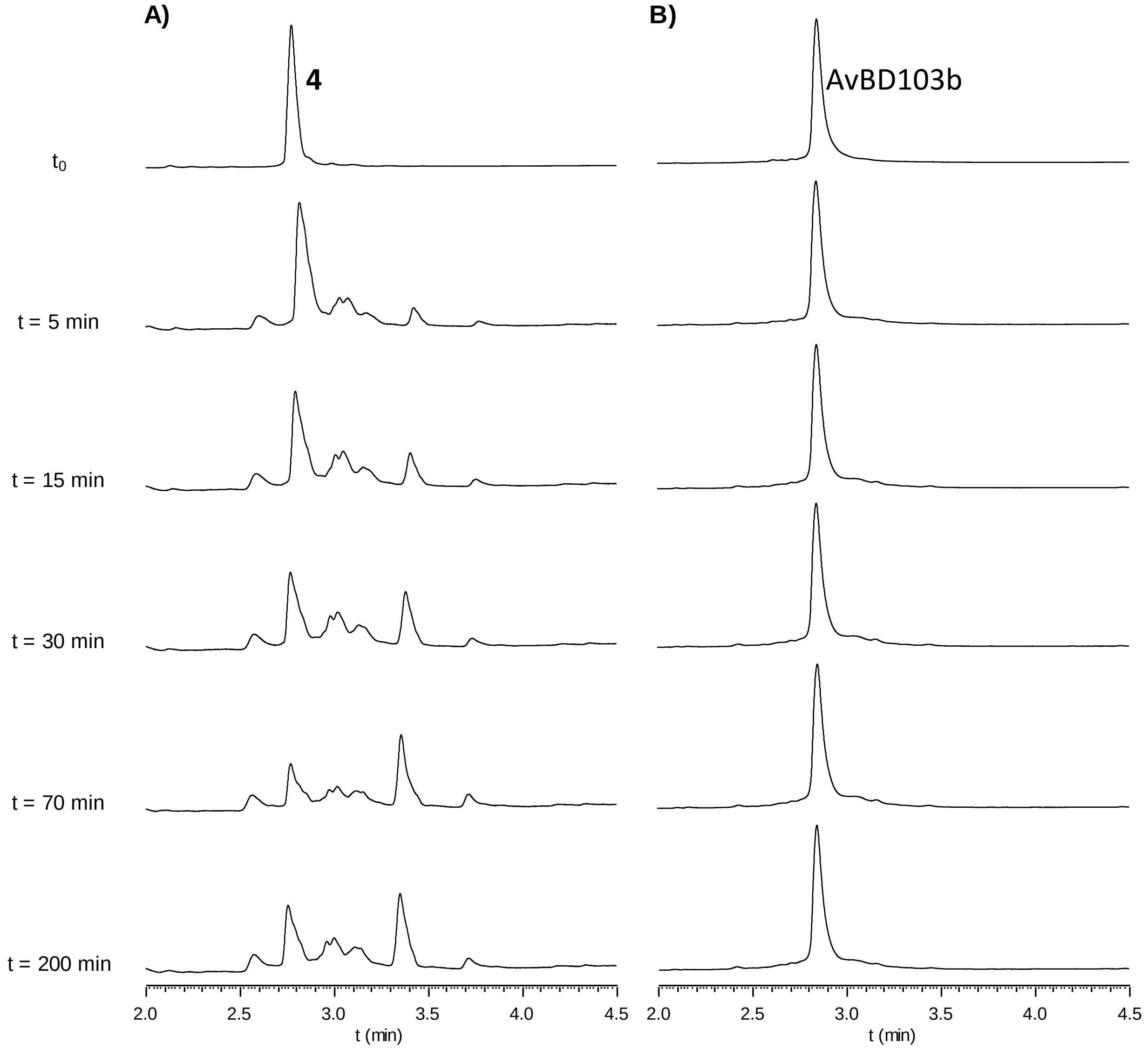

Figure S21: HPLC traces (λ = 214 nm) of (A) LINGO1 peptide **4**, and (B) AvBD103b[12] at different timepoints during incubation in oxidative folding redox buffer.

6- Synthesis of peptide spacers 5a-5e

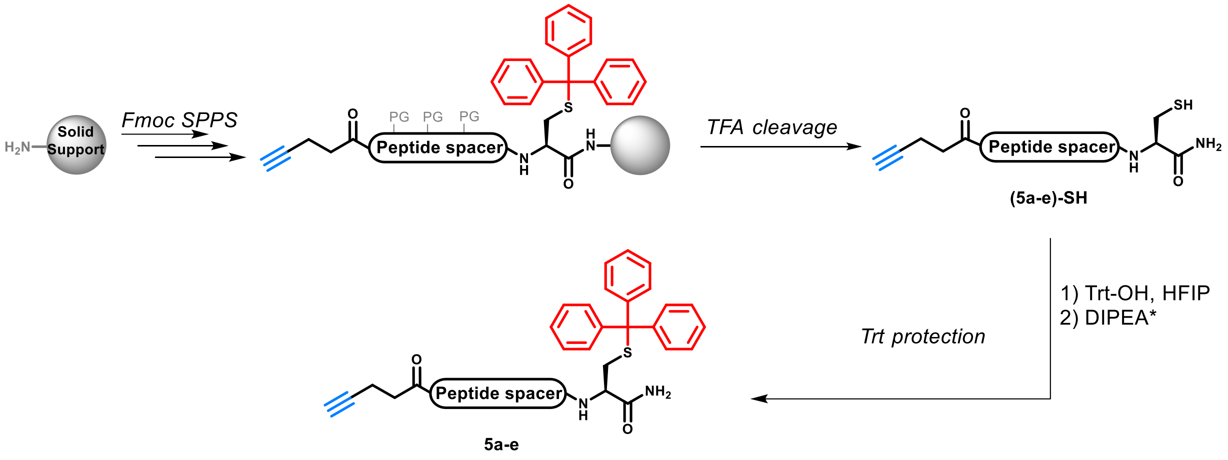

Peptide spacer sequences: a = $(GGGGS)_2GG$ / b = $(GGGGS)_4GGGG$ / c = $(GGGGS)_8G$ / d = P_6 / e = P_{12}

Scheme S2. General scheme for the synthesis of peptide spacers **5a**-**5e**. *Step required for **5a-5c** only. PG: protecting group (*e.g.* serine *t*Bu ethers for peptidyl resins **S3a-S3c**).

6-1 Synthesis of peptide spacer 5a

- **SPPS and cleavage**

Sequence: Pent-GGGGSGGGGSGGC-NH_2 (**5a-SH**)

The peptide was synthesized using protocol PS1. Cleavage was performed following protocol PS2.

Elongation yield: 69%. Determined by the ratio between the quantity of fluorenylpiperidine released during final Fmoc deprotection and the quantity released during the Fmoc deprotection of the C-terminal Cys residue (UV titration at 301 nm, ε = 7800 L·mol^{-1}·cm^{-1}).

ESI-MS: $[M+H]^+$ monoisotopic mass calculated for $C_{34}H_{53}N_{14}O_{16}S_1$: 945.4, found: 945.2.

HPLC purification: The crude peptide was used without any further purification.

HPLC analysis: t_R = 2.08 min. (Chromolith, gradient: 3-70% Solv. B over 7 min.).

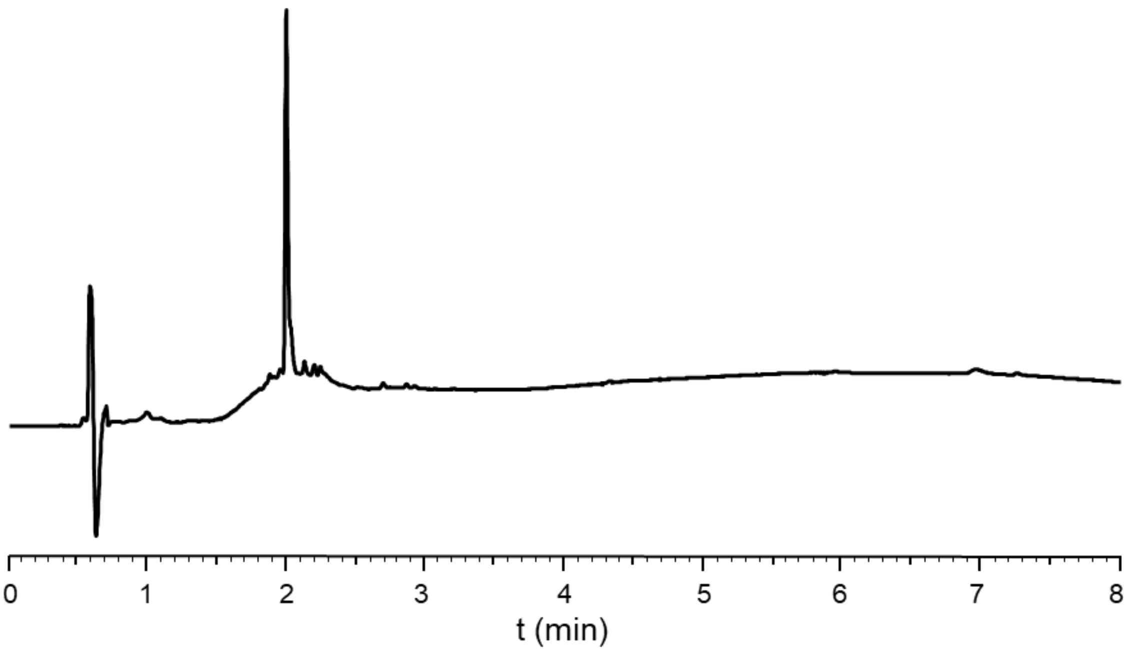

Figure S22. HPLC trace of crude compound **5a-SH** (λ = 214 nm).

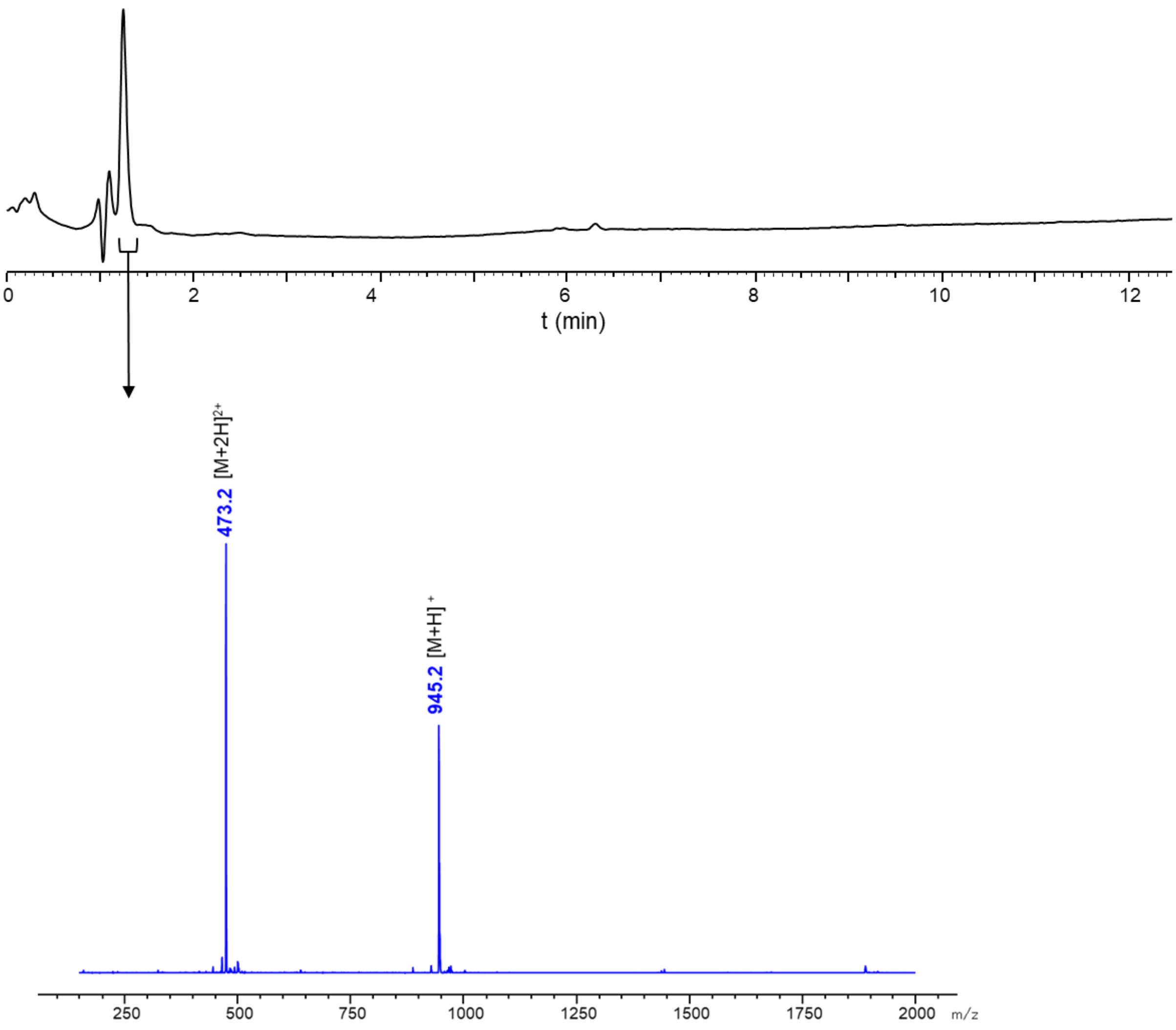

Figure S23. LC-MS analysis of crude compound **5a-SH**: A) HPLC trace (λ = 214 nm) and B) ESI-MS spectrum corresponding to the time window indicated as a black arrow (sum of spectra, 1.41-1.53 min).

- **Trityl protection**

Sequence: Pent-GGGGSGGGGSGGC(Trt)-NH_2 (**5a**)

Cysteine residue tritylation was performed using protocol PS3.

ESI-MS: $[M\text{-}Trt+2H]^+$ monoisotopic mass calculated for $C_{34}H_{52}N_{14}O_{16}S_1$: 945.4; found: 945.2 (corresponding to loss of trityl group during ionization. A mass corresponding to the trityl carbocation, calculated: 243.1, found: 243.2, is also observed).

HPLC purification: Chromolith, gradient: 25-40% B over 8 min.; 22% yield.

HPLC analysis: t_R = 4.92 min. (Chromolith, gradient: 3-70% Solv. B over 7 min.).

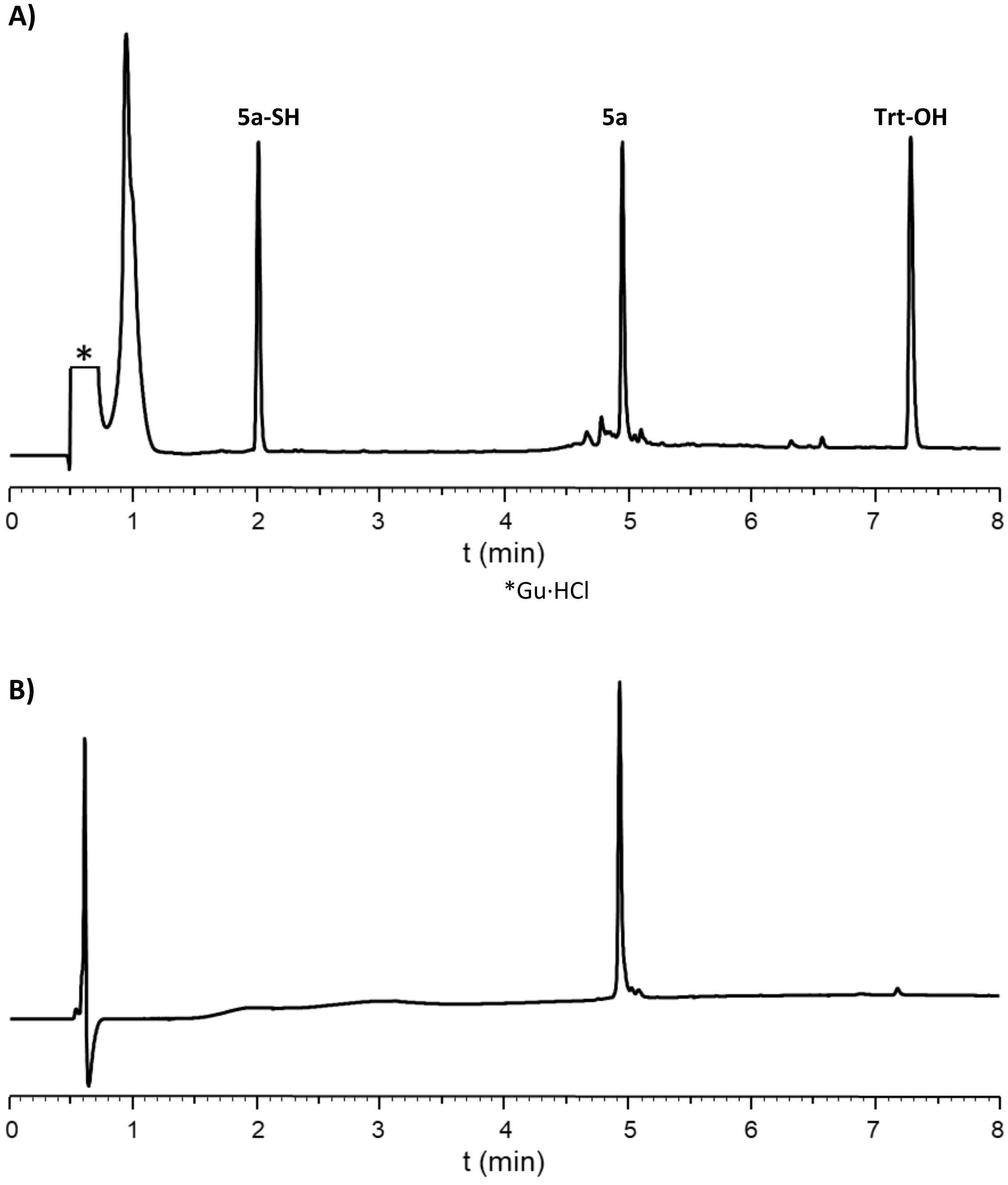
A)
5a-SH
5a
Trt-OH
*
0 1 2 3 4 5 6 7 8
t (min)
*Gu·HCl
B)
0 1 2 3 4 5 6 7 8
t (min)

C)

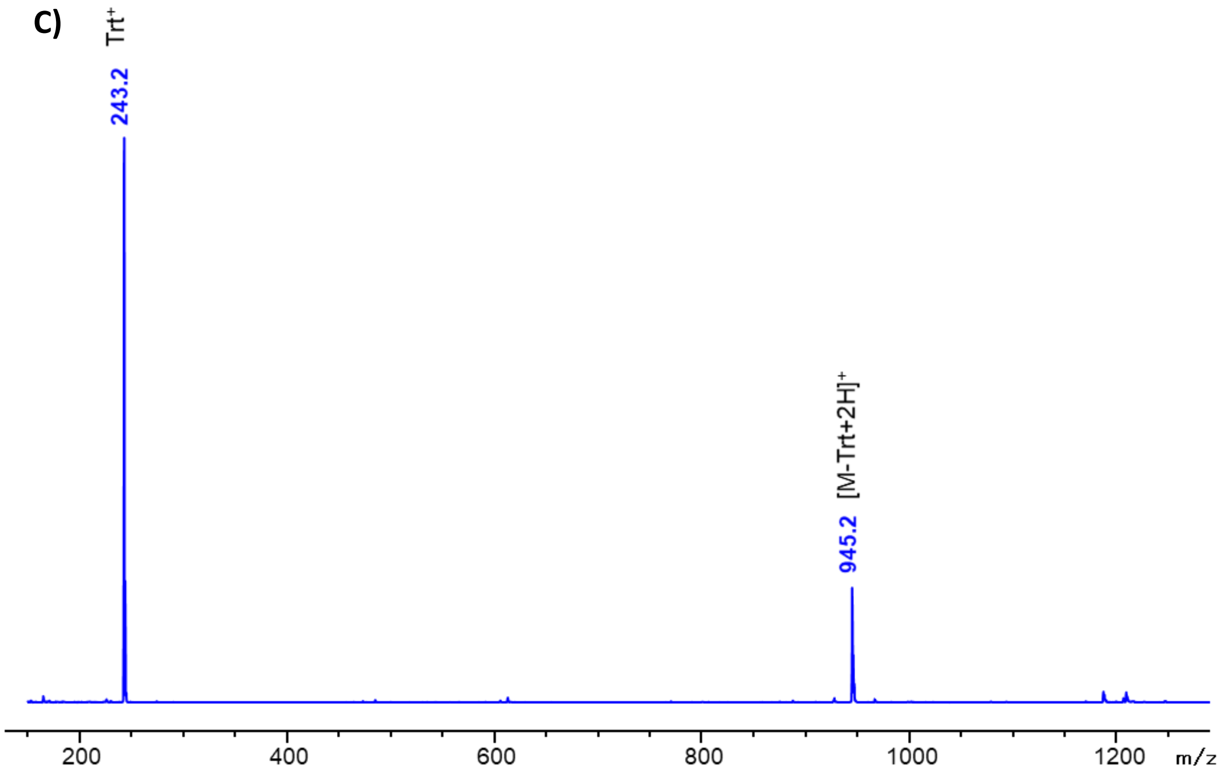

Figure S24. HPLC traces of (A) crude and (B) purified compound **5a** (λ = 214 nm). (C) ESI-MS spectrum of purified compound **5a.**

6-2 Synthesis of peptide spacer 5b

- **SPPS and cleavage**

Sequence: Pent-GGGGSGGGGSGGGGSGGGGSGGGGC-NH_2 (**5b-SH**)

The peptide was synthesized using protocol PS1. Cleavage was performed following protocol PS2.

Elongation yield: 97%. Determined by the ratio between the quantity of fluorenylpiperidine released during initial Fmoc deprotection and the quantity released during the Fmoc deprotection of the C-terminal Ala residue (UV titration at 301 nm, ε = 7800 $L{\cdot}mol^{-1}{\cdot}cm^{-1}$).

ESI-MS: $[M+H]^+$ monoisotopic mass calculated. for $C_{60}H_{93}N_{26}O_{30}S_1$: 1689.7, found: 1689.8.

HPLC purification: The crude peptide was used without any further purification.

HPLC analysis: t_R = 2.09 min. (Chromolith, gradient: 3-70% Solv. B over 7 min.).

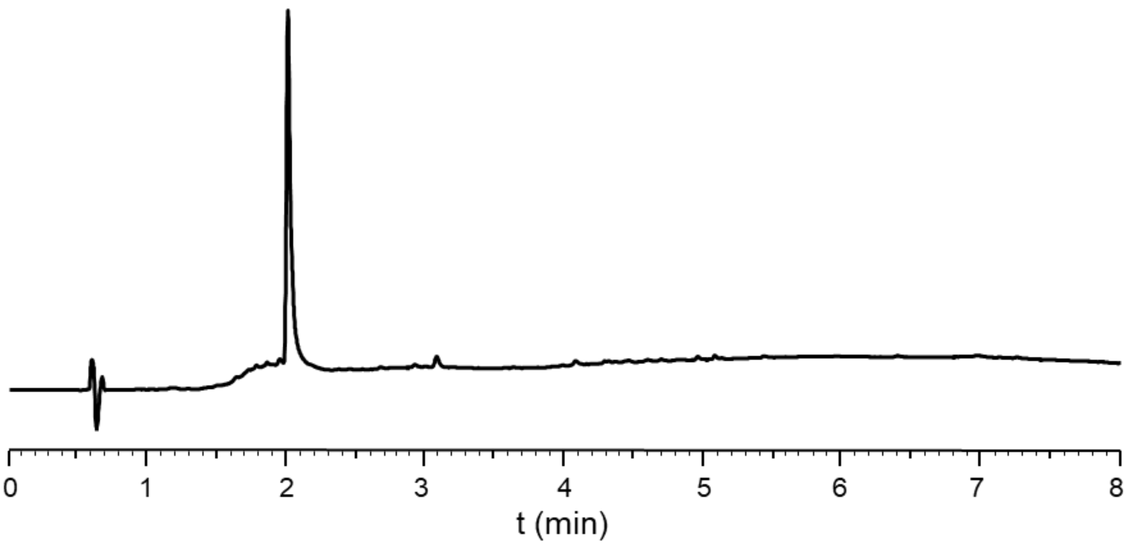

Figure S25. HPLC trace of crude compound **5b-SH** (λ = 214 nm).

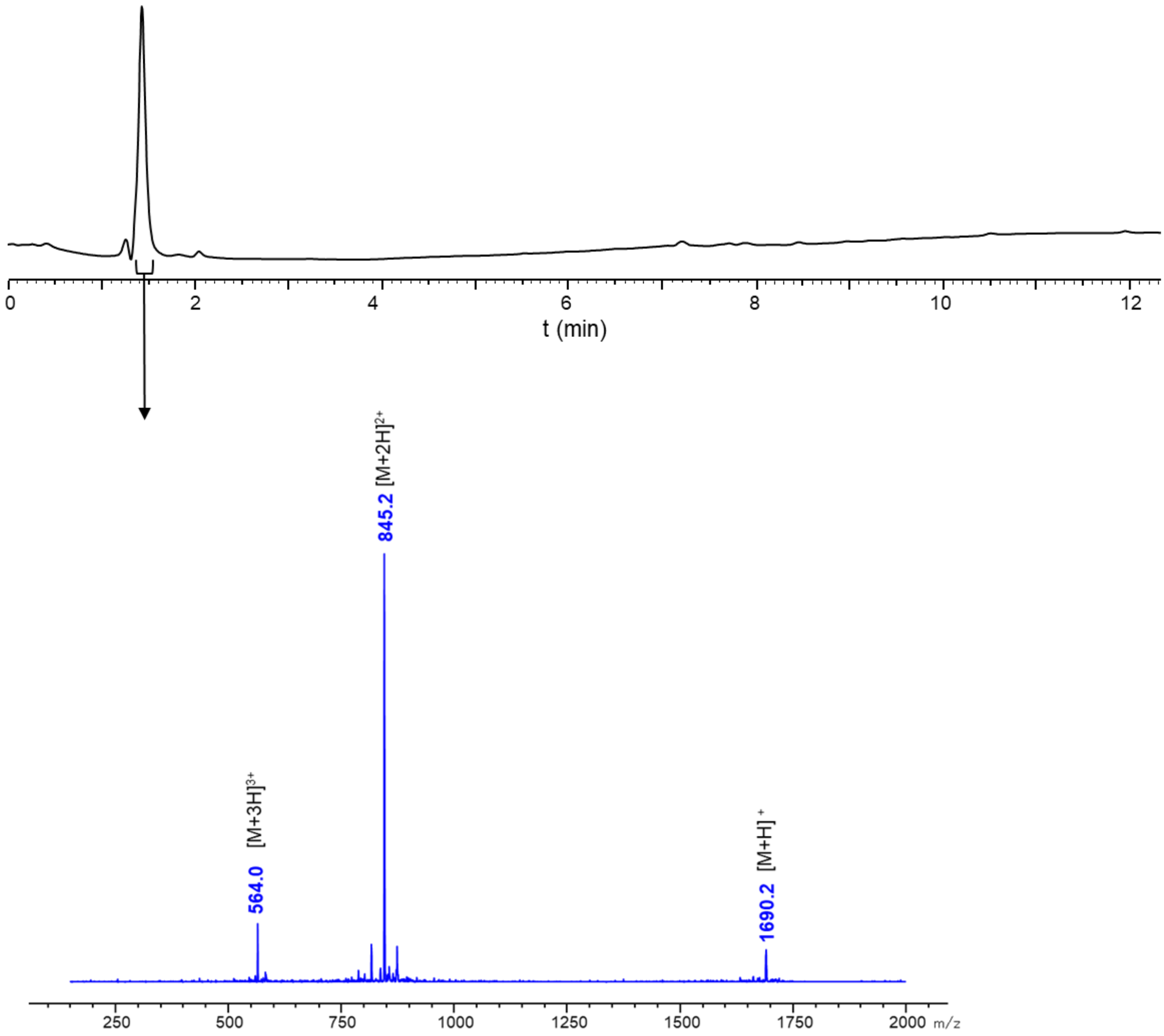

Figure S26. LC-MS analysis of crude compound **5b-SH**: A) HPLC trace (λ = 214 nm) and B) ESI-MS spectrum corresponding to the time window indicated as a black arrow (sum of spectra, 1.51-1.64 min).

- **Trityl protection**

Sequence: Pent-GGGGSGGGGSGGGGSGGGGSGGGGC(Trt)-NH_2 (**5b**)

Cysteine residue tritylation was performed using protocol PS3.

ESI-MS: $[M\text{-}Trt+3H]^{2+}$ monoisotopic mass calculated for $C_{60}H_{94}N_{26}O_{30}S_1$: 845.3, found: 845.2 (corresponding to loss of trityl group during ionization. A mass corresponding to the trityl carbocation, calculated: 243.1, found: 243.2, is also observed).

HPLC purification: Chromolith, gradient: 15-40% B over 10 min.; 34% yield.

HPLC analysis: t_R = 4.68 min. (Chromolith, gradient: 3-70% Solv. B over 7 min.).

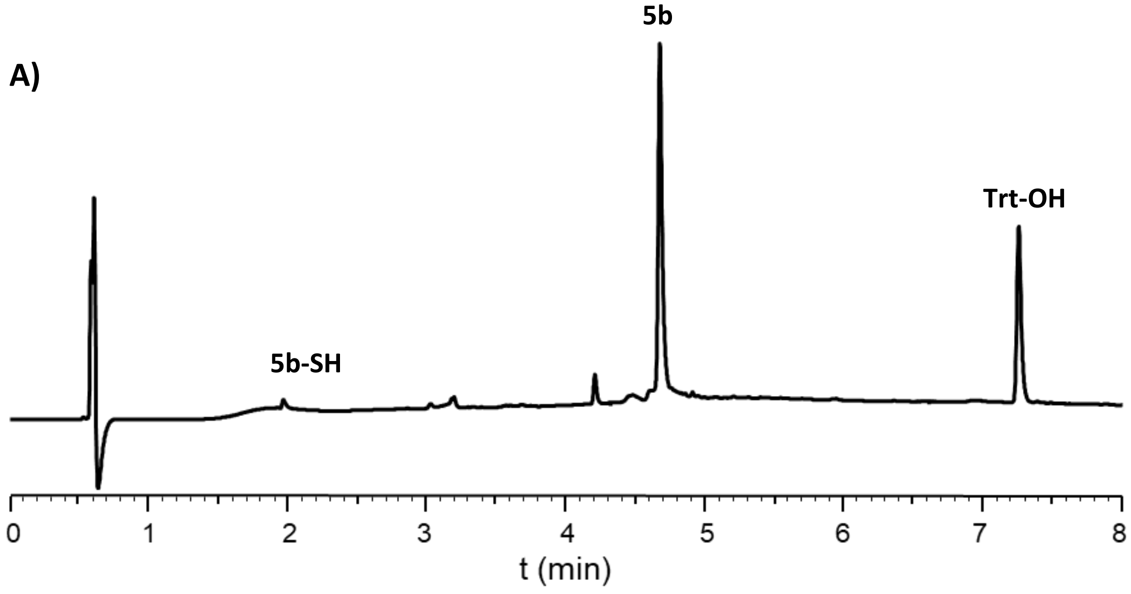

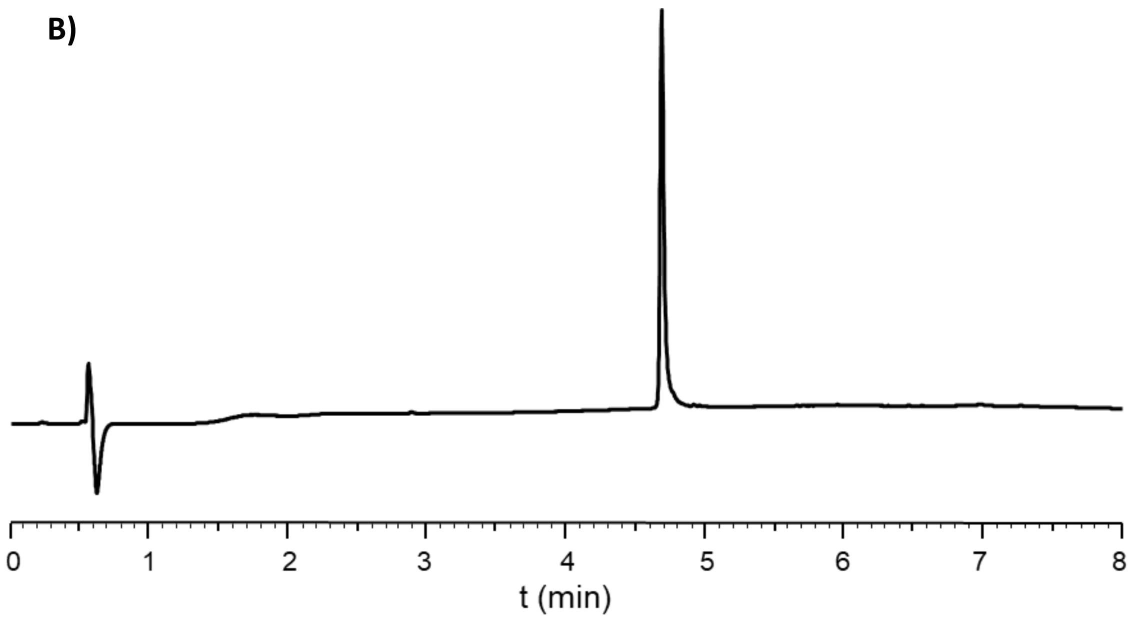

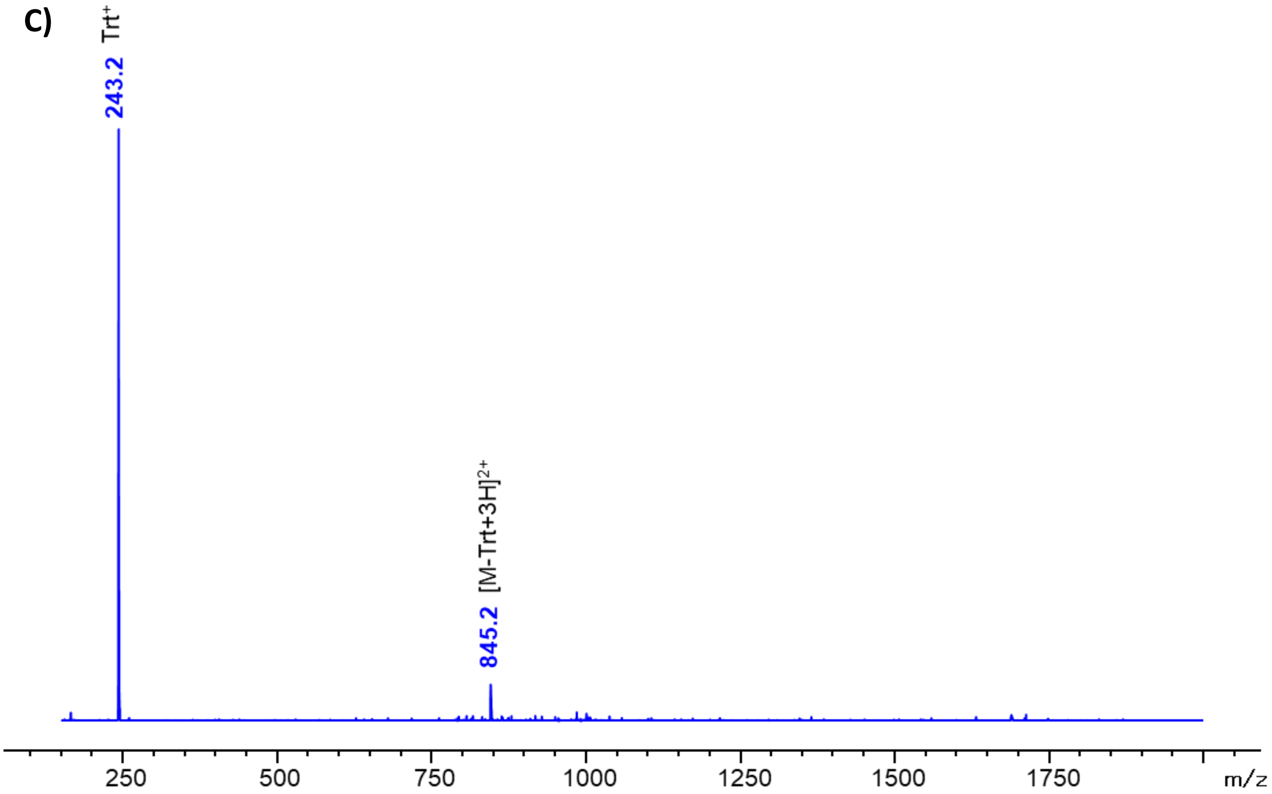

Figure S27. HPLC traces of (A) crude and (B) purified compound **5b** (λ = 214 nm). (C) ESI-MS spectrum of purified compound **5b.**

6-3 Synthesis of peptide spacer 5c

- **SPPS and cleavage**

Sequence: Pent-GGGGSGGGGSGGGGSGGGGSGGGGSGGGGSGGGGSGGGGSGC-NH_2 (**5c-SH**)

The peptide was synthesized using protocol PS1. Cleavage was performed following protocol PS2.

Elongation yield: 93%. Determined by the ratio between the quantity of fluorenylpiperidine released during initial Fmoc deprotection and the quantity released during the Fmoc deprotection of the C-terminal Ala residue (UV titration at 301 nm, ε = 7800 L·mol^{-1}·cm^{-1}).

ESI-MS: [M] average mass calculated for $C_{98}H_{151}N_{43}O_{51}S_1$: 2779.6, found: 2778.7 (deconvoluted).

HPLC purification: The crude peptide was used without any further purification.

HPLC analysis: t_R = 2.07 min. (Chromolith, gradient: 3-70% Solv. B over 7 min.).

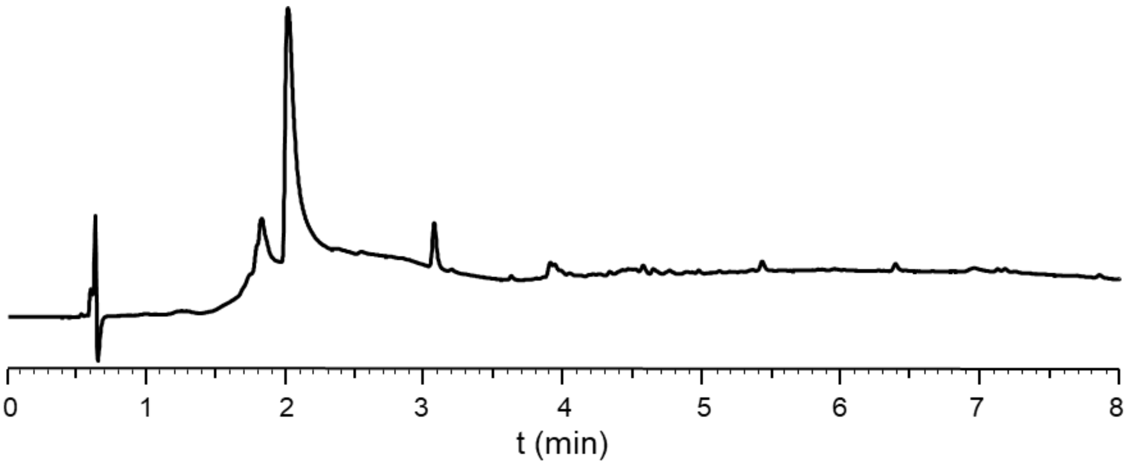

Figure S28. HPLC trace of crude compound **5c-SH** (λ = 214 nm).

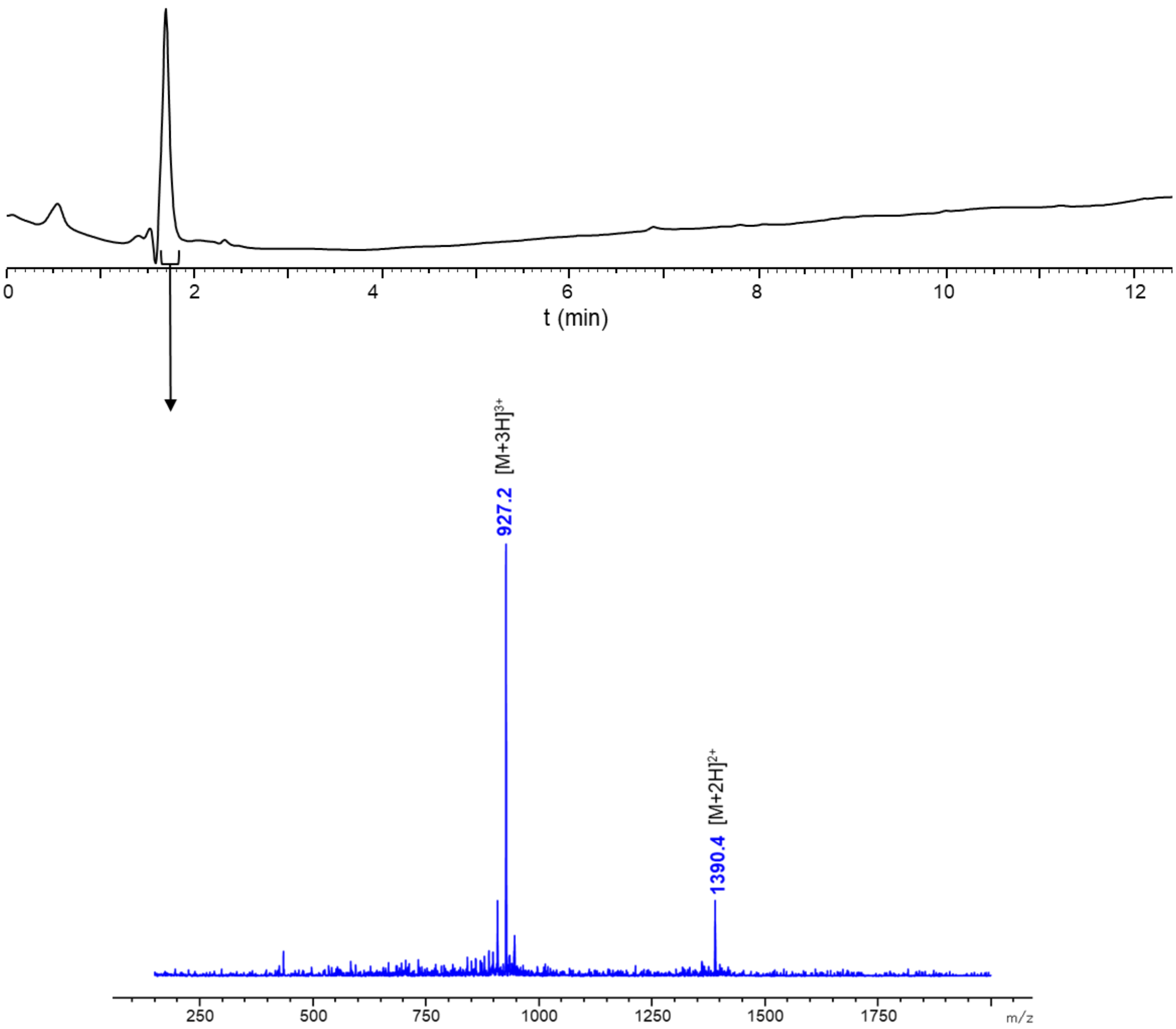

Figure S29. LC-MS analysis of crude compound **5c-SH**: A) HPLC trace (λ = 214 nm) and B) ESI-MS spectrum corresponding to the time window indicated as a black arrow (sum of spectra, 1.71-1.85 min).

- **Trityl protection**

Sequence: Pent-GGGGSGGGGSGGGGSGGGGSGGGGSGGGGSGGGGSGGGGSGC(Trt)-NH_2 (**5c**)

Cysteine residue tritylation was performed using protocol PS3.

ESI-MS: $[M\text{-}Trt+3H]^{2+}$ monoisotopic mass calculated for $C_{98}H_{153}N_{43}O_{51}S_1$: 1390.8, found: 1390.2 (corresponding to loss of trityl group during ionization. A mass corresponding to the trityl carbocation, calculated: 243.1, found: 243.2, is also observed).

HPLC purification: Chromolith, gradient: 10-40% B over 10 min.; 19% yield.

HPLC analysis: t_R = 4.44 min. (Chromolith, gradient: 3-70% Solv. B over 7 min.).

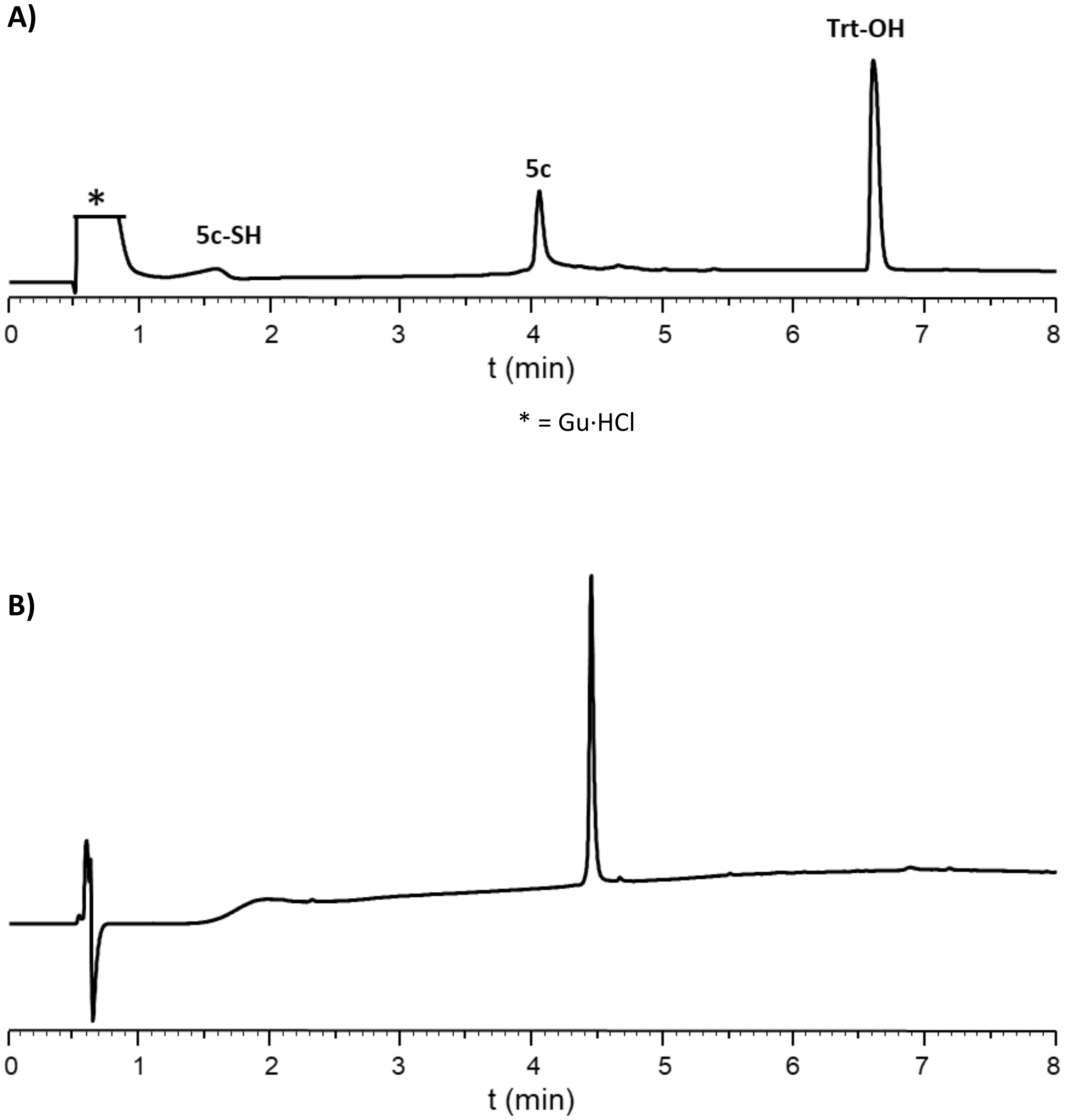
A)
Trt-OH
5c
*
5c-SH
0 1 2 3 4 5 6 7 8
t (min)
* = Gu·HCl
B)
0 1 2 3 4 5 6 7 8
t (min)

C)

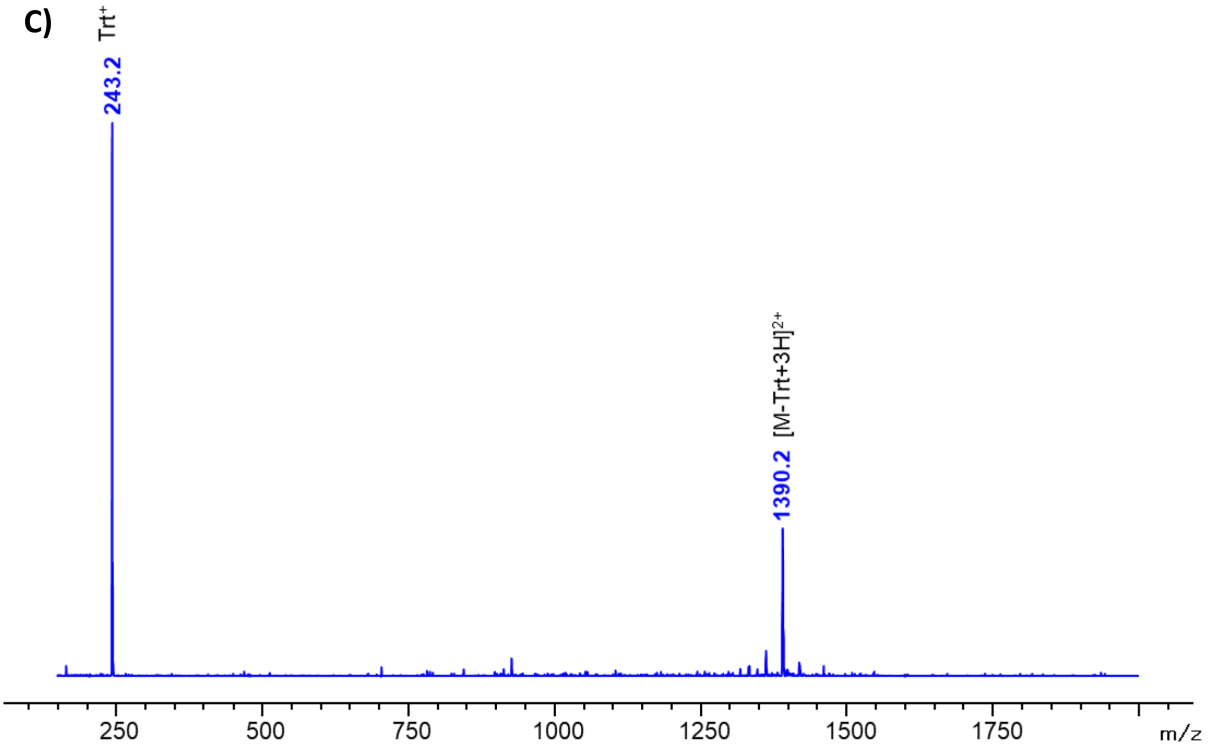

Figure S30. HPLC traces of (A) crude (Chromolith, gradient: 5-80% Solv. B over 7 min.) and (B) purified compound **5c** (λ = 214 nm). (C) ESI-MS spectrum of purified compound **5c.**

6-4 Synthesis of peptide spacer 5d

- **SPPS and cleavage**

Sequence: Pent-PPPPPPC-NH_2 (**5d-SH**)

The peptide was synthesized using protocol PS1. Cleavage was performed following protocol PS2.

Elongation yield: 98%. Determined by the ratio between the quantity of fluorenylpiperidine released during initial Fmoc deprotection and the quantity released during the Fmoc deprotection of the C-terminal Ala residue (UV titration at 301 nm, ε = 7800 L·mol^{-1}·cm^{-1}).

ESI-MS: $[M+H]^+$ monoisotopic mass calculated for $C_{38}H_{55}N_8O_8S$: 783.4, found: 783.4.

HPLC purification: The crude peptide was used without any further purification.

HPLC analysis: t_R = 2.63 min. (Chromolith, gradient: 5-95% Solv. B over 7 min.).

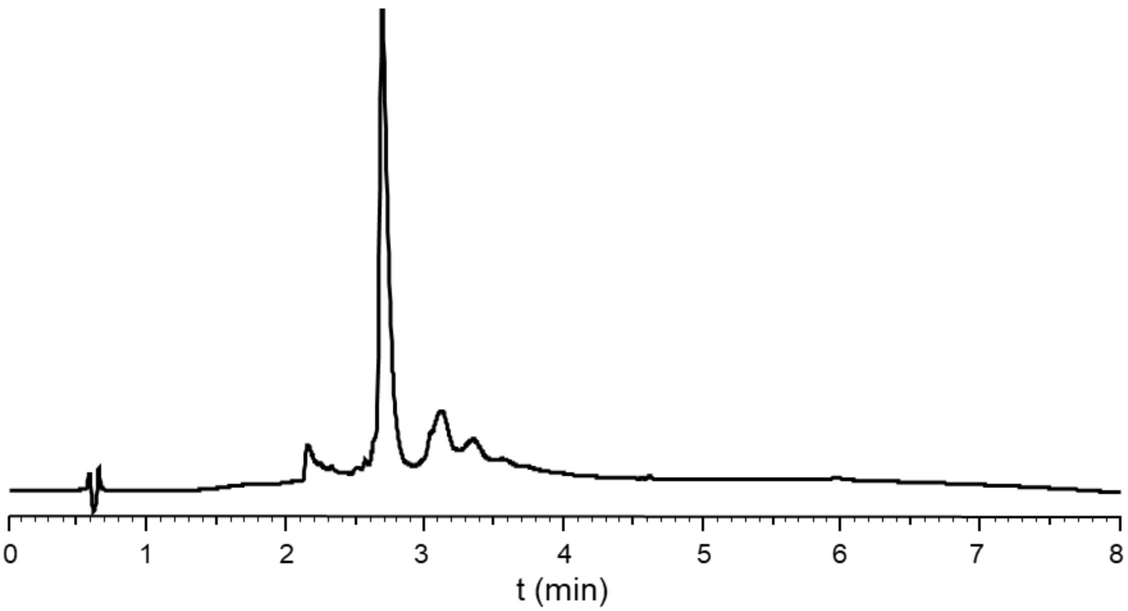

Figure S31. HPLC trace of crude compound **5d-SH** (λ = 214 nm).

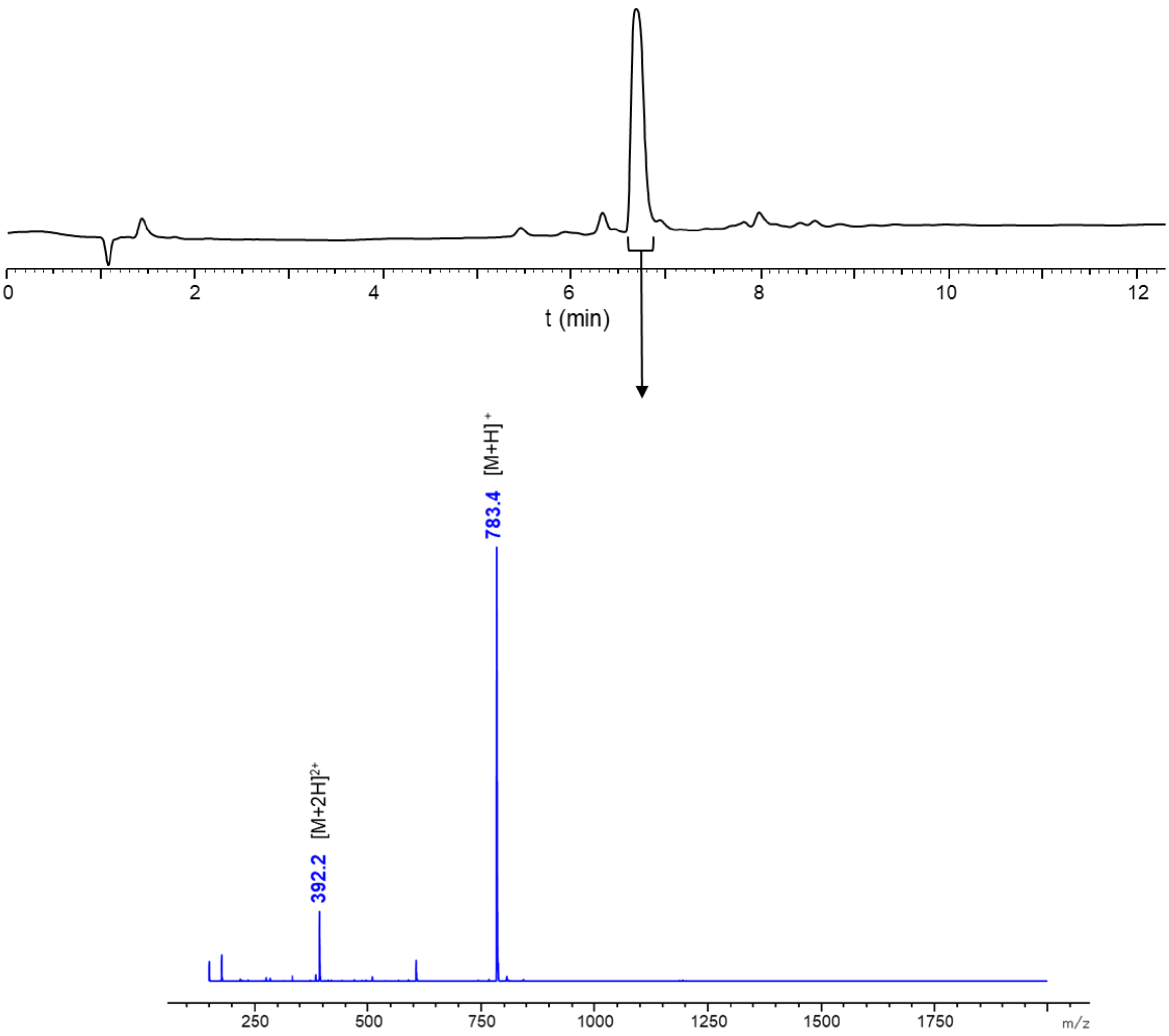

Figure S32. LC-MS analysis of crude compound **5d-SH**: A) HPLC trace (λ = 214 nm) and B) ESI-MS spectrum corresponding to the time window indicated as a black arrow (sum of spectra, 6.74-6.93 min).

- **Trityl protection**

Sequence: Pent-PPPPPPC(Trt)-NH_2 (**5d**)

Cysteine residue tritylation was performed using protocol PS4.

ESI-MS: $[M+H]^+$ monoisotopic mass calculated for $C_{57}H_{68}N_8O_8S_1$: 1025.5, found: 1025.4.

HPLC purification: Nucleosil C18, gradient: 30% Solv. B during 8 min., then 30-60% Solv. B over 60 min.; 26% yield.

HPLC analysis: t_R = 3.08 min. (Chromolith, gradient: 35-55% Solv. B over 5 min.).

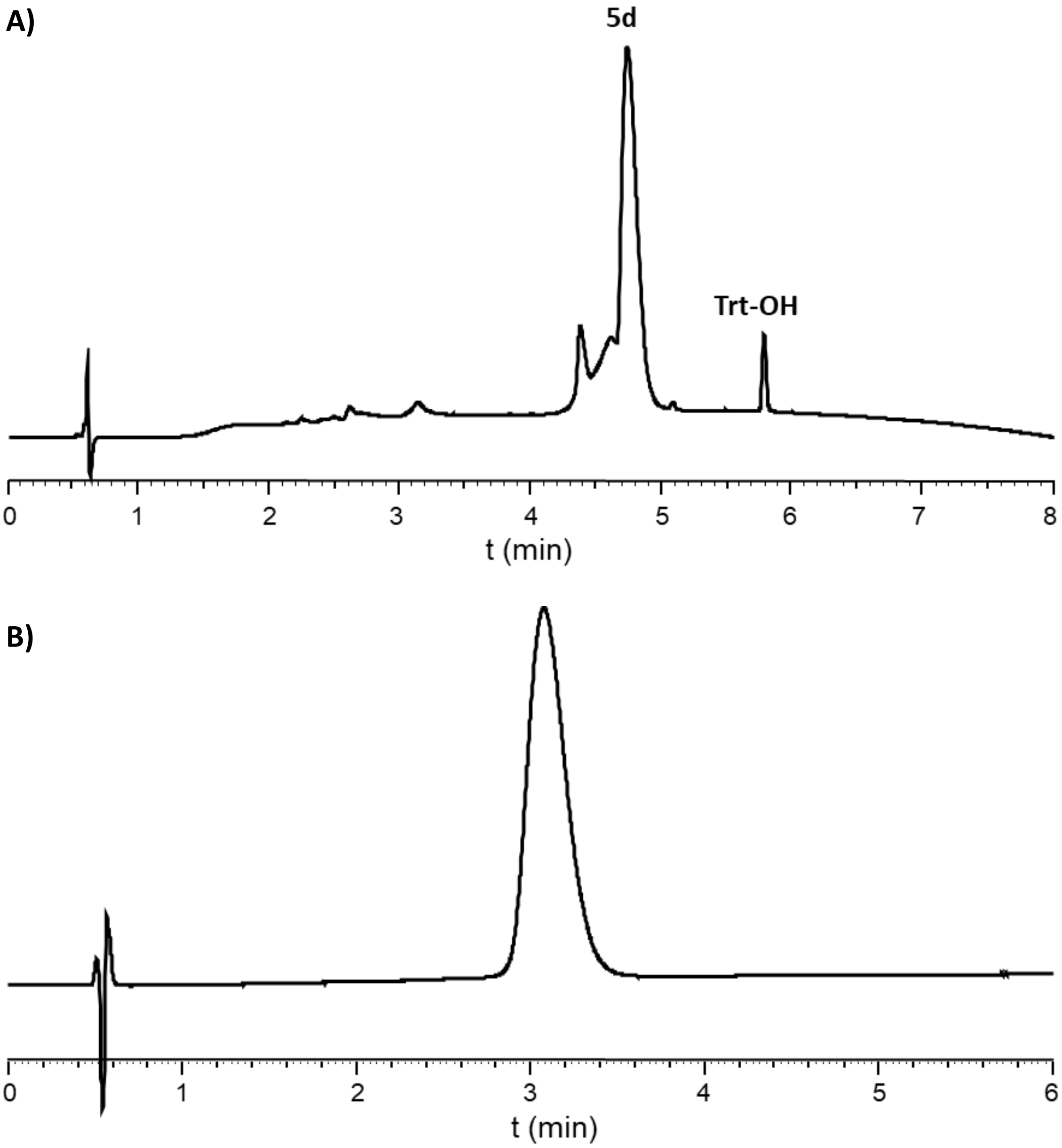
A)
5d
Trt-OH
0 1 2 3 4 5 6 7 8
t (min)
B)
0 1 2 3 4 5 6
t (min)

C)

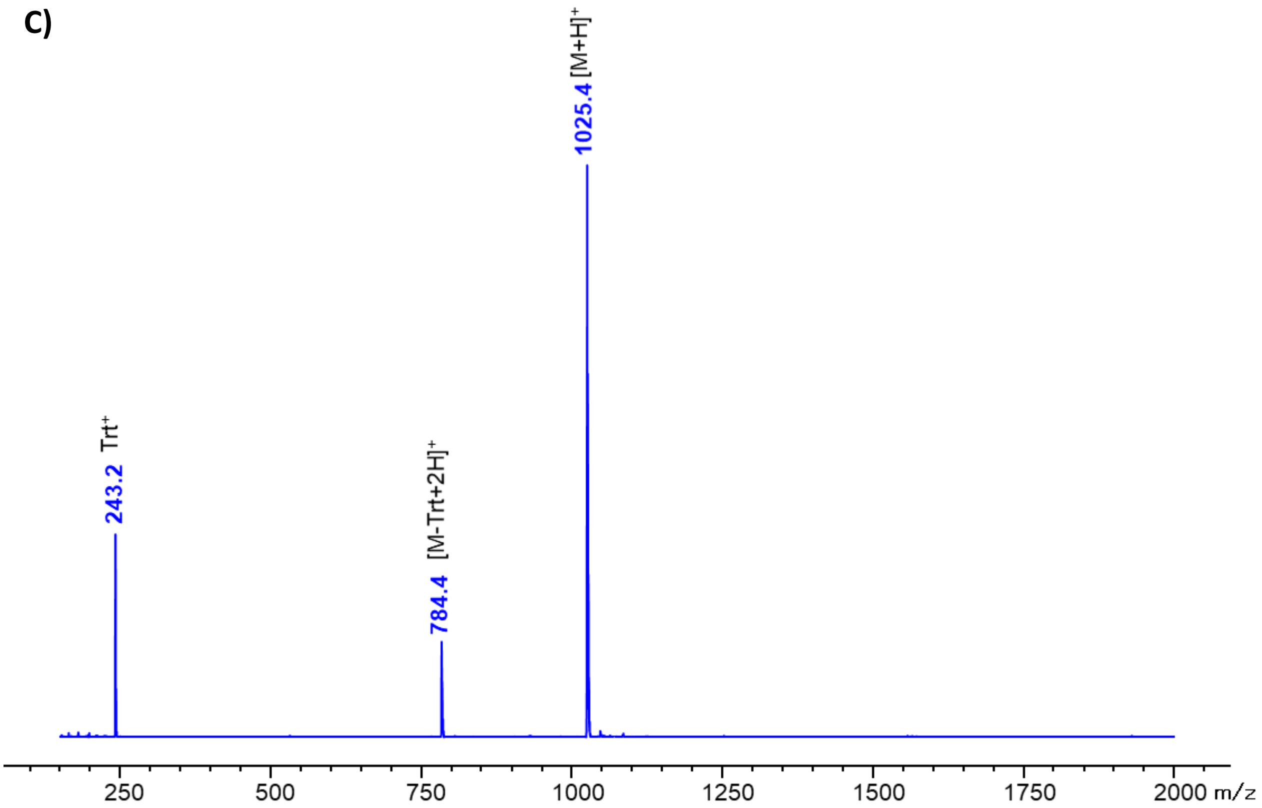

Figure S33. HPLC traces of (A) crude (Chromolith, gradient: 5-95% Solv. B over 7 min.) and (B) purified compound **5d** (λ = 214 nm). (C) ESI-MS spectrum of purified compound **5d**.

6-5 Synthesis of peptide spacer 5e

- **SPPS and cleavage**

Sequence Pent-PPPPPPPPPPPPC-NH_2 (**5e-SH**)The peptide was synthesized using protocol PS1. Cleavage was performed following protocol PS2.

Elongation yield: 85%. Determined by the ratio between the quantity of fluorenylpiperidine released during initial Fmoc deprotection and the quantity released during the Fmoc deprotection of the C-terminal Ala residue (UV titration at 301 nm, ε = 7800 $L \cdot mol^{-1} \cdot cm^{-1}$).

ESI-MS: $[M+H]^+$ monoisotopic mass calculated for $C_{68}H_{97}N_{14}O_{14}S$: 1365.7, found: 1366.6.

HPLC analysis: t_R = 2.42 min. (Chromolith, gradient: 15-35% Solv. B over 5 min.).

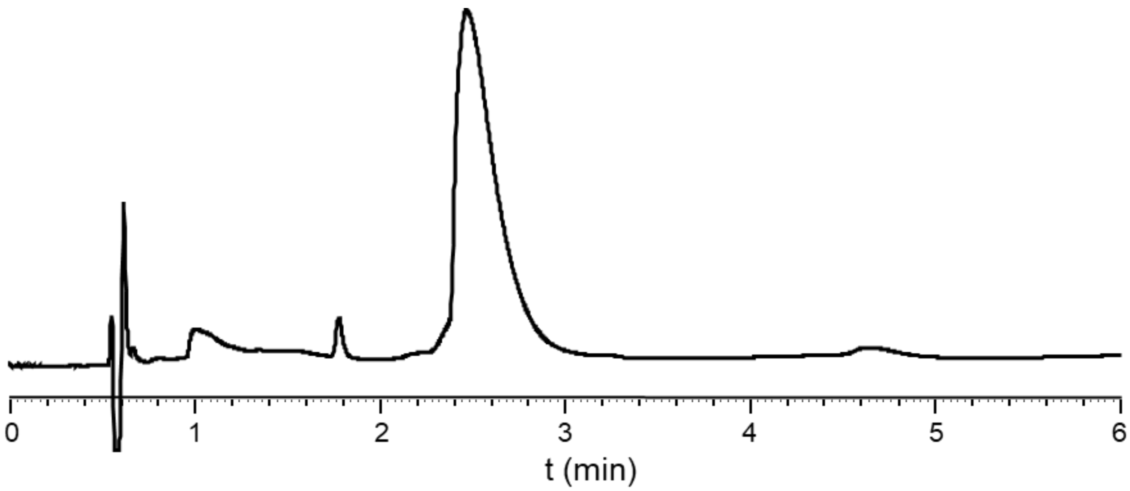

Figure S34. HPLC trace of crude compound **5e-SH** (λ = 214 nm).

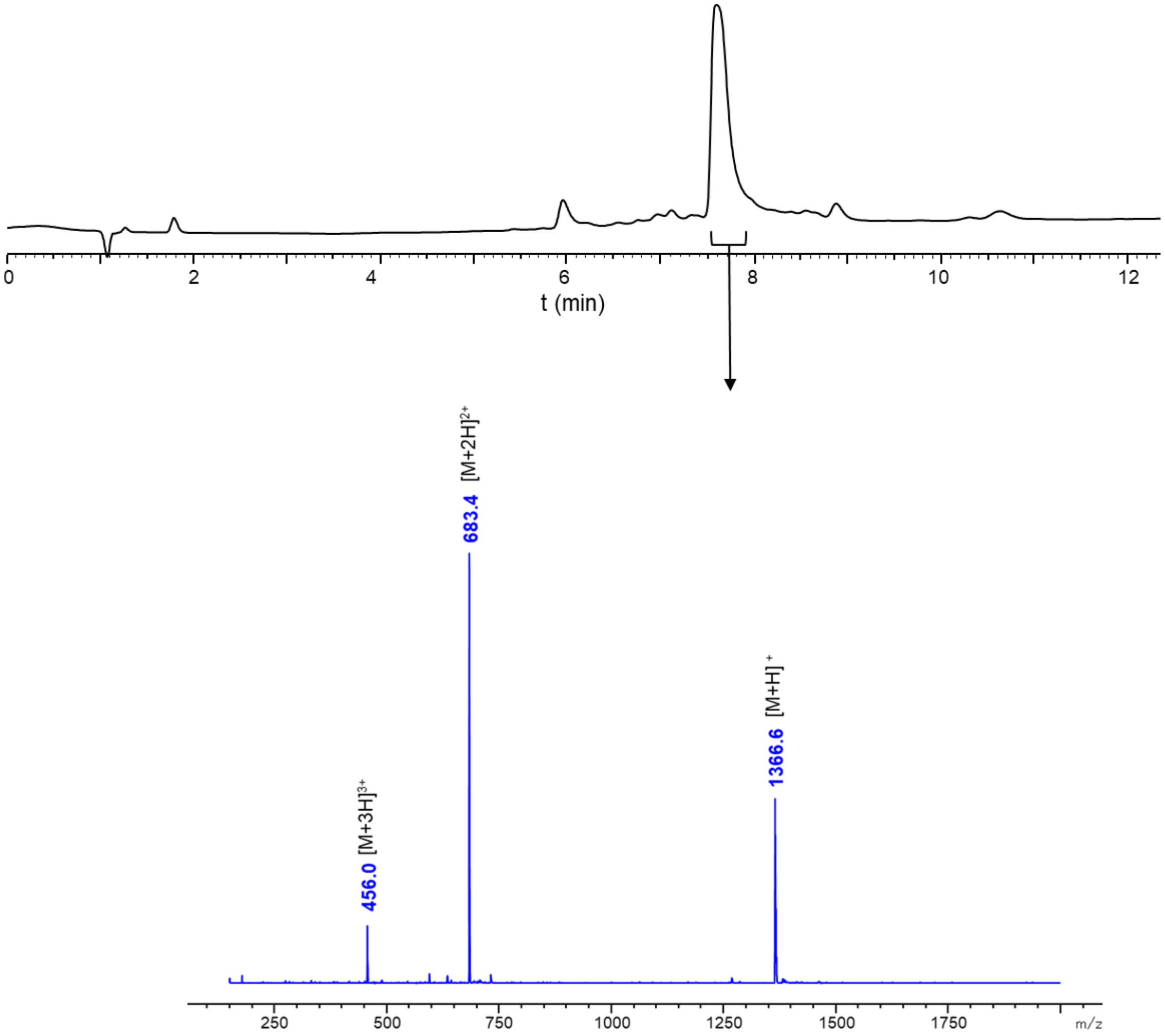

Figure S35. LC-MS analysis of crude compound **5e-SH**: A) HPLC trace (λ = 214 nm) and B) ESI-MS spectrum corresponding to the time window indicated as a black arrow (sum of spectra, 4.68-4.92 min).

- **Trityl protection**

Sequence: Pent-PPPPPPPPPPPPC(Trt)-NH_2 (**5e**)

Cysteine residue tritylation was performed using protocol PS4.

ESI-MS: $[M+H]^+$ monoisotopic mass calculated for $C_{87}H_{111}N_{14}O_{14}S_1$: 1607.8, found: 1609.6.

HPLC analysis: t_R = 5.11 min. (Chromolith, gradient: 5-80% B over 7 min.).

HPLC purification: Nucleosil C18, gradient: 30-60% Solv. B over 60 min.; 49% yield.

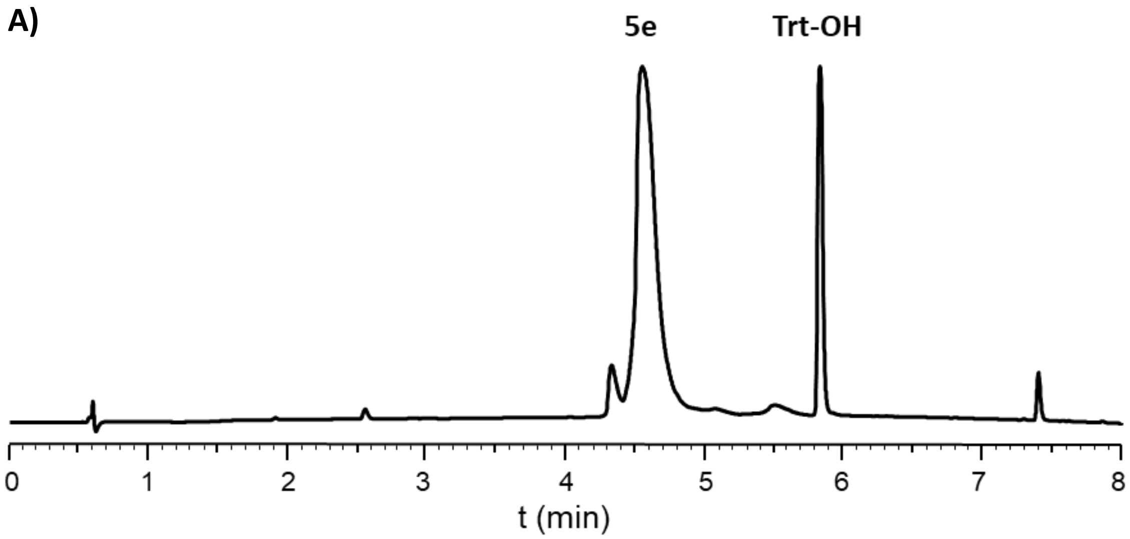

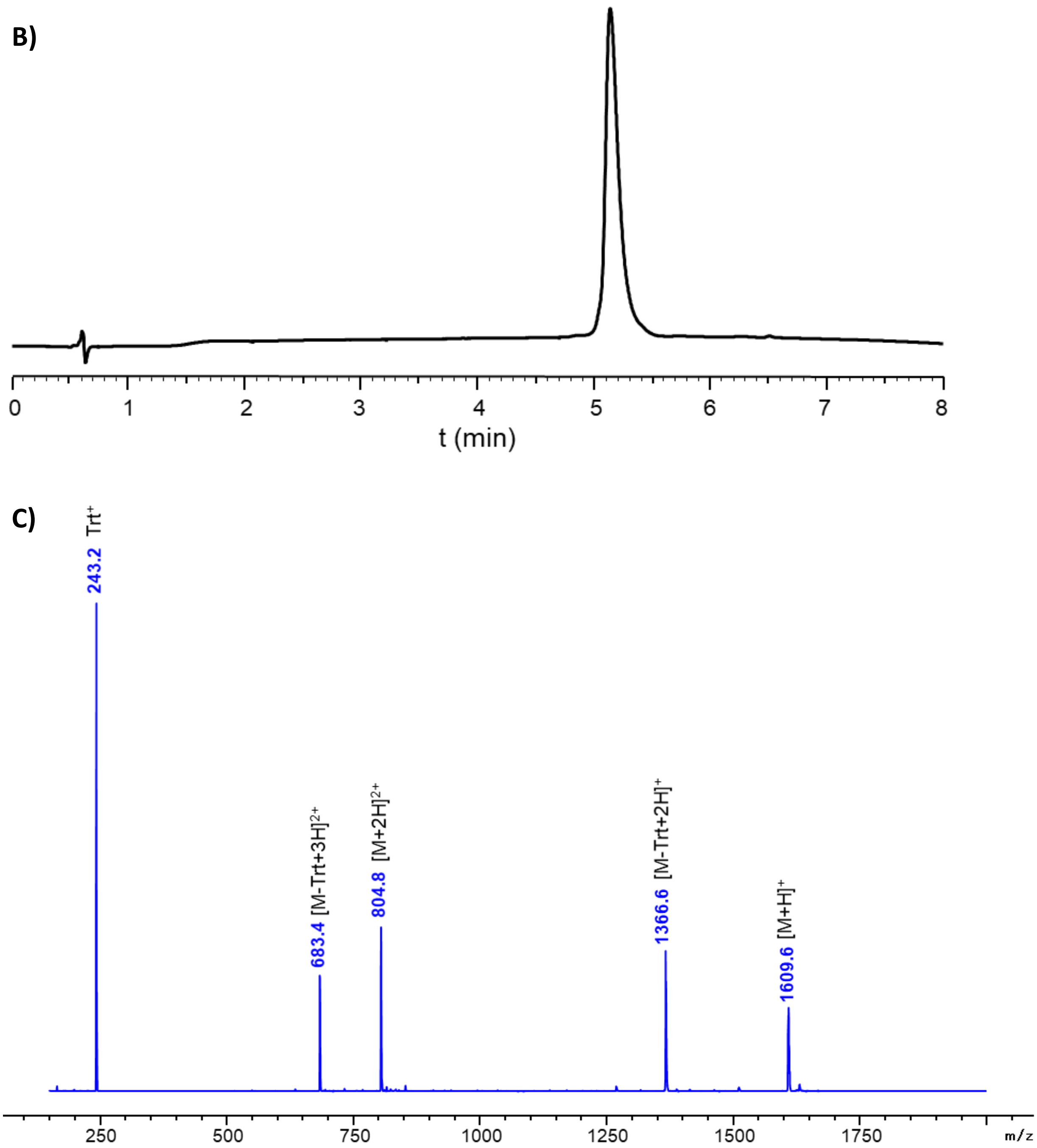

Figure S36. HPLC traces of (A) crude (Chromolith, gradient: 5-95% Solv. B over 7 min.) and (B) purified compound **5e** (λ = 214 nm). (C) ESI-MS spectrum of purified compound **5e**.

6-5 Evidences of *N-O* acyl shift products in linkers 5a-5c, and optimization of conditions to reverse it.

- **Evidences of *N-O* acyl shift products in linkers 5a-5c.**

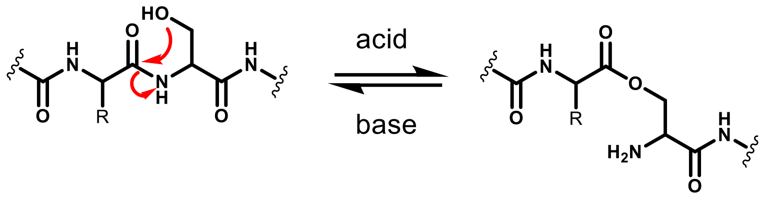

Scheme S3. Reversible *N→O* acyl shift in serine-containing peptides.

After purification of compound **5b**, we found that, contrary to what was suggested by the HPLC chromatogram (Figure S27, A), the compound was not pure. Under LC-MS conditions, using 0.1% formic acid as the mobile phase additive instead of 0.1% TFA, an additional peak was observed. Both peaks displayed identical molecular masses but showed distinct multicharged ions patterns (Figure S27, C and D), indicating the presence of isomeric species. This side product was detected for all $(GGGGS)_n$ spacers, although in variable proportions, and correlated with the asymmetrical peak shape observed in the HPLC chromatogram (Figure S27, A). The difference observed between TFA- and formic acid-containing elution systems led us to hypothesize that this minor species could result from an *N→O* acyl shift: indeed, a *N→O* acyl shift amine product would have one trifluoroacetate or formate counter-ion, whereas the uncharged amide form would not. The trifluoroacetate counterion is expected to interact more strongly with the stationary phase than formate, giving a plausible explanation of the observed differences in relative retention times of both species under the two elution systems. This assumption was further supported by the effect of a basic treatment. Three basic conditions were tested, PBS 10X, NH_4HCO_3 and DIPEA. All showed comparable efficiency in reversing the shift; however, NH_4HCO_3 resulted in less clean HPLC traces, while DIEA proved to be the most straightforward and effective to implement.

A)

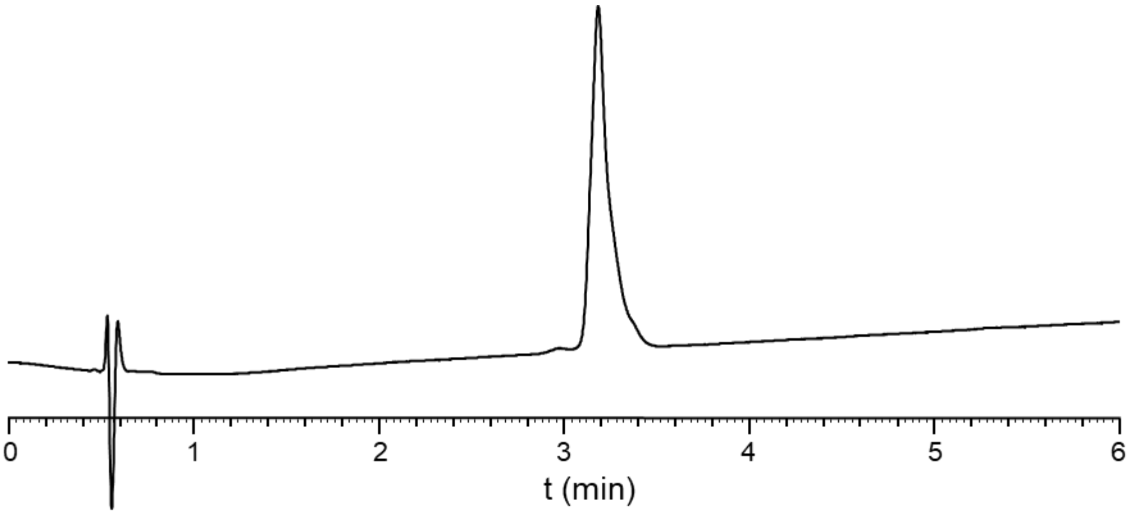

B)

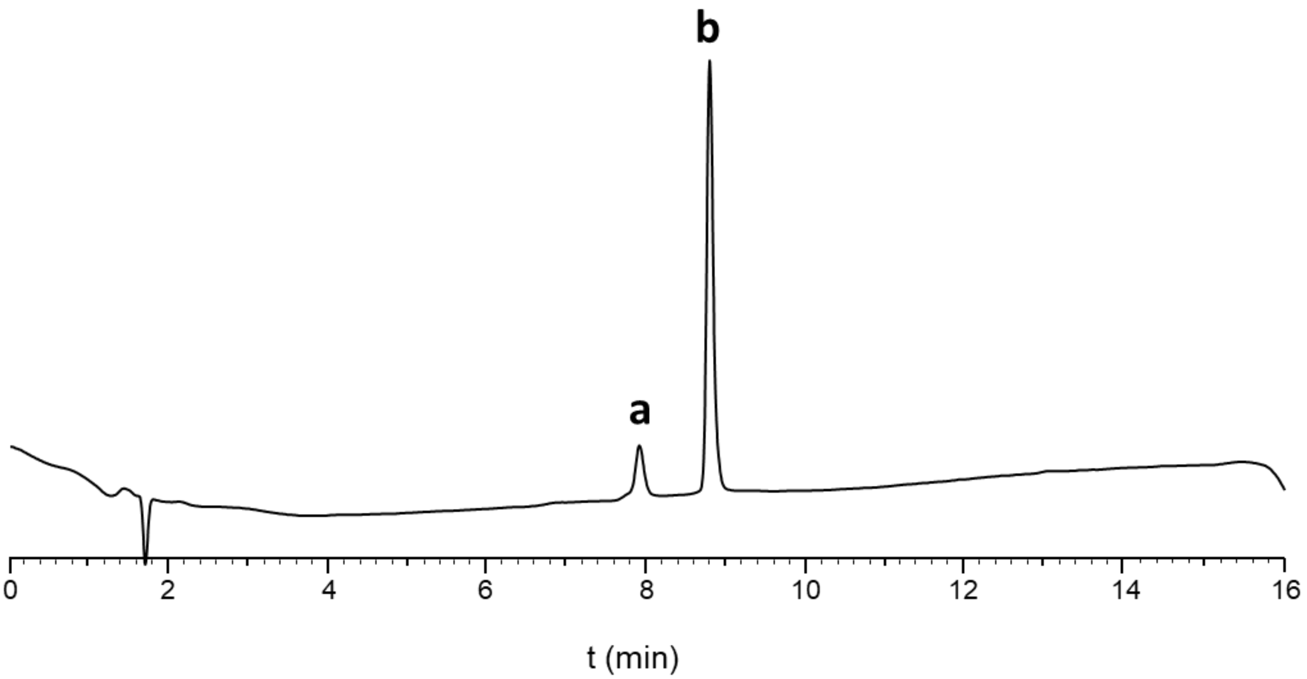

C)

MS spectrum of peak a

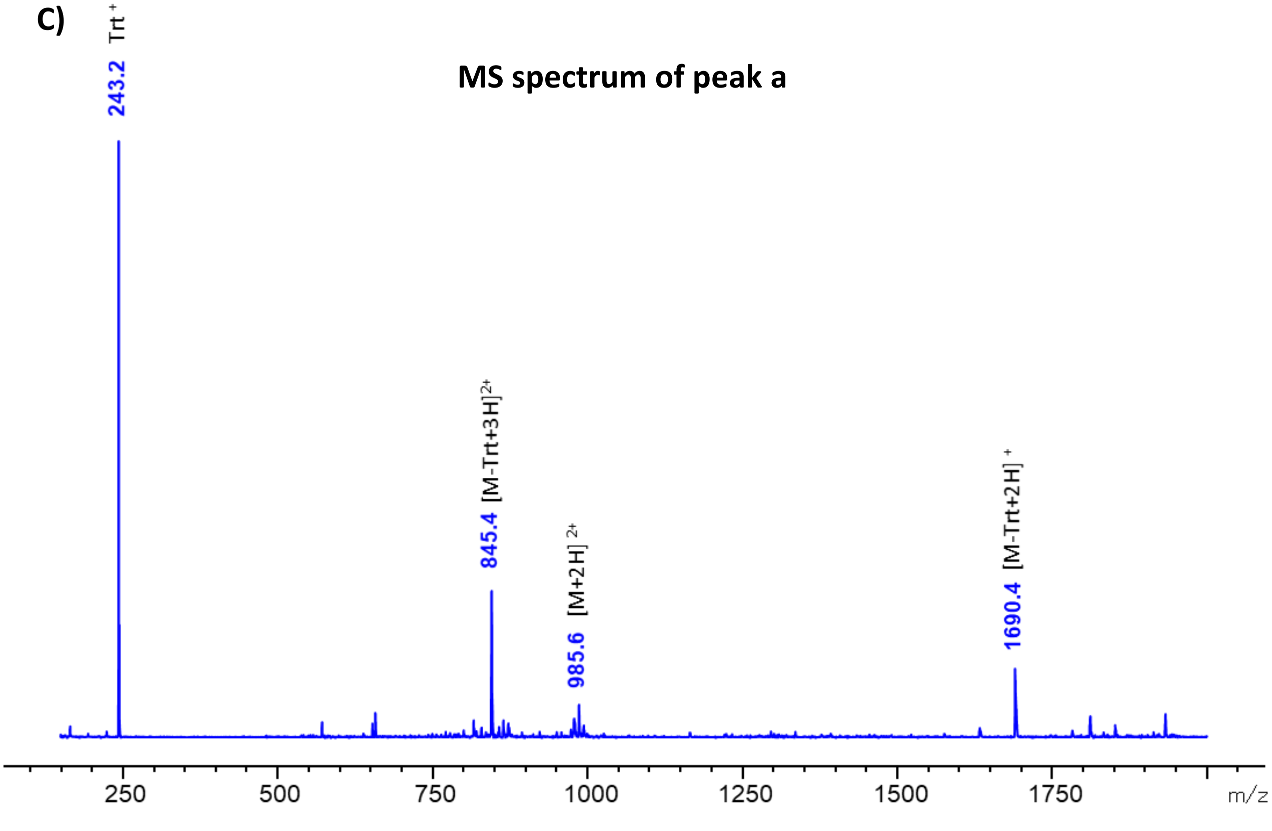

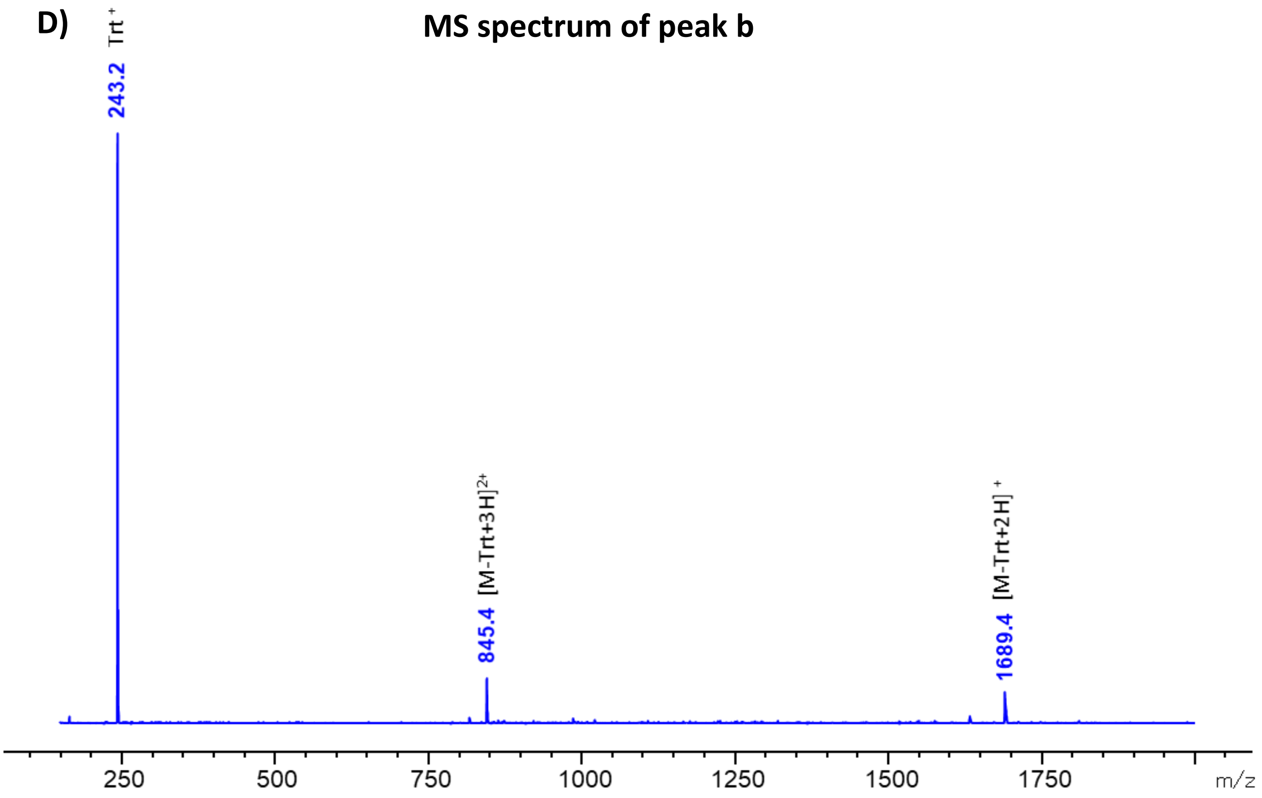

Figure S37. HPLC trace of compound **5b** observed during semi-preparative purification in (A) Chromolith, gradient: 25-40% Solv. B over 5 min. and in (B) Aeris Widepore XB-C18, gradient: 3% Solv.B' during 0.6 min then 3-50% Solv. B' over 10.8 min. at 60 °C (λ = 214 nm). ESI-MS spectrum of (C) corresponding *N*→*O* acyl shifted compound **5b** (peak a) and of (D) compound **5b** (peak b).

7- Synthesis of "DRP-spacer-thiol" constructs 6a-6e

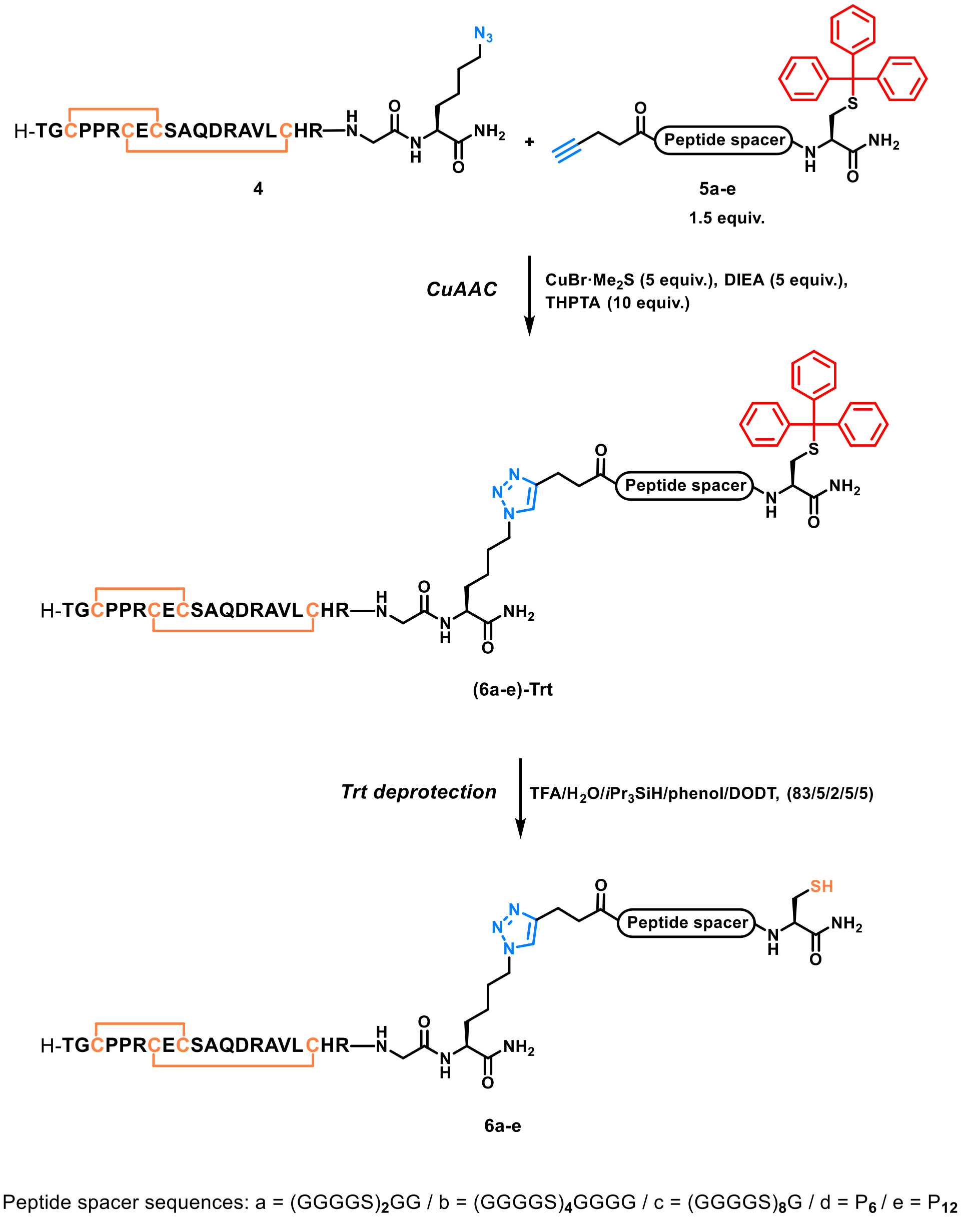

Scheme S4. General scheme of the CuAAC reaction.

A series of experiments were conducted by varying key parameters including the copper source, ligand, reducing agent, solvent, temperature and pH. The results are summarized in table S4. Due to the limited quantity of GGGGS-containing spacers starting material, the experiments were performed using either **5a**, **5b** or **5c** (see table S4 footnotes). Optimal conversion was achieved at 3.35 mM using CuBr·Me$_2$S (5 equiv.) in presence of THPTA (10 equiv.) and DIPEA (5equiv.) in DMSO at 37 °C for 2 hours. Notably, deviations from these conditions led to either lower yields or incomplete conversion and copper oxidation, as evidenced by a blue-green color.

Table S4. Different conditions used for the CuAAC reaction. All reactions were performed for 2 h, at a 3.35 mM peptide concentration.

Entry	Copper catalyst		THPTA (equiv.)	Na ascorbate (equiv.)	DIPEA (equiv.)	Solvent	Temperature	Results
	CuX	Equiv.						
**1**[a]	$CuSO_4$	2	6	8.1	-	HFIP/HEPES pH 7.2 : 1/1	RT	Very low conversion rate
**2**[b]	$CuSO_4$	2	6	8.1	-	HFIP/HEPES pH 7.2 : 1/3	RT	Very low conversion rate
**3**[b]	$CuSO_4$	6	10	20	2	HFIP/HEPES pH 7.2 : 1/1	RT	Precipitation / Cu oxidation
**4**[c]	$CuSO_4$	2	6	8.1	-	HFIP/HEPES pH8 : 1/1	RT	Precipitation / Trt deprotection
**5**[c]	$CuBr.Me_2S$	5	-	-	-	DMSO	RT	Very low conversion rate
**6**[c]	$CuBr.Me_2S$	5	10	-	5	DMSO	RT	21% conversion
**7**[c]	$CuBr.Me_2S$	5	10	-	5	DMSO	37 °C	Full conversion

[a] Spacer **5a** $(GGGGS)_2$GGC(Trt), [b] Spacer **5c** $(GGGGS)_8$GC(Trt), [c] Spacer **5b** $(GGGGS)_4$GGGGC(Trt)

7-1 Synthesis of 6a

- **CuAAC**

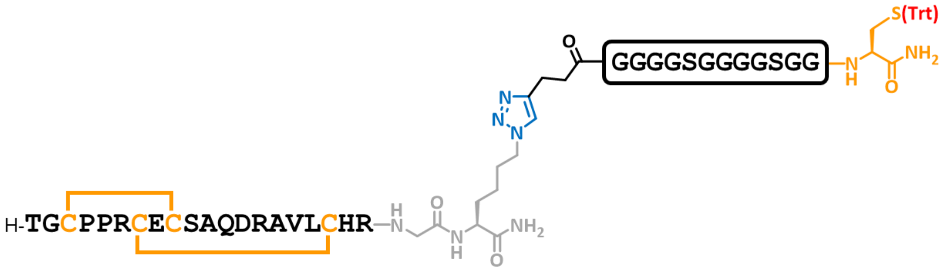

6a-Trt

Synthesis of **6a-Trt** was performed following protocol PS5.

ESI-MS: [M] average mass calculated. for $C_{147}H_{220}N_{52}O_{45}S_5$: 3596.0, found: 3595.1 (deconvoluted).

HPLC purification: Nucleosil C18, gradient: 20-50% Solv. B over 20 min.; 37% yield.

HPLC analysis: t_R = 3.98 min. (Chromolith, gradient: 5-80% Solv. B over 7 min.).

A)

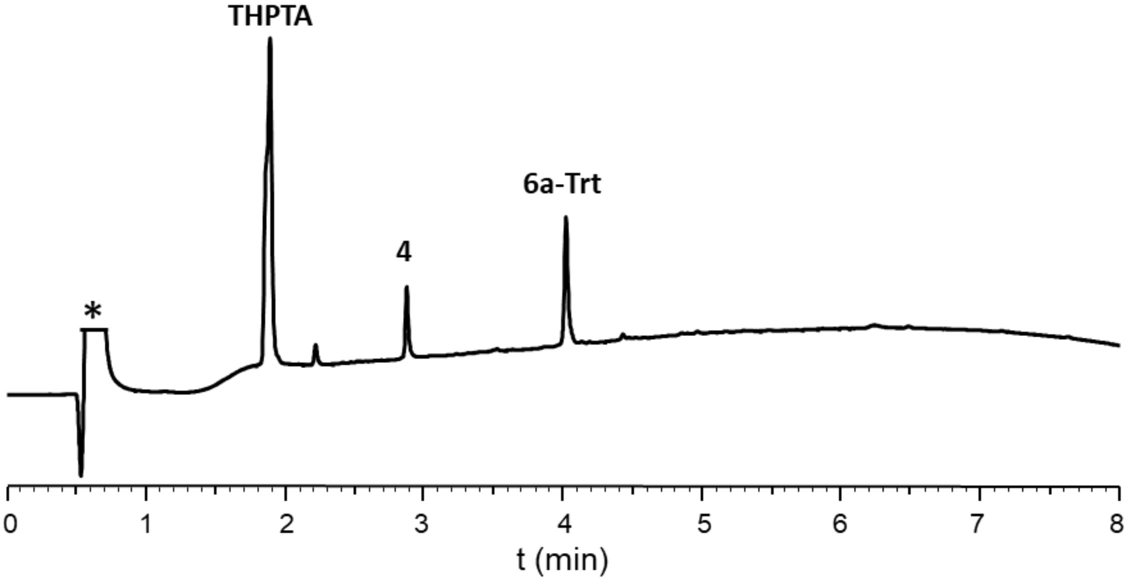

*DMSO

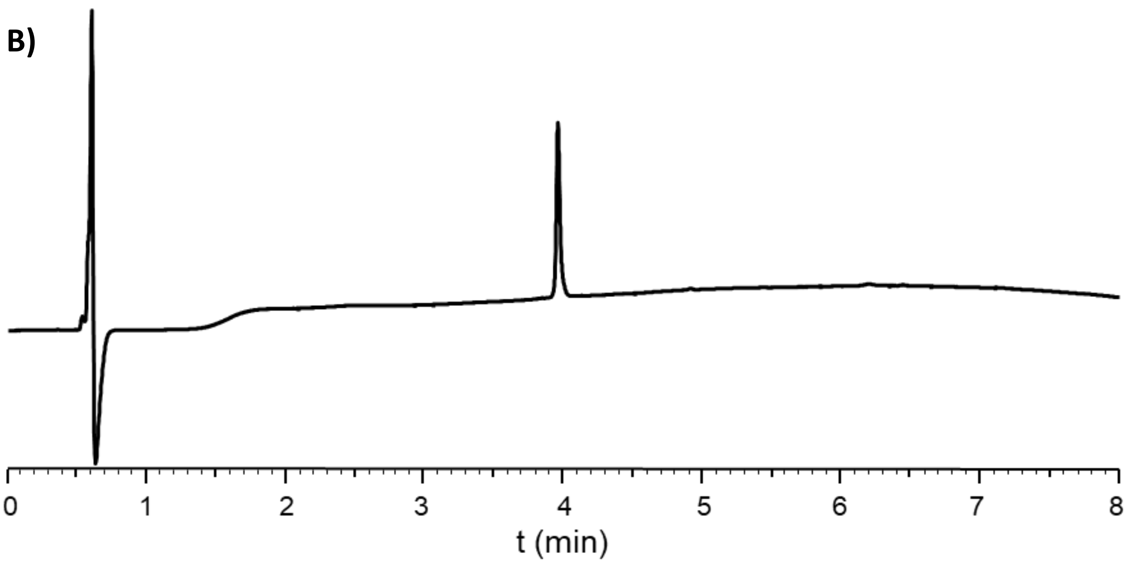

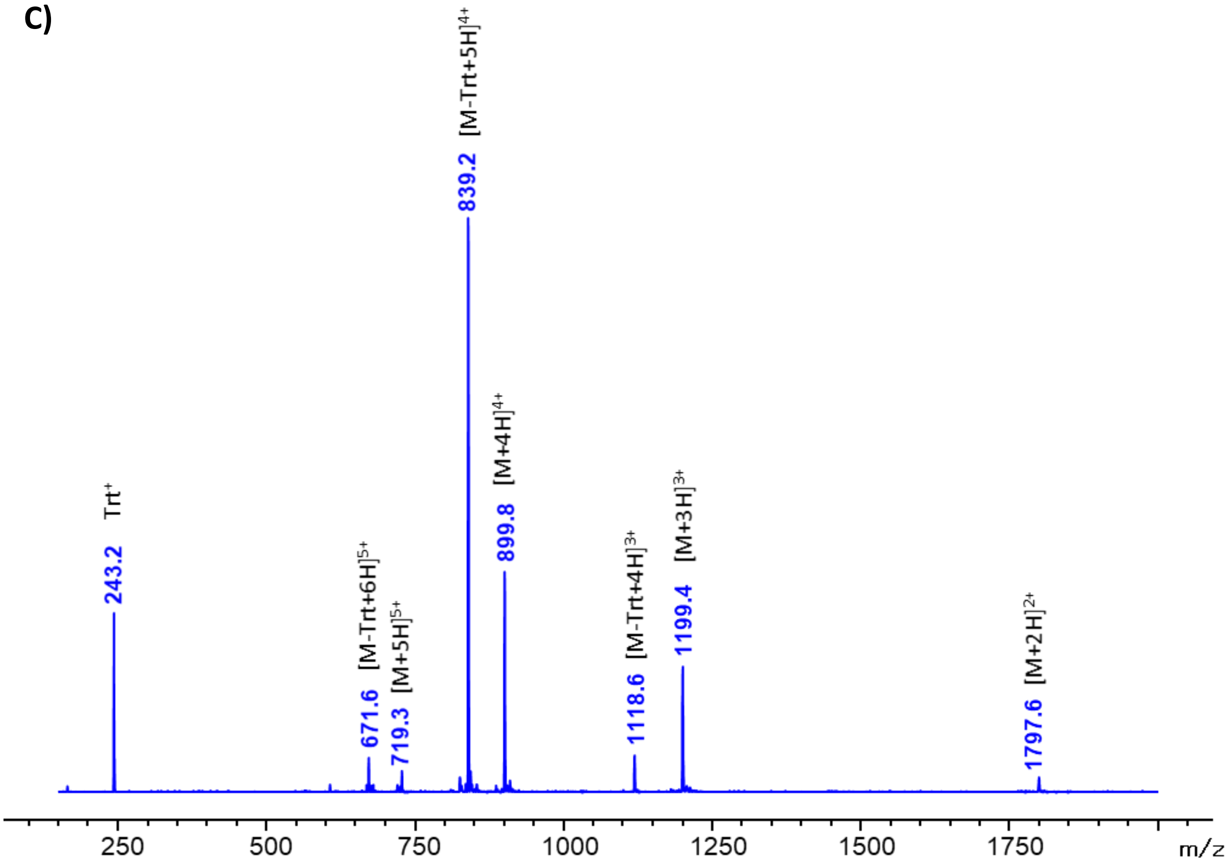

Figure S38. HPLC traces of (A) crude and (B) purified compound **6a-Trt** (λ = 214 nm). (C) ESI-MS spectrum of purified compound **6a-Trt**.

- **Trityl deprotection**

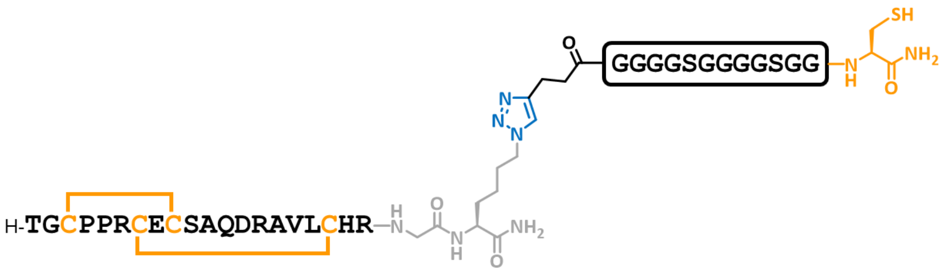

6a

Synthesis of **6a** was performed following protocol PS6.

ESI-MS: [M] average mass calculated for $C_{128}H_{206}N_{52}O_{45}S_5$: 3353.7, found: 3352.9 (deconvoluted).

HPLC purification: The crude peptide was used for disulfide pattern disruption assay without any further purification.

HPLC analysis: t_R = 2.58 min. (Chromolith, gradient: 10-30% Solv. B over 5 min.).

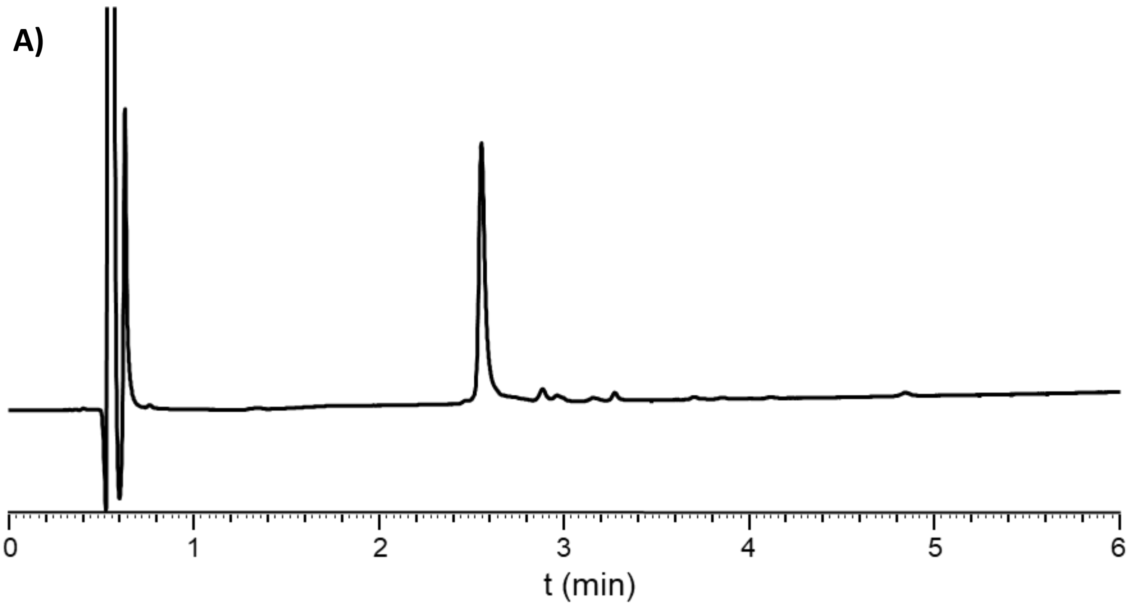

B)

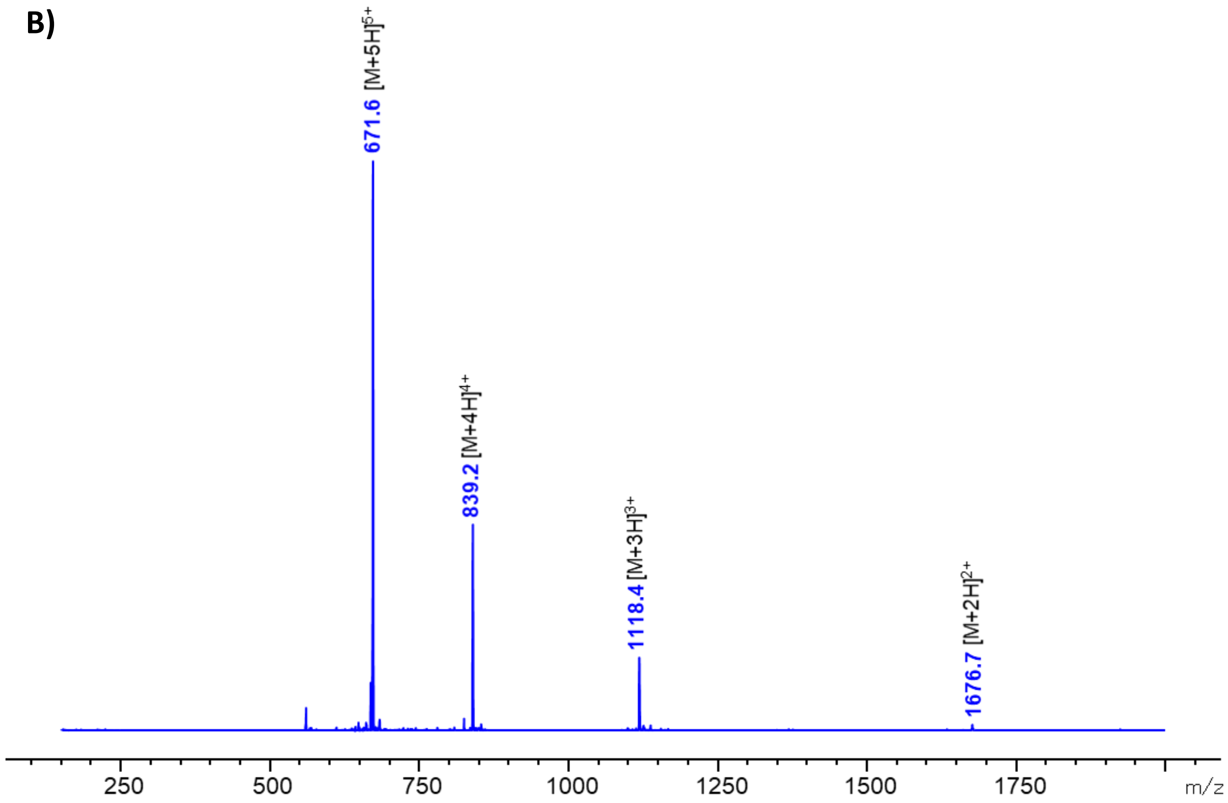

Figure S39. (A) HPLC trace of crude compound **6a** (λ = 214 nm). (B) ESI-MS spectrum of crude compound **6a**.

7-2 Synthesis of 6b

- **CuAAC**

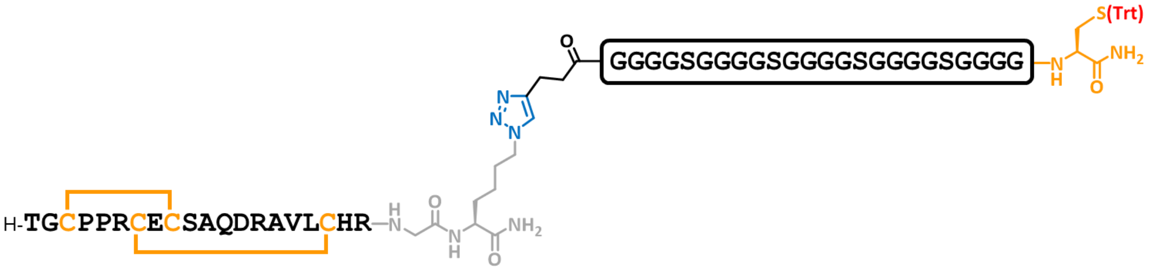

6b-Trt

Synthesis of **6b-Trt** was performed following protocol PS5.

ESI-MS: [M] average mass calculated for $C_{173}H_{260}N_{64}O_{59}S_5$: 4340.6, found: 4339.8 (deconvoluted).

HPLC purification: Nucleosil C18, gradient: 25-40% Solv. B over 20 min.; 31% yield.

HPLC analysis: t_R = 3.98 min. (Chromolith, gradient: 5-80% Solv. B over 7 min.).

A)

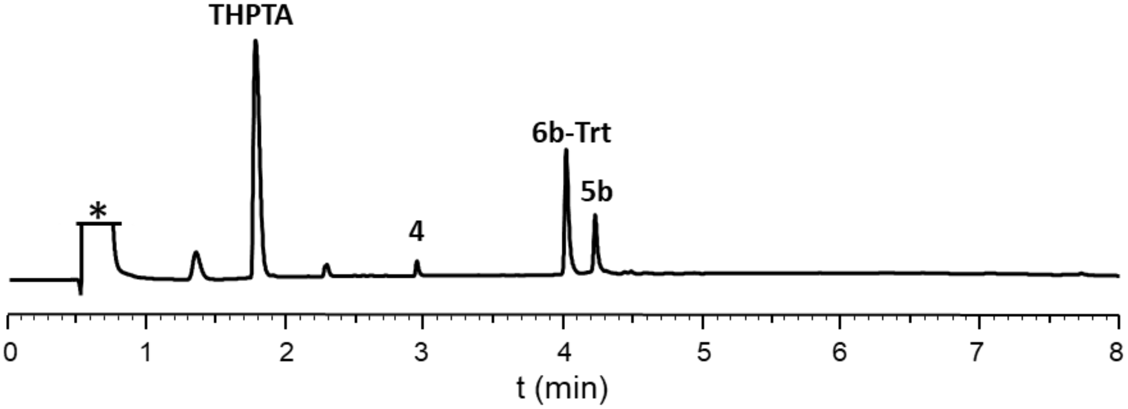

*DMSO

B)

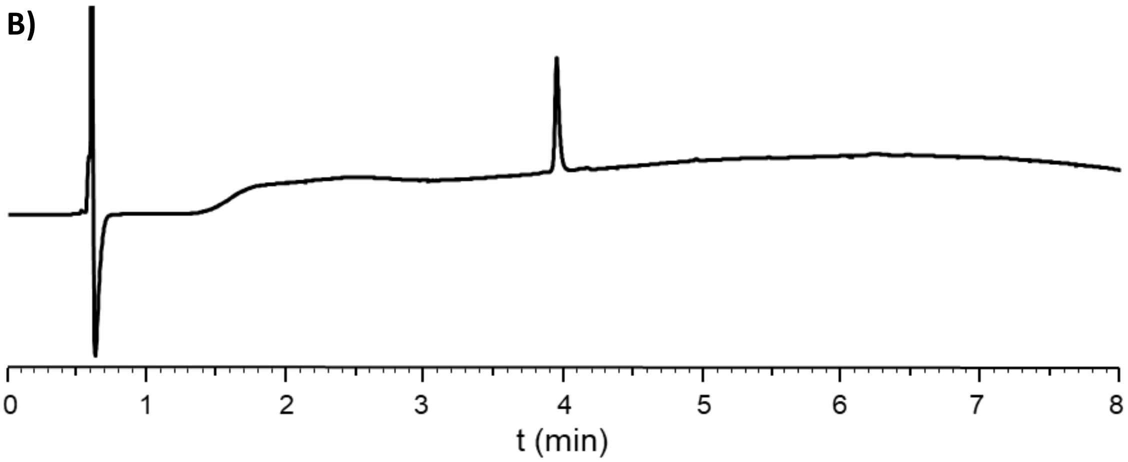

C)

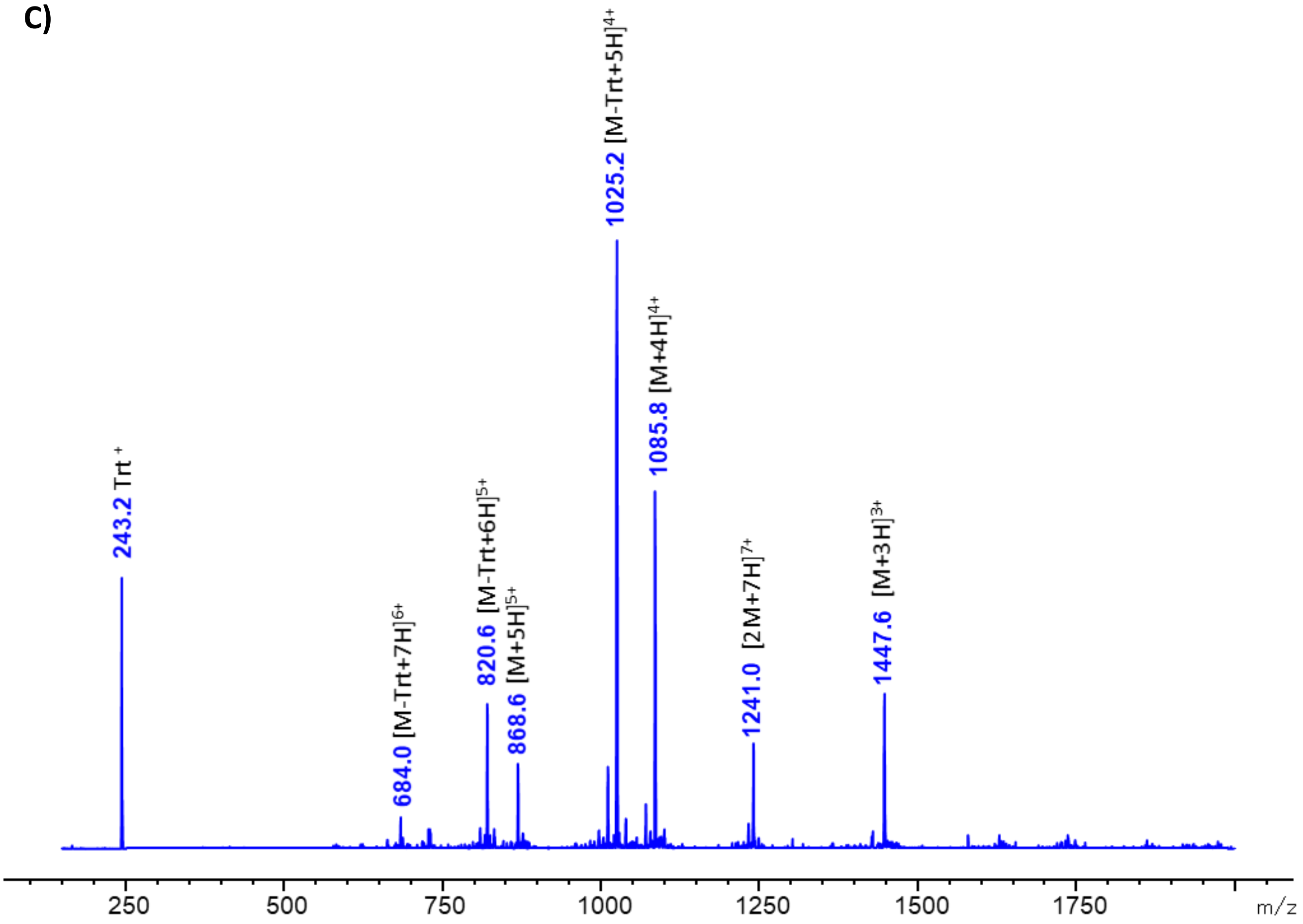

Figure S40. HPLC traces of (A) crude and (B) purified compound **6b-Trt** (λ = 214 nm). (C) ESI-MS spectrum of purified compound **6b-Trt**.

- **Trityl deprotection**

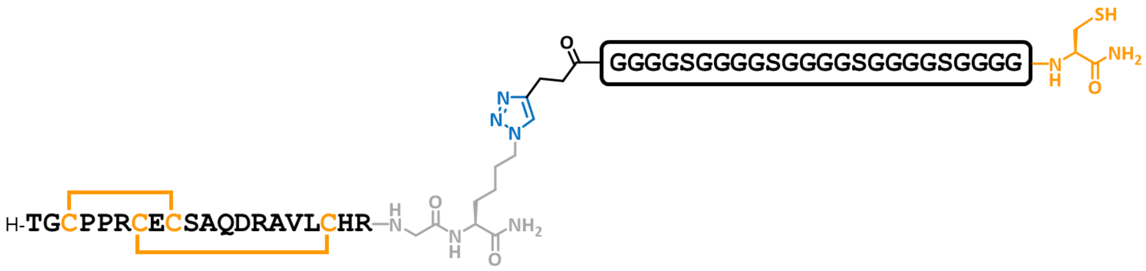

6b

Synthesis of **6b** was performed following protocol PS6.

ESI-MS: [M] average mass calculated for $C_{154}H_{246}N_{64}O_{59}S_5$: 4098.3, found: 4097.5 (deconvoluted).

HPLC purification: The crude peptide was used for disulfide pattern disruption assay without any further purification.

HPLC analysis: t_R = 2.46 min (Chromolith, gradient: 10-30% Solv. B over 5 min.).

A)

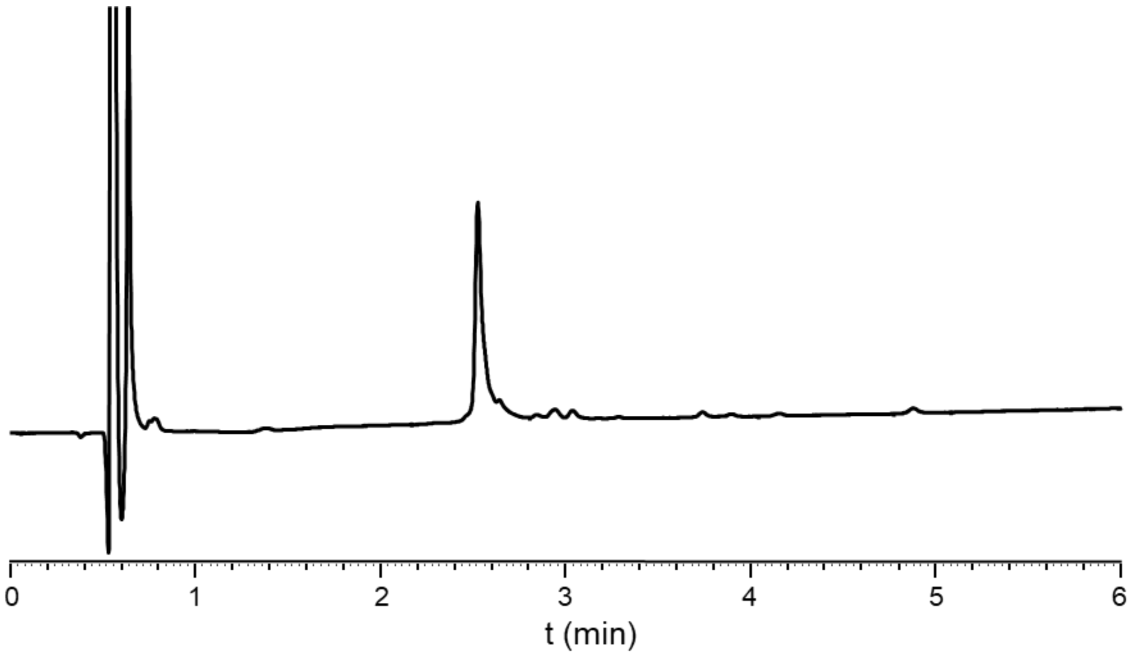

B)

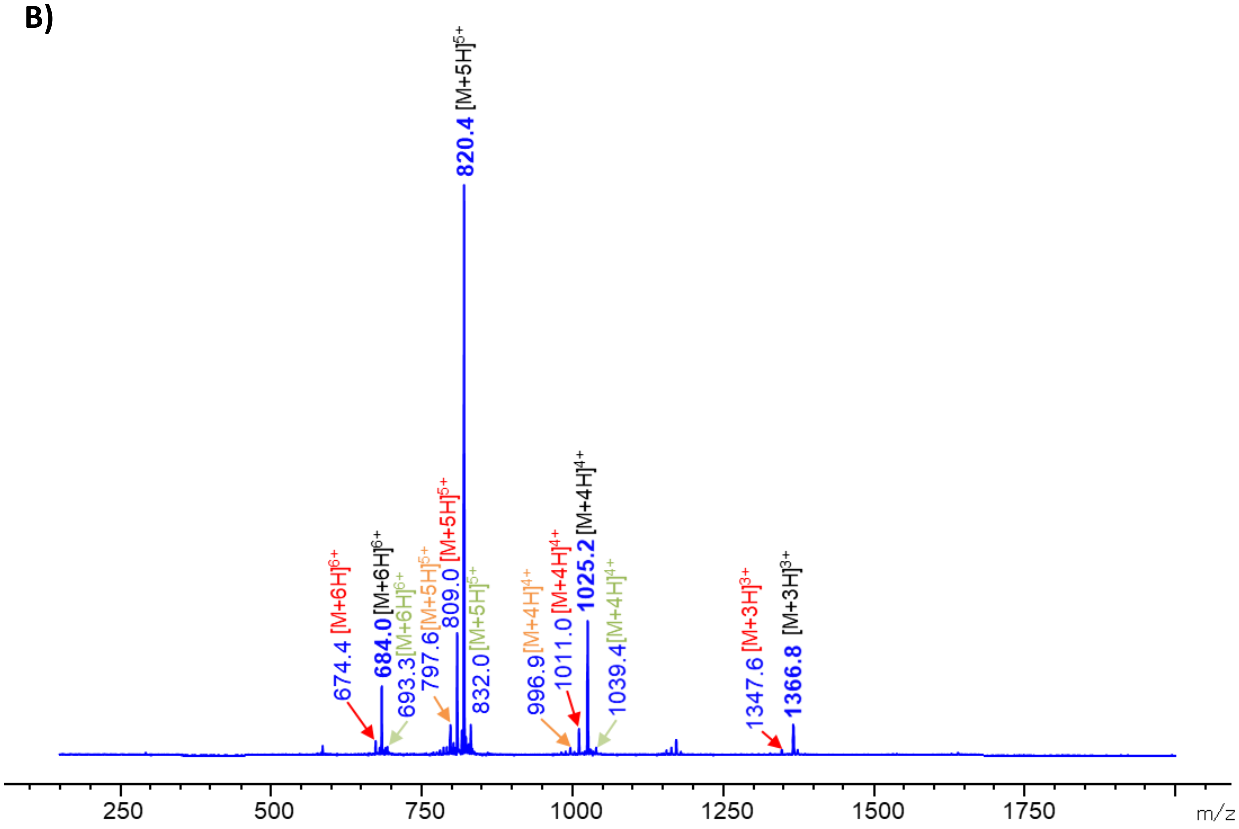

Figure S41. (A) HPLC trace of crude compound **6b** (λ = 214 nm). (B) ESI-MS spectrum of crude compound **6b.** Additional MS peaks were observed, consistent with deletion of one Gly residue (highlighted in red), deletion of two Gly (orange), or overcoupling of one Gly (green). [M] calculated/found: 4041.3/4040.0; 3984.2/3983.0; 4155.4/4154.7, respectively. These impurities arise from the SPPS of the peptide-based linker, as evidenced by the presence of masses with similar M in linker **5b-SH** and **5b**, as well as in *S*-Trt conjugate **6b-Trt**.

7-3 Synthesis of 6c

- **CuAAC**

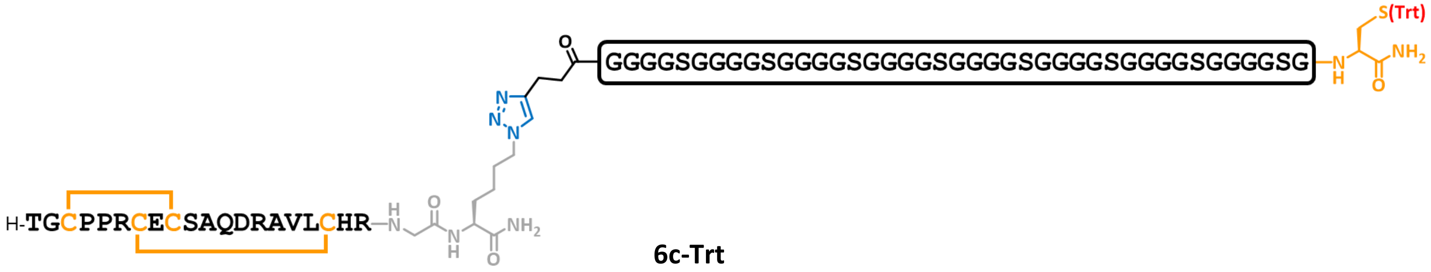

6c-Trt

Synthesis of **6c-Trt** was performed following protocol PS5.

ESI-MS: [M] average mass calculated for $C_{211}H_{319}N_{81}O_{80}S_5$: 5430.6, found: 5429.5 (deconvoluted).

HPLC purification: Nucleosil C18, gradient: 25-40% Solv. B over 20 min.; 37% yield.

HPLC analysis: t_R = 3.85 min. (Chromolith, gradient: 5-80% Solv. B over 7 min.).

A)

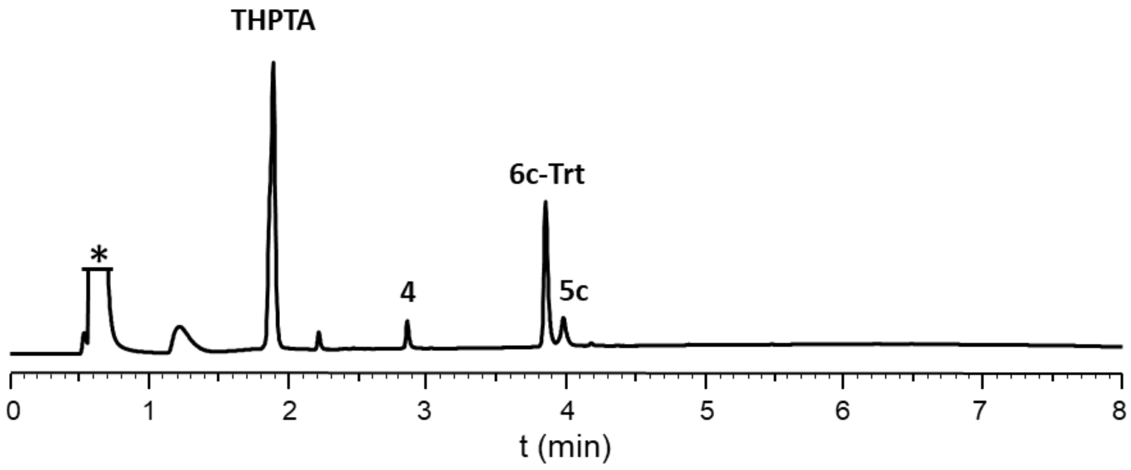

*DMSO

B)

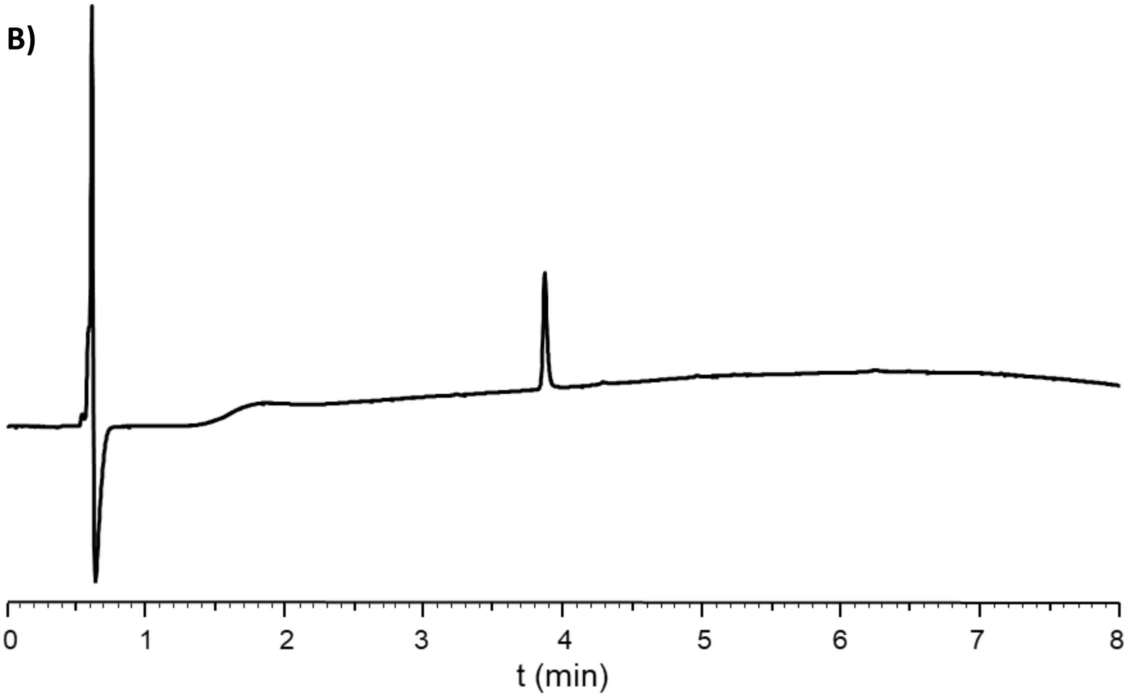

C)

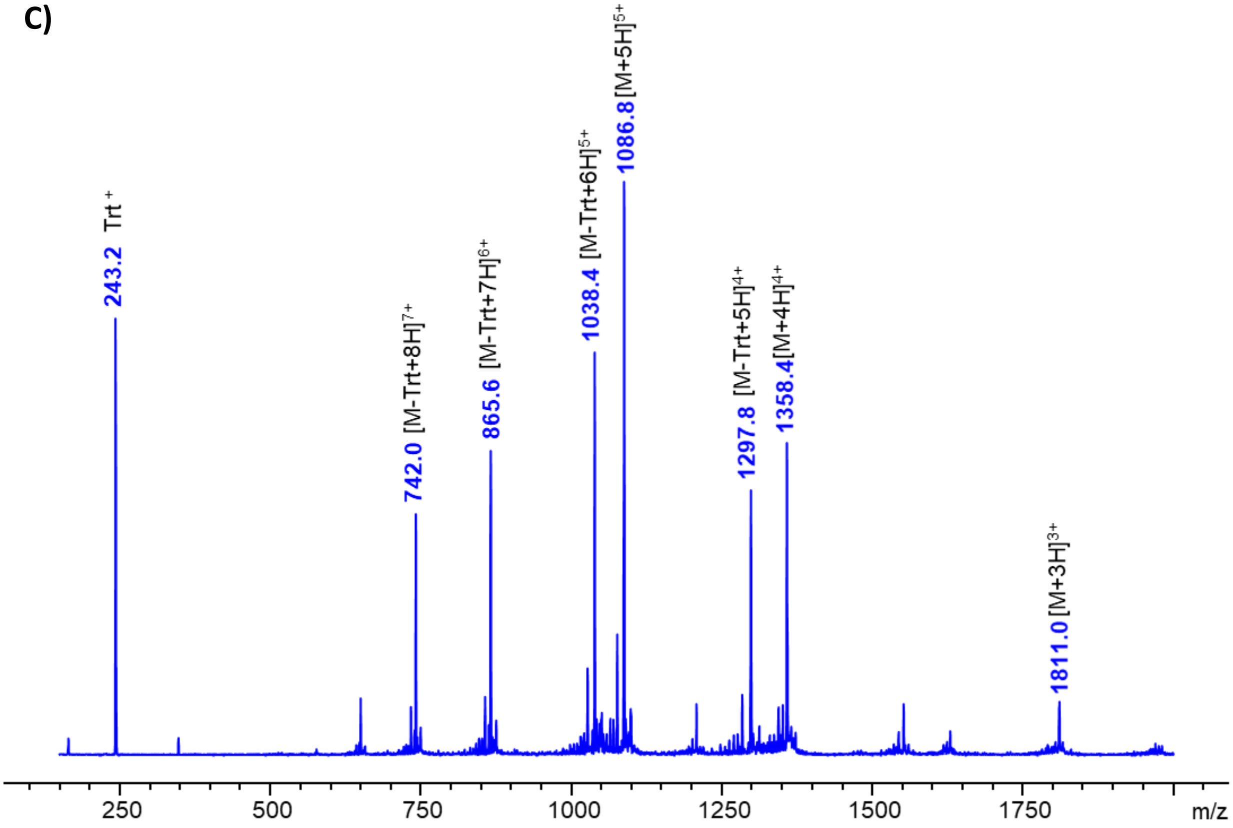

Figure S42. HPLC traces of (A) crude and (B) purified compound **6c-Trt** (λ = 214 nm). (C) ESI-MS spectrum of purified compound **6c-Trt**.

- **Trityl deprotection**

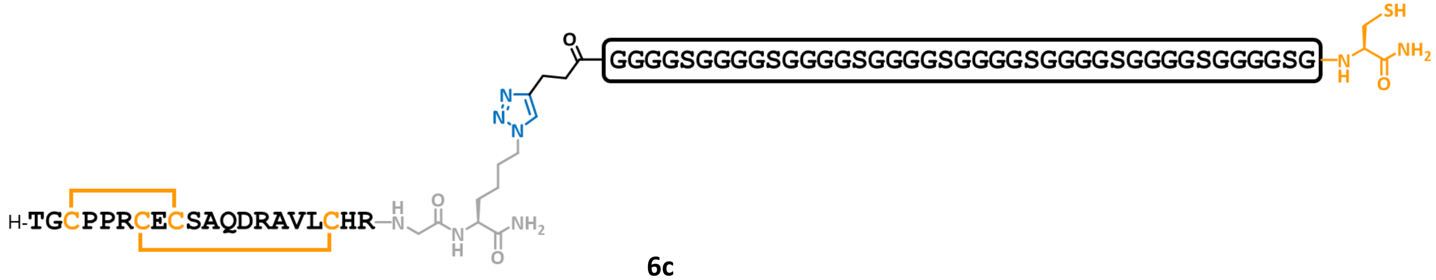

6c

Synthesis of **6c** was performed following protocol PS6.

ESI-MS: [M] average mass calculated for $C_{192}H_{305}N_{81}O_{80}S_5$: 5188.3, found: 5187.4 (deconvoluted).

HPLC purification: The crude peptide was used for disulfide pattern disruption assay without any further purification.

HPLC analysis: t_R = 2.19 min. (Chromolith, gradient: 10-30% Solv. B over 5 min.).

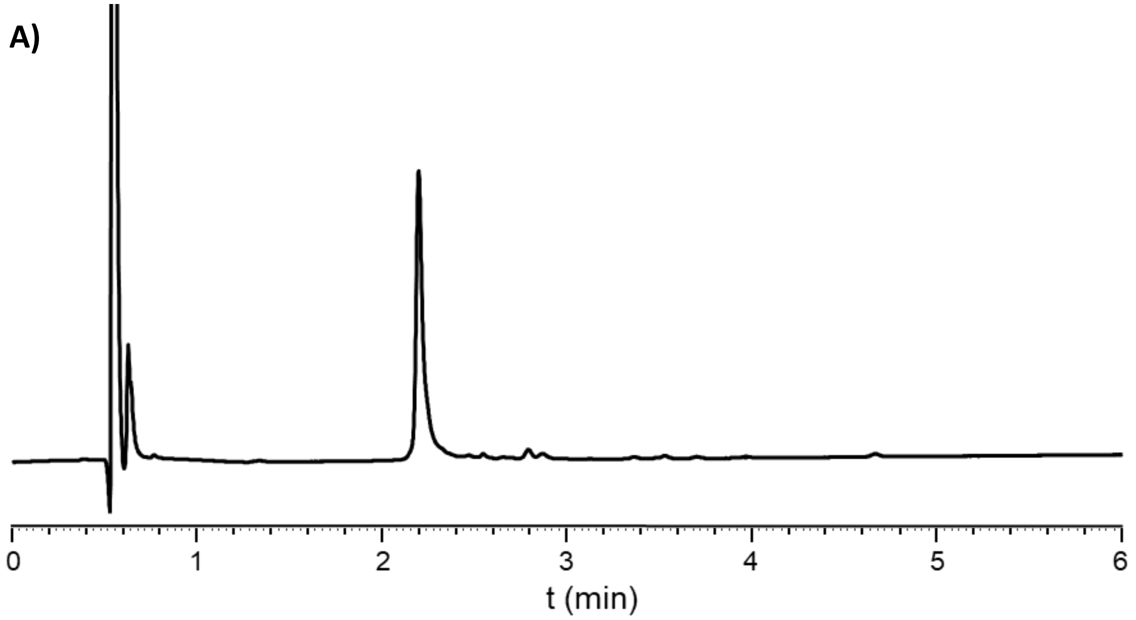

B)

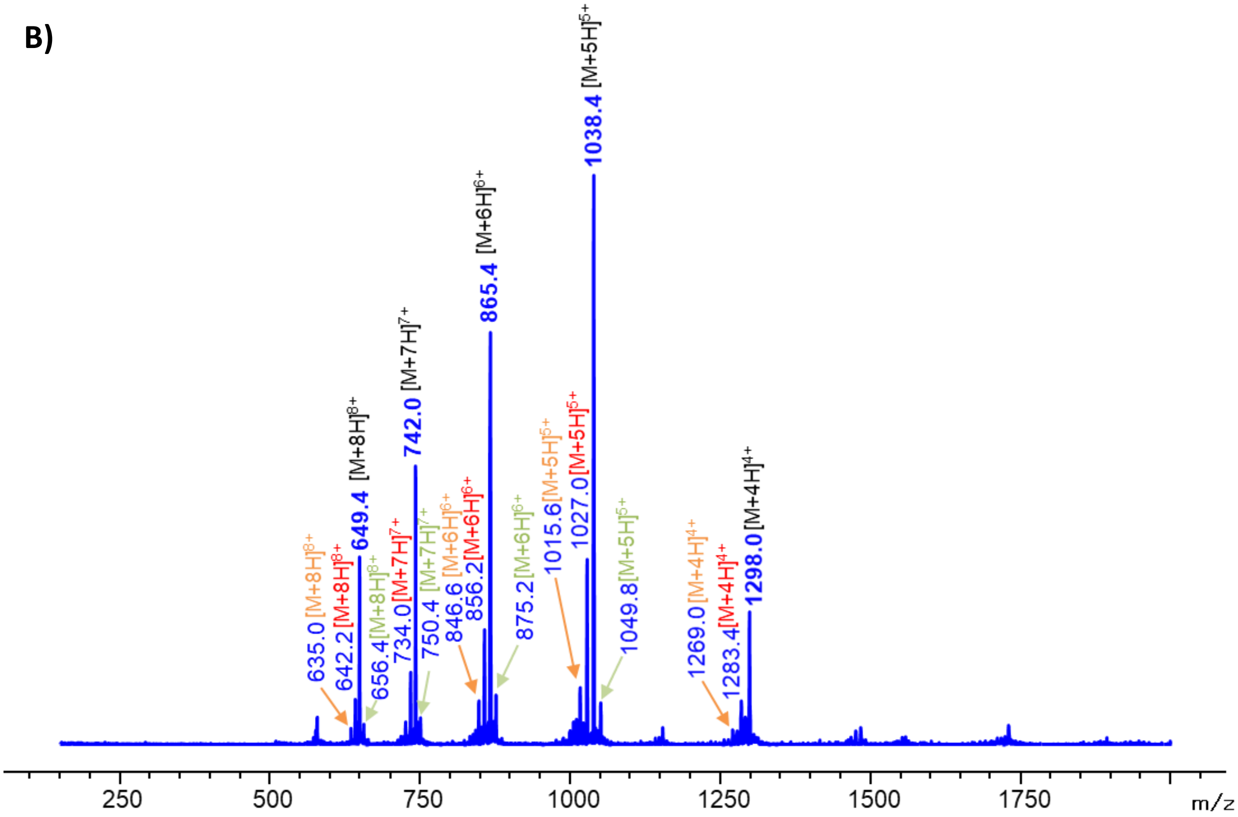

Figure S43. (A) HPLC trace of crude compound **6c** (λ = 214 nm). (B) ESI-MS spectrum of crude compound **6c.** Additional MS peaks were observed, consistent with deletion of one Gly residue (highlighted in red), deletion of two Gly (orange), or overcoupling of one Gly (green). [M] calculated/found: 5131.2/5130.3; 5074.2/5073.7; 5245.4/5244.6, respectively. These impurities arise from the SPPS of the peptide-based linker, as evidenced by the presence of masses with similar M in linker **5c-SH** and **5c**, as well as in *S*-Trt conjugate **6c-Trt**.

7-4 Synthesis of 6d

- **CuAAC**

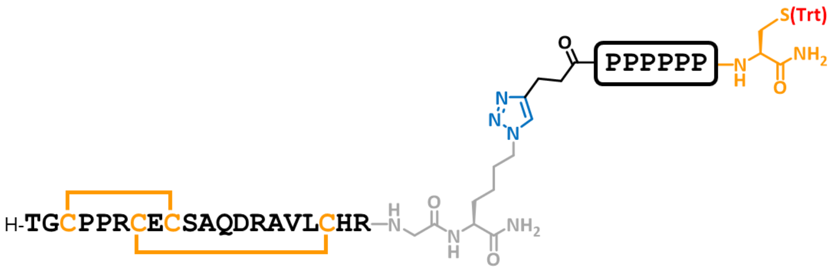

6d-Trt

Synthesis of **6d-Trt** was performed following protocol PS5.

ESI-MS: [M] average mass calculated for $C_{151}H_{222}N_{46}O_{37}S_5$: 3434.0, found: 3433.2 (deconvoluted).

HPLC purification: Nucleosil C18, gradient: 30-60% Solv. B over 20 min.; 67% yield.

HPLC analysis: t_R = 4.50 min. (Chromolith, gradient: 5-80% Solv. B over 7 min.).

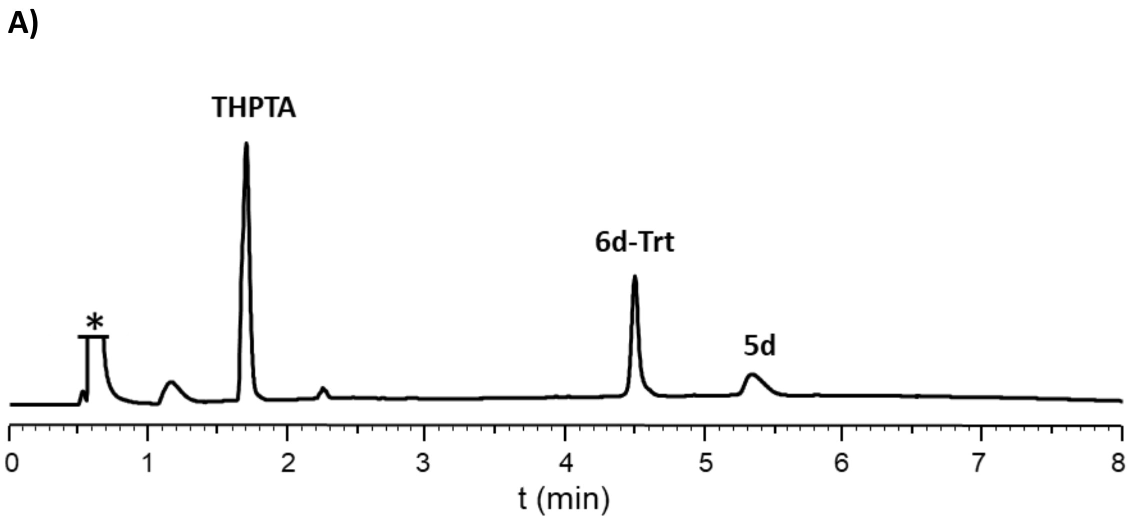

* DMSO

B)

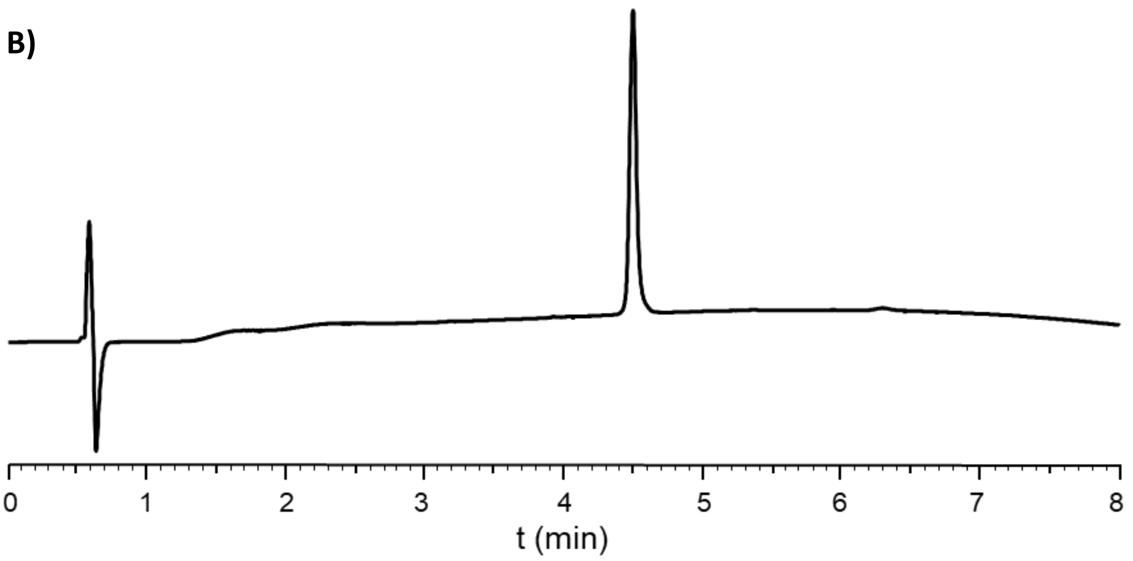

C)

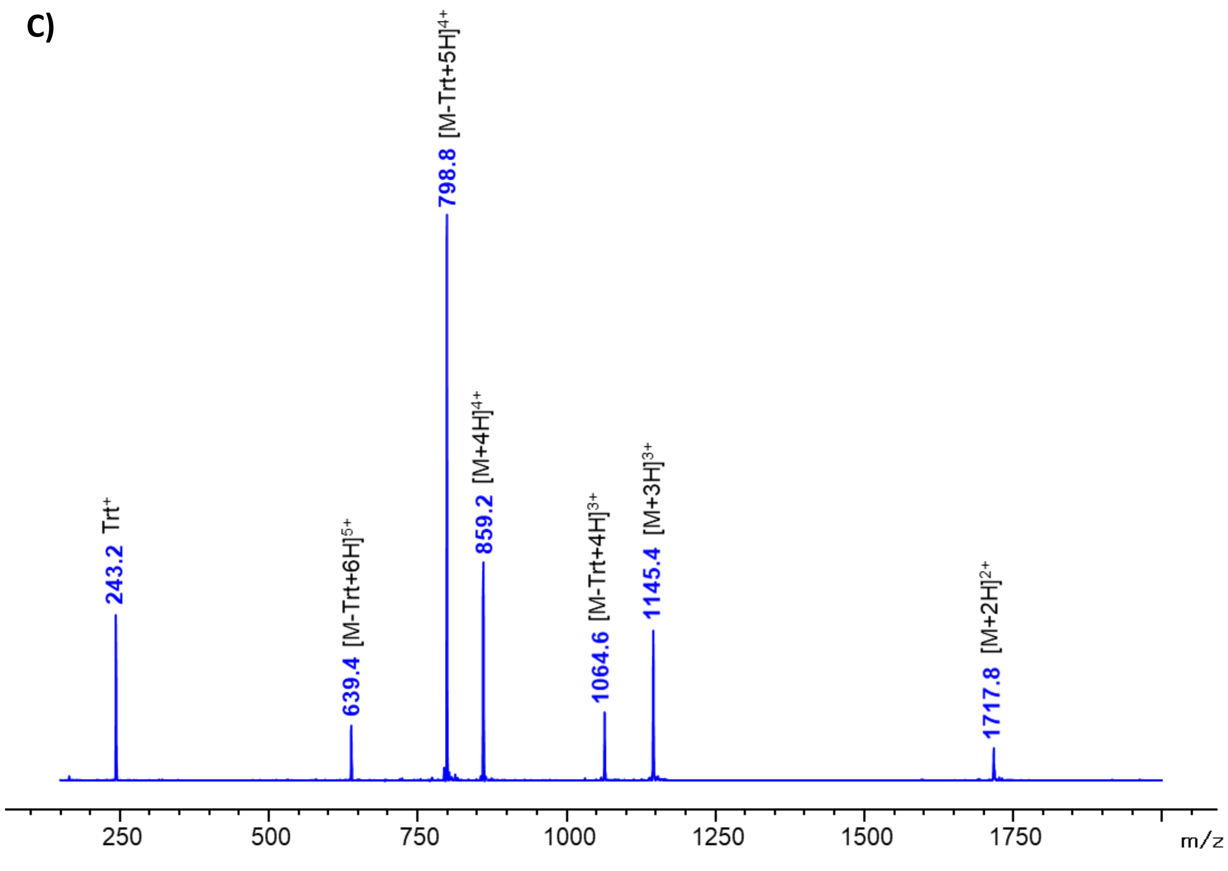

Figure S44. HPLC traces of (A) crude and (B) purified compound **6d-Trt** (λ = 214 nm). (C) ESI-MS spectrum of purified compound **6d-Trt**.

- **Trityl deprotection**

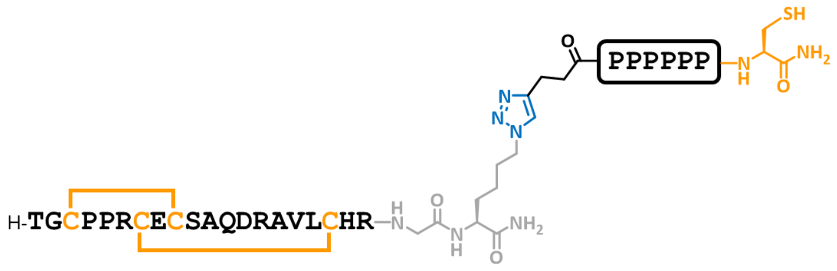

6d

Synthesis of **6d** was performed following protocol PS6.

ESI-MS: [M] average mass calculated for $C_{132}H_{208}N_{46}O_{37}S_5$: 3191.7, found: 3191.1 (deconvoluted).

HPLC purification: The crude peptide was used for disulfide pattern disruption assay without any further purification.

HPLC analysis: t_R = 3.55 min (Chromolith, gradient: 10-40% Solv. B over 7.5 min).

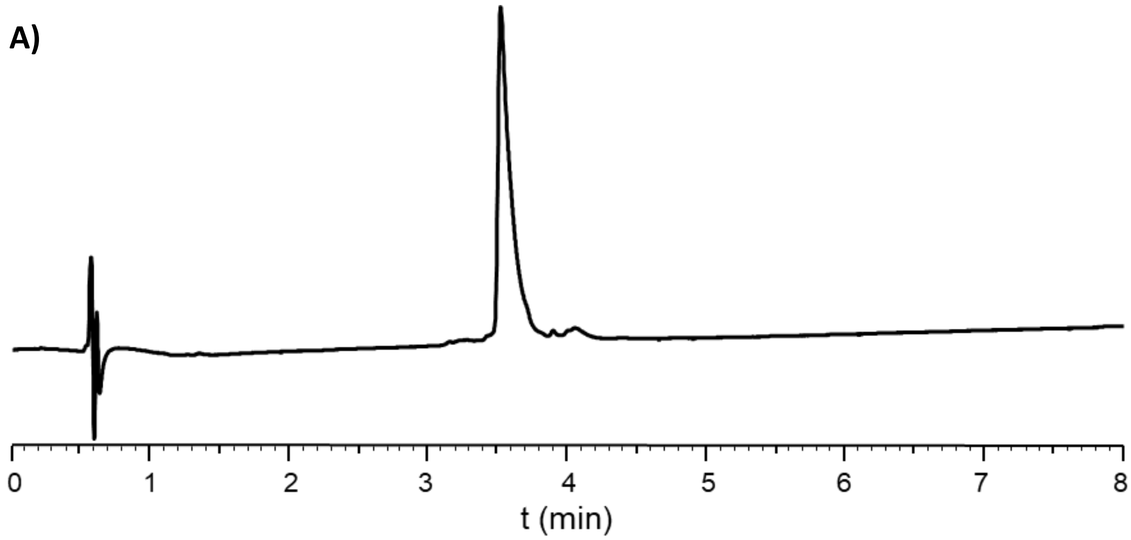

B)

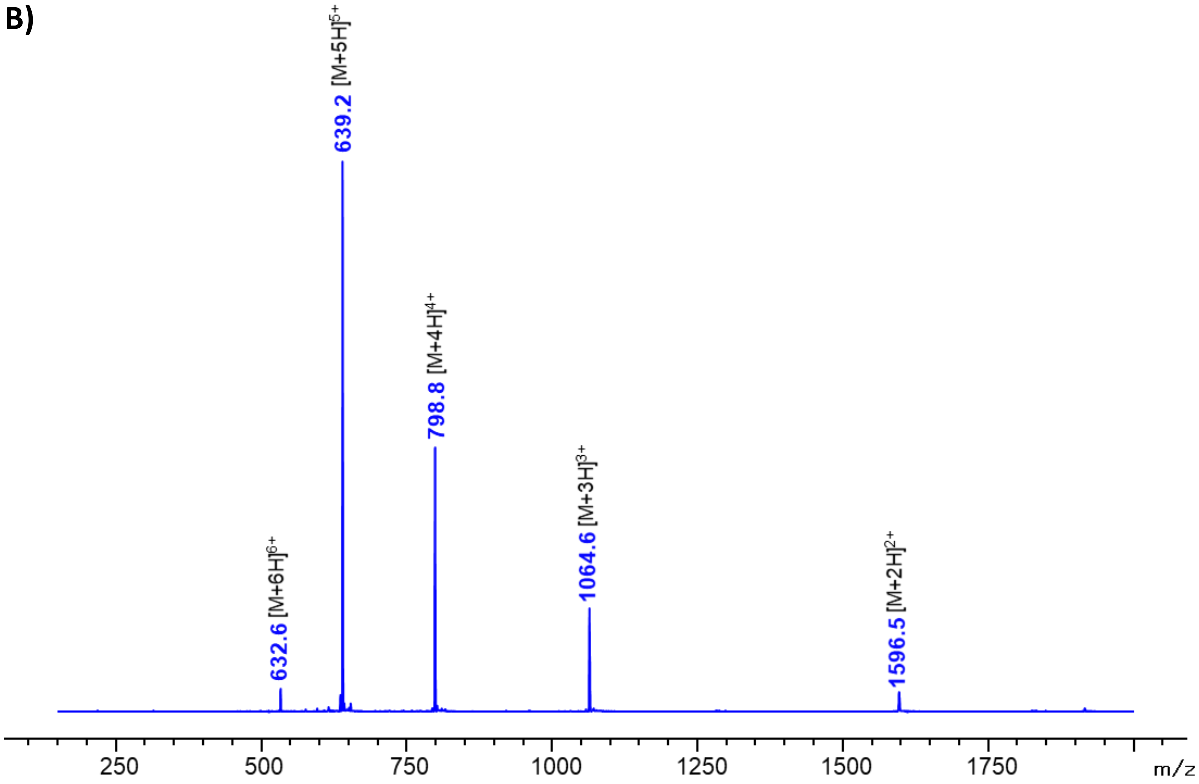

Figure S45. (A) HPLC trace of crude compound **6d** (λ = 214 nm). (B) ESI-MS spectrum of crude compound **6d.**

7-5 Synthesis of 6e

- **CuAAC**

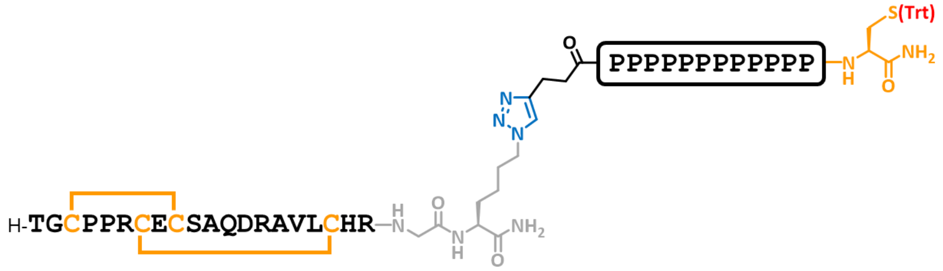

6e-Trt

Synthesis of **6e-Trt** was performed following protocol PS5.

ESI-MS: [M] average mass calculated for $C_{181}H_{264}N_{52}O_{43}S_5$: 4016.7, found: 4015.8 (deconvoluted).

HPLC purification: Nucleosil C18, gradient: 30-60% Solv. B over 20 min.; 53% yield.

HPLC analysis: t_R = 4.54 min. (Chromolith, gradient: 5-80% Solv. B over 7 min.).

A)

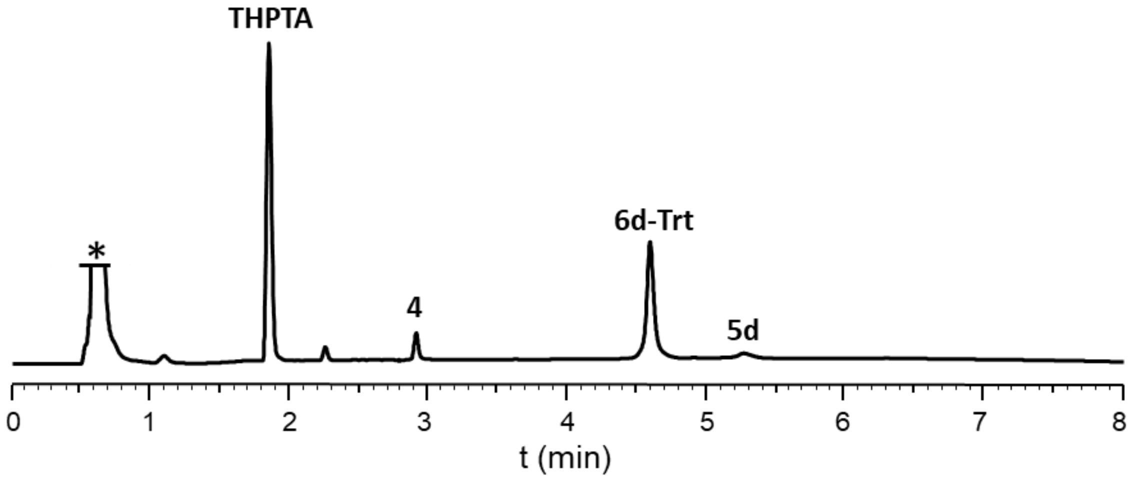

* DMSO

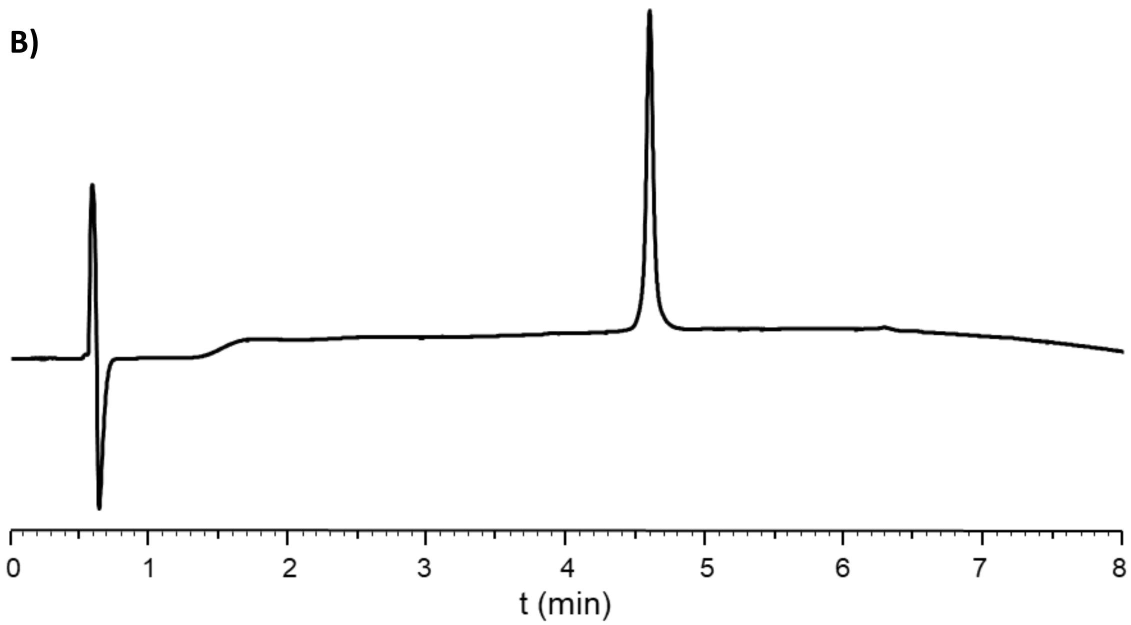

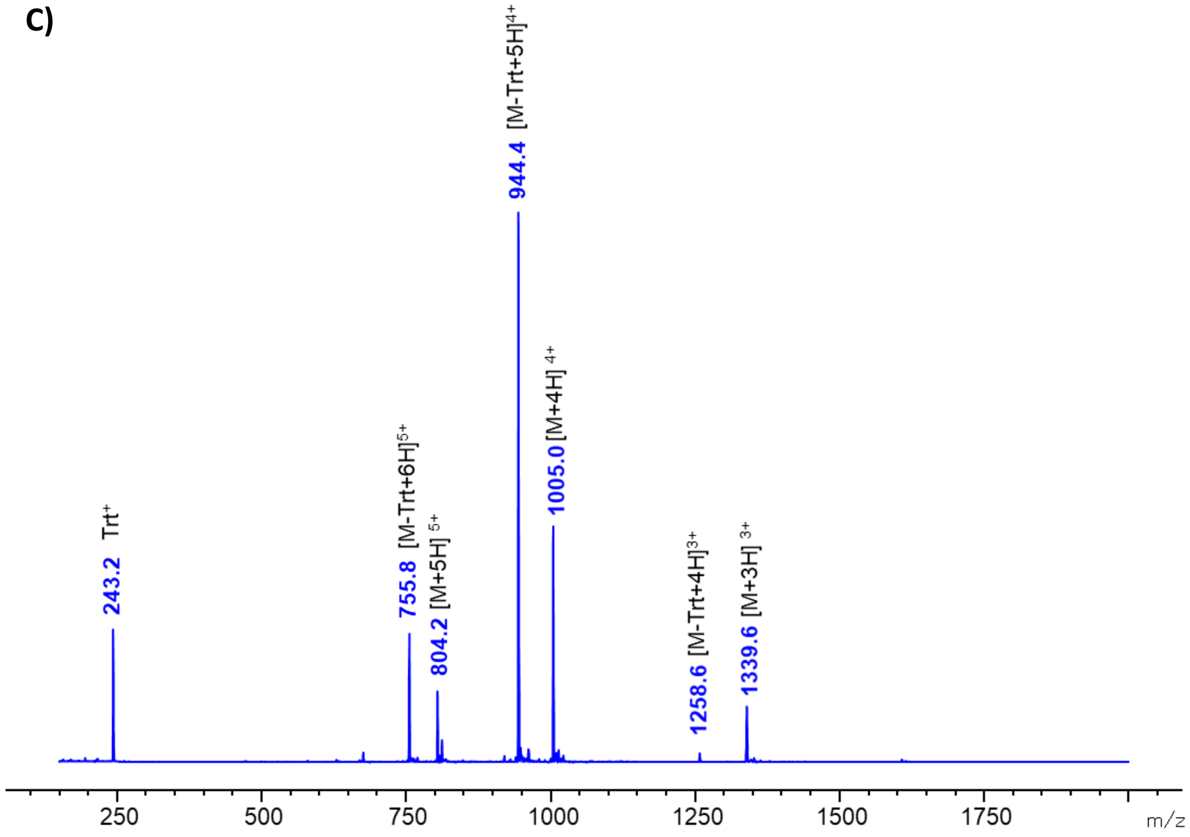

Figure S46. HPLC traces of (A) crude and (B) purified compound **6e-Trt** (λ = 214 nm). (C) ESI-MS spectrum of purified compound **6e-Trt**.

- **Trityl deprotection**

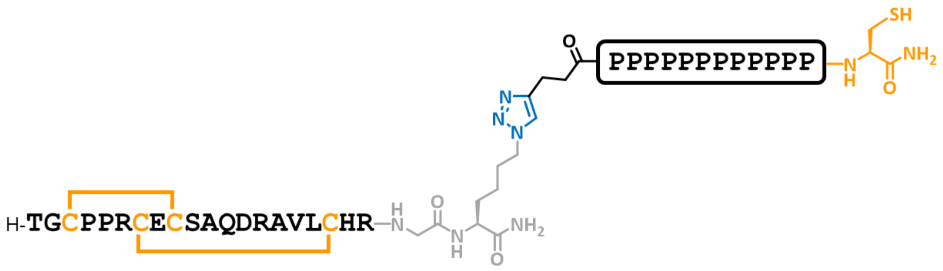

6e

Synthesis of **6e** was performed following protocol PS6.

ESI-MS: [M] average mass calculated for $C_{162}H_{250}N_{52}O_{43}S_5$: 3774.4, found: 3773.8 (deconvoluted).

HPLC purification: The crude peptide was used for disulfide pattern disruption assay and for conjugation to KLH without any further purification.

HPLC analysis: t_R = 3.79 min (Chromolith, gradient: 10-40% Solv. B over 7.5 min).

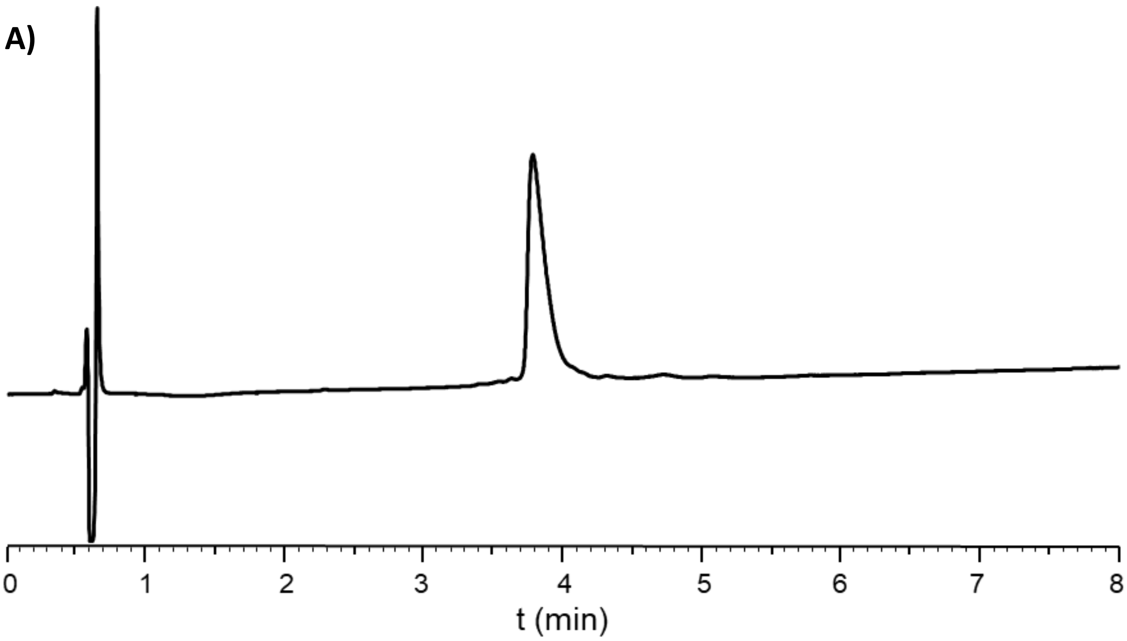

B)

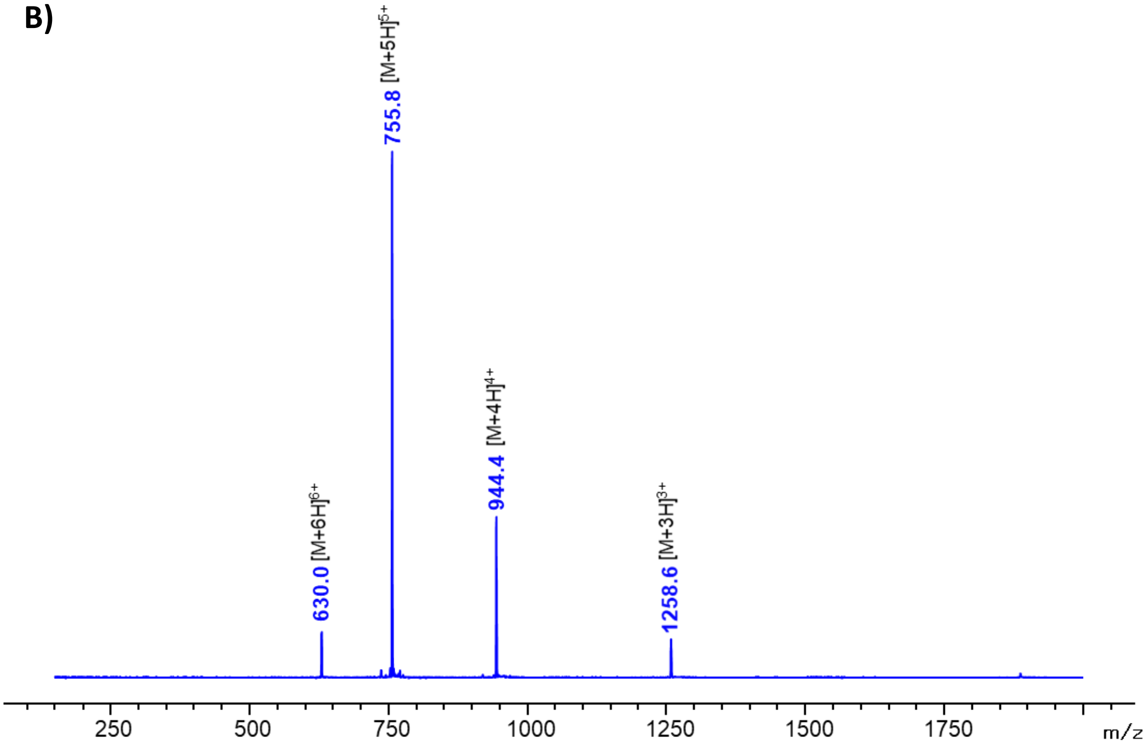

Figure S47. (A) HPLC trace of crude compound **6e** (λ = 214 nm). (B) ESI-MS spectrum of crude compound **6d.**

8- Structural characterization of the LINGO-1[1-20]-P_{12}C 6e construct by circular dichroism

Circular dichroism (CD) analyses[13] were performed on a Jasco J-810 spectropolarimeter equipped with a cell with an optical path length of 1 mm. All samples were dissolved in water at a 50 µM concentration. A baseline (cell filled with pure water) was recorded separately and subtracted. CD spectra were taken between 185 and 280 nm at 20 °C, using a 2 nm bandwidth, 2 s integration time, 1 nm data pitch and 50 nm/min scan speed for spectral acquisition. Spectra are reported as the averages of 30 scans, and were smoothed using the Savitzky-Golay algorythm (n = 11). The results are presented as molar ellipticity [θ] and molar circular dichroism ΔƐ, calculated using the following formulas:

$$[\theta] = \frac{\theta_{obs}}{10 \times l \times c} \quad \text{and} \quad \Delta\varepsilon = \frac{\theta_{obs}}{(N\text{-}1) \times 3280 \times l \times c}$$

with:

[θ] = molar ellipticity (deg.cm^2.$dmol^{-1}$)
ΔƐ = molar circular dichroism (M^{-1}.cm^{-1})
θ_{obs} = observed ellipticity (mdeg)
N = number of amino acids
l = pathlenght (cm)
c = concentration (M)

Note that molar circular dichroism (ΔƐ) of peptides and proteins corresponds to a mean value per peptide bond, which is also the case for mean residue ellipticity $[\theta]_{MRW}$, equal to the molar ellipticity divided by the number of peptide bonds (N-1). Comparing the CD spectrum of construct **6e** with the sum of spectra for **4** and **5e-SH** would not be apprioriate if expressed in such units rather than in molar ellipticity.

To prevent thiol oxidation, P_{12}C(SH) (**5e-SH**) and LINGO-1[1-20]-*Tz*-P_{12}C(SH) (**6e**) peptide aliquots were diluted under argon in previously deoxygenated water and transferred to CD cuvettes under an argon atmosphere. Pure **5e-SH** for spectrum acquisition was obtained from the *S*-protected peptide **5e**, taking advantage of having it in a purified form. An aliquot was treated following protocol PS2 for 1 h, not requiring any further purification of resulting **5e-SH** (quantitative yield).

A)

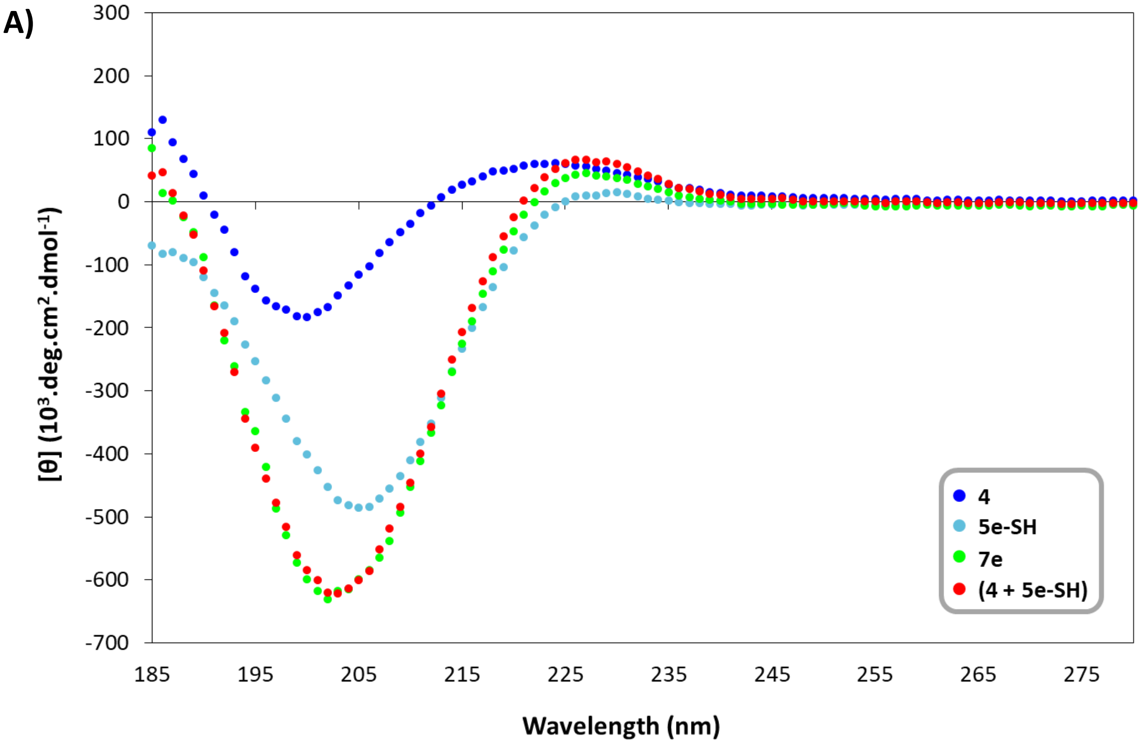

B)

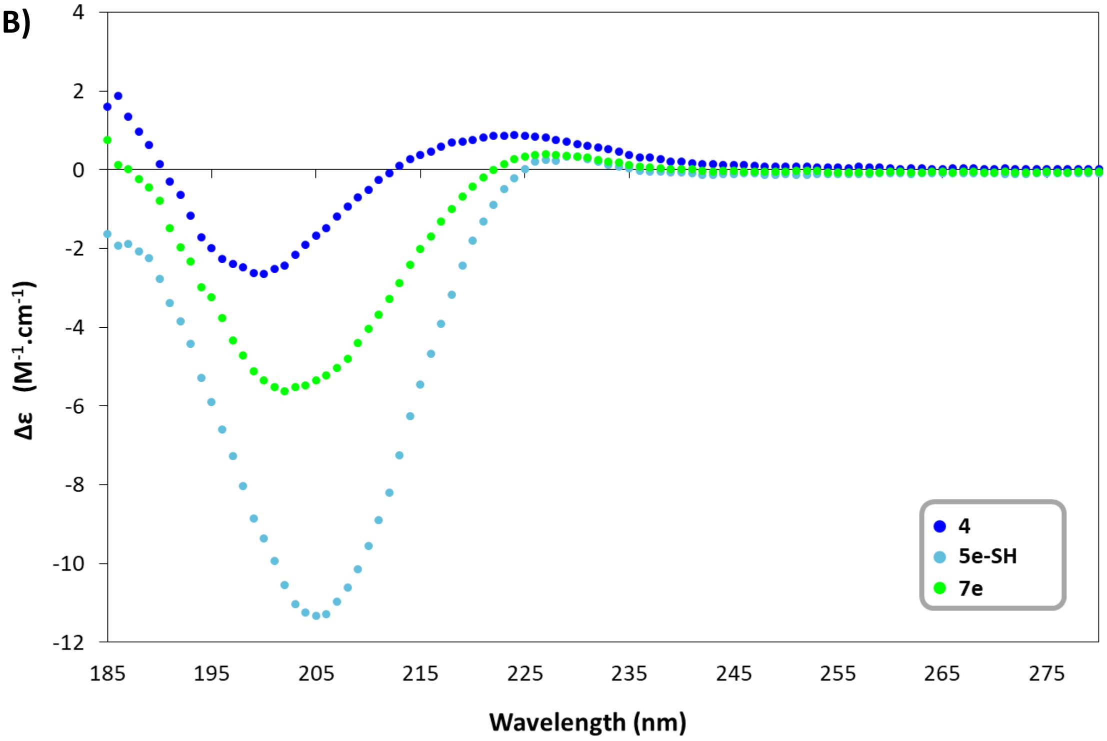

Figure S48. (A) CD spectra of **4**, **5e-SH** and **6e**, and the sum of **4** and **5e-SH** signals, reported as molar ellipticity; (B) CD spectra of **4**, **5e-SH** and **6e** reported as molar circular dichroism (ΔƐ).

Table S5. Measured ellipticities for compounds **4**, **5e-SH** and **6e**.

λ (nm)	Θ_{obs} (mdeg)			λ (nm)	Θ_{obs} (mdeg)		
	**4**	**5e-SH**	**6e**		**4**	**5e-SH**	**6e**
280	0.10	-0.17	-0.30	232	1.98	0.45	1.44
279	0.07	-0.19	-0.24	231	2.12	0.59	1.73
278	0.08	-0.17	-0.26	230	2.26	0.74	1.87
277	0.07	-0.19	-0.34	229	2.50	0.72	2.01
276	0.08	-0.20	-0.38	228	2.60	0.52	2.10
275	0.05	-0.17	-0.30	227	2.83	0.52	2.25
274	0.06	-0.20	-0.34	226	2.90	0.44	2.14
273	0.05	-0.21	-0.35	225	3.01	0.05	1.87
272	0.11	-0.23	-0.33	224	3.07	-0.44	1.49
271	0.13	-0.22	-0.29	223	3.01	-1.05	0.82
270	0.11	-0.19	-0.35	222	3.01	-1.90	-0.06
269	0.09	-0.16	-0.32	221	2.88	-2.81	-1.06
268	0.11	-0.16	-0.25	220	2.60	-3.85	-2.35
267	0.14	-0.18	-0.24	219	2.46	-5.22	-3.82
266	0.12	-0.19	-0.32	218	2.41	-6.79	-5.52
265	0.09	-0.17	-0.29	217	2.03	-8.36	-7.30
264	0.09	-0.17	-0.29	216	1.61	-10.01	-9.46
263	0.13	-0.20	-0.31	215	1.34	-11.68	-11.28
262	0.15	-0.22	-0.30	214	0.93	-13.42	-13.49
261	0.07	-0.19	-0.28	213	0.33	-15.55	-16.11
260	0.12	-0.18	-0.23	212	-0.30	-17.58	-18.35
259	0.21	-0.19	-0.32	211	-0.90	-19.08	-20.57
258	0.23	-0.22	-0.34	210	-1.78	-20.49	-22.64
257	0.26	-0.22	-0.34	209	-2.43	-21.76	-24.64
256	0.16	-0.23	-0.33	208	-3.20	-22.72	-26.89
255	0.23	-0.23	-0.36	207	-4.08	-23.51	-28.24
254	0.25	-0.22	-0.31	206	-5.14	-24.17	-29.22
253	0.23	-0.22	-0.18	205	-5.76	-24.26	-29.95
252	0.28	-0.26	-0.12	204	-6.60	-24.10	-30.74
251	0.29	-0.25	-0.17	203	-7.43	-23.66	-30.90
250	0.29	-0.23	-0.21	202	-8.38	-22.61	-31.52
249	0.30	-0.26	-0.25	201	-8.74	-21.30	-30.88
248	0.30	-0.27	-0.25	200	-9.16	-20.07	-29.95
247	0.35	-0.19	-0.24	199	-9.05	-18.97	-28.63
246	0.43	-0.16	-0.21	198	-8.57	-17.20	-26.48
245	0.44	-0.21	-0.18	197	-8.29	-15.57	-24.31
244	0.47	-0.24	-0.15	196	-7.83	-14.15	-21.04
243	0.49	-0.27	-0.19	195	-6.89	-12.63	-18.17
242	0.52	-0.29	-0.18	194	-5.92	-11.31	-16.67
241	0.57	-0.19	0.06	193	-4.01	-9.46	-13.04
240	0.70	-0.15	0.08	192	-2.19	-8.24	-11.00
239	0.75	-0.15	0.15	191	-1.03	-7.26	-8.25
238	0.94	-0.11	0.24	190	0.49	-5.96	-4.39
237	1.11	-0.08	0.38	189	2.18	-4.80	-2.44
236	1.12	-0.02	0.51	188	3.37	-4.46	-1.25
235	1.35	0.07	0.73	187	4.71	-4.02	0.09
234	1.60	0.18	1.01	186	6.49	-4.14	0.66
233	1.79	0.26	1.23	185	5.54	-3.47	4.27

9- Disulfide pattern disruption evaluation

20 nmol of each "DRP-spacer-thiol" construct (**6a**-**6e**) were incubated in 400 µL of thiol-maleimide conjugation buffer: 20 mM sodium phosphate pH 6.6, 150 mM NaCl, 35 mM EDTA, pH adjusted to 6.6. The buffer was thoroughly degassed through vacuum/argon cycles then added under argon to the peptide aliquot. Reactions were carried out either at room temperature (time points: 30 minutes and 2 hours) or 4 °C (time point: 24 hours) and monitored by HPLC and LC-MS (see article main text) after quenching by addition of two volumes of H_2O/MeCN/TFA 80:20:0.1.

Table S6. Proportion of intact construct after 24 hours incubation at 4 °C, determined by HPLC peak area at λ = 214 nm. Data used to generate Figure 2F in the article main text.

Compound		% of intact disulfide pattern after 30 minutes at RT	% of intact disulfide pattern after 120 minutes at RT
**6a**	$(GGGGS)_2GGC$	31	15
**6b**	$(GGGGS)_4GGGGC$	47	25
**6c**	$(GGGGS)_8GC$	64	42
**6d**	P_6C	72	42
**6e**	$P_{12}C$	97	95

10- Synthesis of KLH conjugate 7e and immunization

10-1 Conjugation of LINGO-1[1-20]-P12C(SH) to KLH

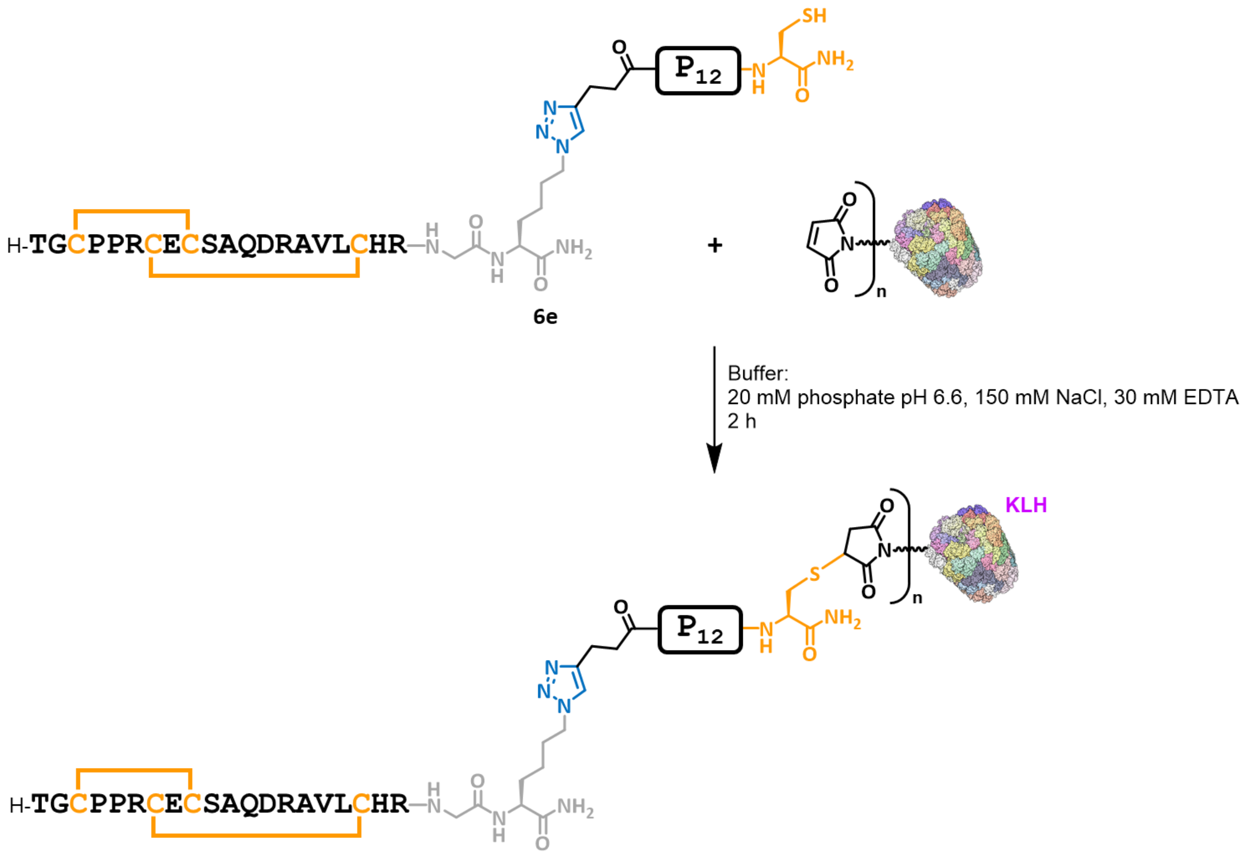

Scheme S5. Synthesis of immunogen **7e** through thiol/maleimide bioconjugation.

Maleimide-activated KLH (5 mg, Sigma-Aldrich, Cat. N° K0383) was reconstituted in 1 mL of water (resulting in a 10 mM sodium phosphate buffer, pH 6.6, with 0.115 mM NaCl, 1 mM EDTA, and 40 mM sucrose as stabilizer) and gently stirred for 1 hour. Construct **6e** (7.7 mg, 1.79 µmol) was solubilized in 1.5 mL of conjugation buffer (20 mM phosphate, pH 6.6, 150 mM NaCl, 30 mM EDTA) and then added to the KLH solution. After stirring for 2 hours at room temperature, 2.5 mL of phosphate-buffered saline (PBS, pH 7.4) was added to reach a final volume of 5 mL. The conjugate was purified by centrifugal filtration (Amicon Ultra-4, 30 kDa cut-off) using PBS as exchange buffer (4 cycles). A final volume of 1350 µL containing 470 µg of conjugate was recovered and used for follow-up immunization (quantity of KLH was determined by spectrophotometry at λ = 280 nm, following manufacturer recommendations).

10-2 Immunization

Immunization and polyclonal IgG purification was outsourced to Eurogentec (Liège, Belgium). One rabbit was injected with antigen **7e** (117 µg per dose), following a standard immunization protocol: four injections, at days 0, 14, 28 and 56. Blood was withdrawn before the first injection (pre-immune bleed), and after 38 (medium bleed I), 66 (medium bleed II) and 87 days (final bleed).

- **Polyclonal IgG purification**

90 mL of serum was loaded onto a Protein A affinity column. Bound antibodies were eluted using 100 mM glycine buffer at pH 2.5. The eluate was exchanged into PBS containing 0.01% thiomersal. A final volume of 41.8 mL was obtained, containing 416.6 mg of polyclonal antibodies (corresponding to a concentration of 9.97 mg/mL). The preparation was analyzed using a Bioanalyzer and showed 97.6% purity.

11- Evaluation of post-immunization serum and polyclonal IgG

11-1 Peptide-based ELISA assays

- **ELISA protocol**

100 µL of antigen solution (5 µg/mL stock solution in coating buffer: 100 mM bicarbonate/carbonate, pH 9.5) was added to each well of a MaxiSorp 96-well plate (Thermo Scientific) and incubated overnight at 4 °C. The wells were washed with PBS-T (PBS containing 0.05% *v*/*v* Tween® 20, 3 x 200 µL per well) and blocked with PBS-BSA (PBS containing 1% *w*/*v* Bovine Serum Albumin (BSA), 1.5 h at 25 °C, 200 µL/well). The wells were washed again with PBS-T (3 x 200 µL) and loaded with serum dilution in PBS-BSA (100 µL/well, 1.5 h at 25 °C). After washing with PBS-T (3 x 200 µL), the wells were incubated with 100 µL of horseradish peroxidase (HRP)-conjugated anti-rabbit goat immunoglobulins (H+L) secondary antibody (Invitrogen, 1 mg/mL, dilution to 1/5000 in PBS-BSA, 1 h at 25 °C). After a final wash with PBS-T (3 x 200 µL), 3,3',5,5'-tetramethylbenzidine (TMB, Diagomics - Cat. N° C08-1L) substrate solution was added (100 µL/well) and incubated at room temperature, hidden from light. The reaction was quenched after 15 minutes by the addition of a 0.2 M H_2SO_4 solution (100 µL) and the absorbance was immediately read at 450 nm using a microplate reader (CLARIOstar, BMG Labtech).

Four different antigens were used for coating:

- **6e-Aca**, a derivative of the LINGO-1[1-20]-P12C construct **6e**, in which the cysteine residue of the linker has been alkylated with iodoacetamide, in order to prevent any possible disulfide exchange during the assay.
- **4**, the azido-functionalized LINGO-1[1-20] peptide, not coupled to the oligoproline linker.
- **5e-SH**, the alkyne-functionalized oligoproline linker, not coupled to the LINGO-1 peptide.
- "**4D**", an analogue of the azido-functionalized LINGO-1[1-20] peptide **4**, in which the LINGO-1[1-20] segment was synthesized from D-amino acids (and achiral Gly), having identical physicochemical properties as **4**, but mirror-image three-dimensional structure, and used as a negative control.

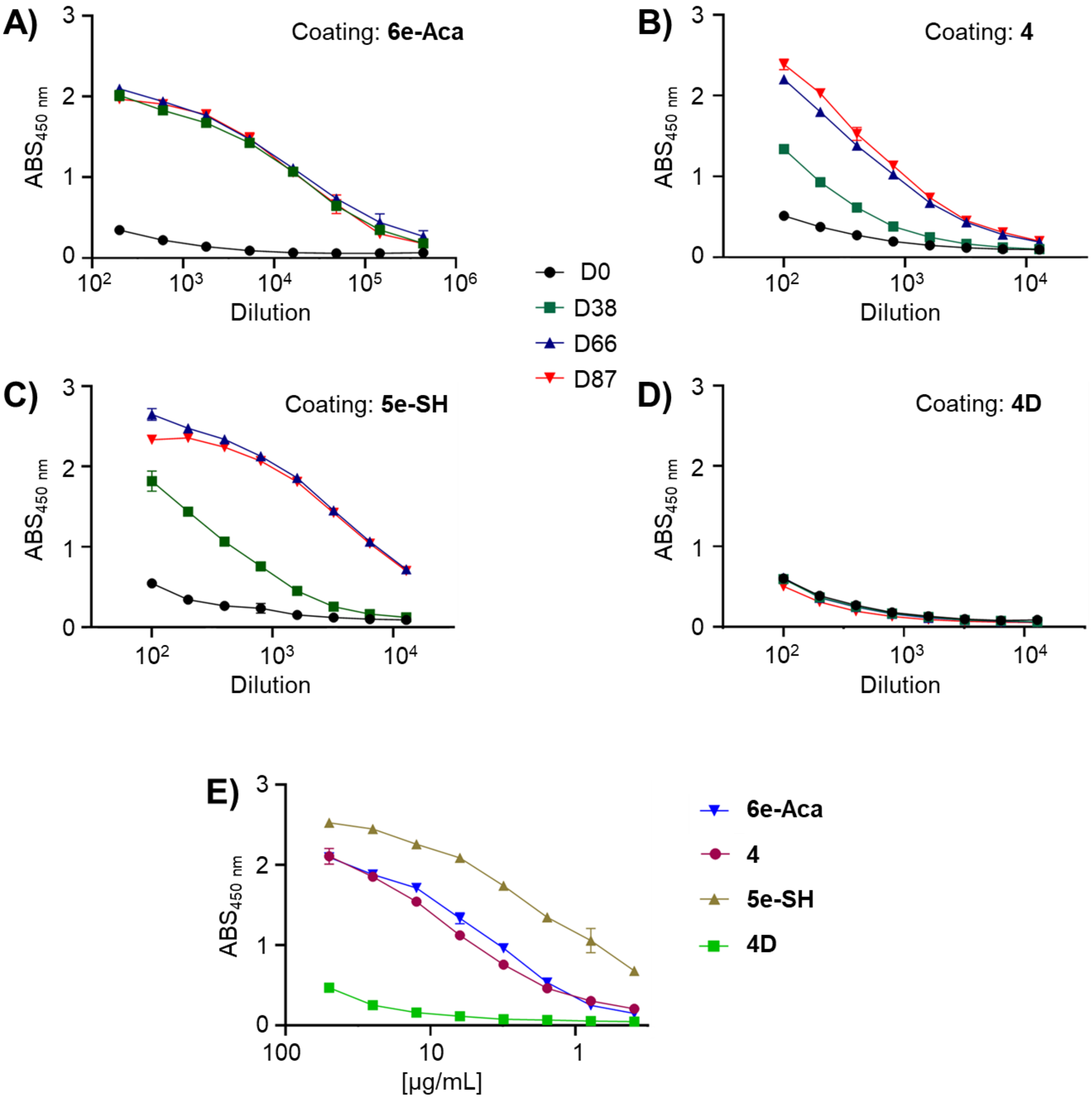

Figure S49. ELISA titration curves as a function of serum dilution using pre-immune serum (D0), and day 38 (D38), day 66 (D66), day 87 (D87) post-immunization sera using as coated antigens **6e-Aca** (A), **4** (B), **5e-SH** (C) and **4D** (D). ELISA titration curves as a function of polyclonal IgG concentration (E).

Table S6. Compounds used for coating ELISA wells.

Code	Structure
**6e-Aca**	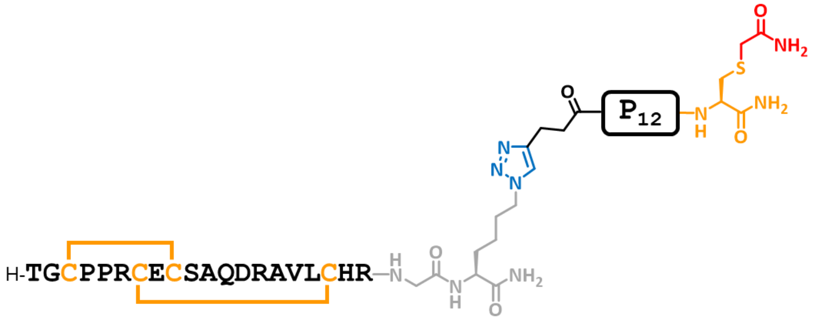
**4**	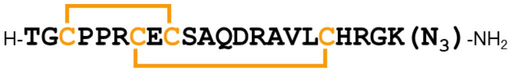
**5e-SH**	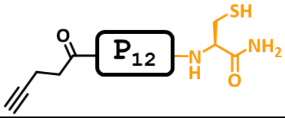
**4D**	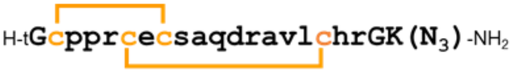

- **Synthesis of 6e-Aca**

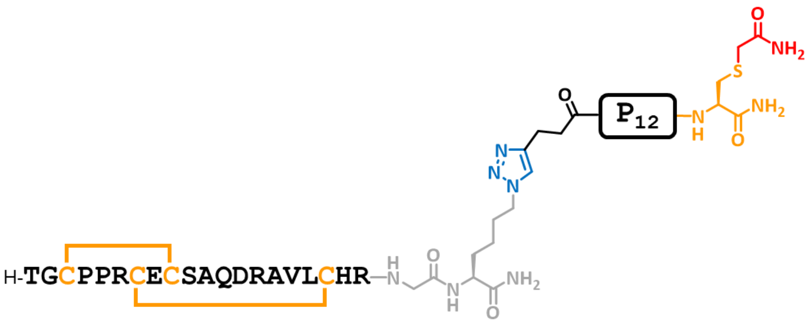

6e-Aca

Under an argon atmosphere, compound **6e** (0.115 µmol) was solubilized in 92 µL of deoxygenated water. To this solution, iodoacetamide (32 µg, 0.173 mmol, 1.5 equiv., solution in 12 µL deoxygenated water) was added under argon. Subsequently, 12 µL of 1 M Tris buffer pH 8.6, was added (1 mM final peptide concentration). The reaction mixture was stirred at room temperature for 2 hours, protected from light. The reaction was quenched by adding 120 µL of 1% TFA in water, and the crude product was immediately purified by RP-HPLC.

ESI-MS: [M] average mass calculated for $C_{164}H_{253}N_{53}O_{44}S_5$: 3831.5, found: 3830.6 (deconvoluted).

HPLC purification: Nucleosil C18, gradient: 30-60% Solv. B over 20 min.; 42% yield.

HPLC analysis: t_R = 4.49 min. (Chromolith, gradient: 3-50% Solv. B over 7 min.).

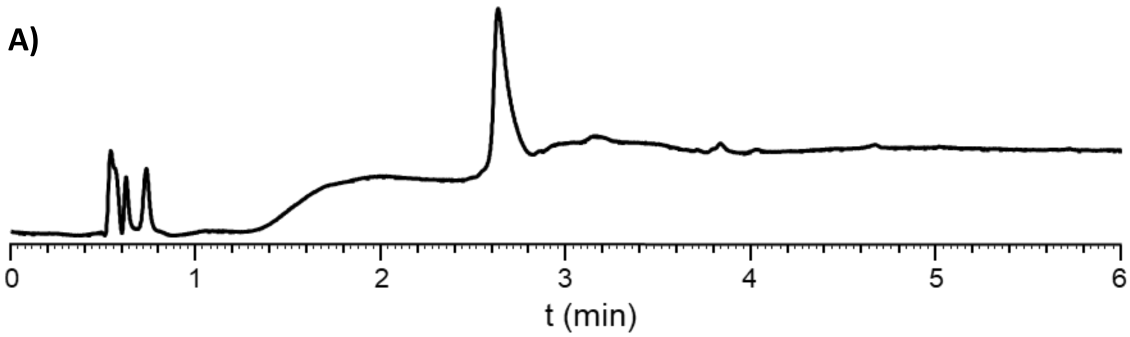

B)

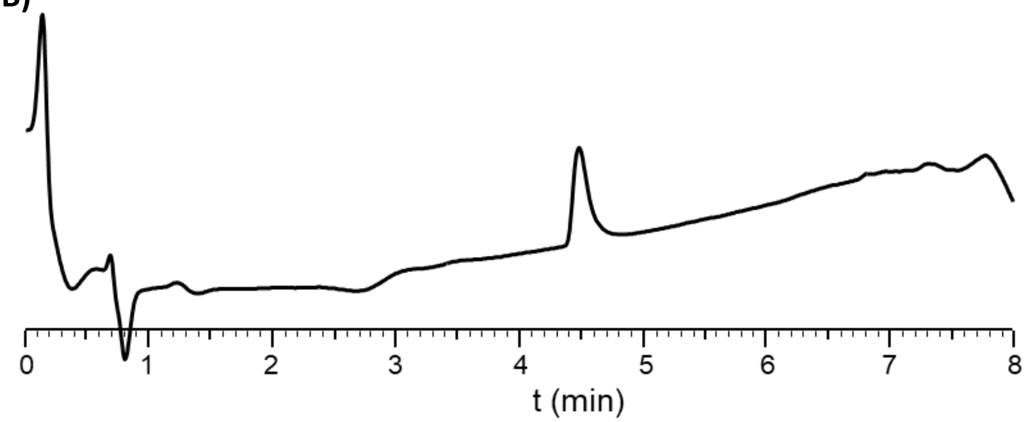

C)

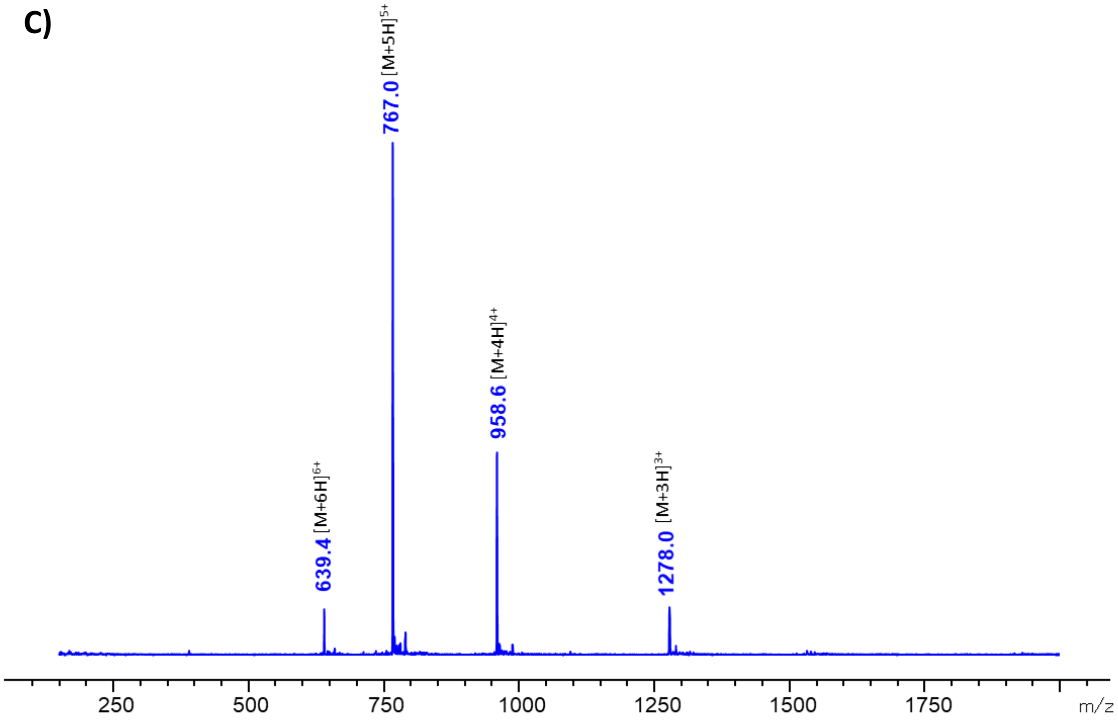

Figure S50. HPLC traces of (A) crude (Chromolith, gradient: 5-50% Solv. B over 5 min.) and (B) purified (Chromolith, gradient: 3-50% Solv. B' over 5 min.) compound **6e-Aca** (λ = 214 nm). (C) ESI-MS spectrum of purified compound **6e-Aca**.

- **Synthesis of 4D**

Sequence: H-tGcpprcecsaqdravlchrGk(N_3)-NH_2 (**4D**)

4D was synthesized using the same procedure as per compound **4**.

ESI-MS: [M] average mass calculated for $C_{94}H_{154}N_{38}O_{29}S_4$: 2408.7, found: 2408.2 (deconvoluted).

HPLC purification: [4 mg/mL] Nucleosil C18, gradient: 10-30% Solv. B over 40 min.; 8% overall yield.

HPLC analysis: t_R = 1.78 min. (Chromolith, gradient: 15-35% Solv. B over 5 min.).

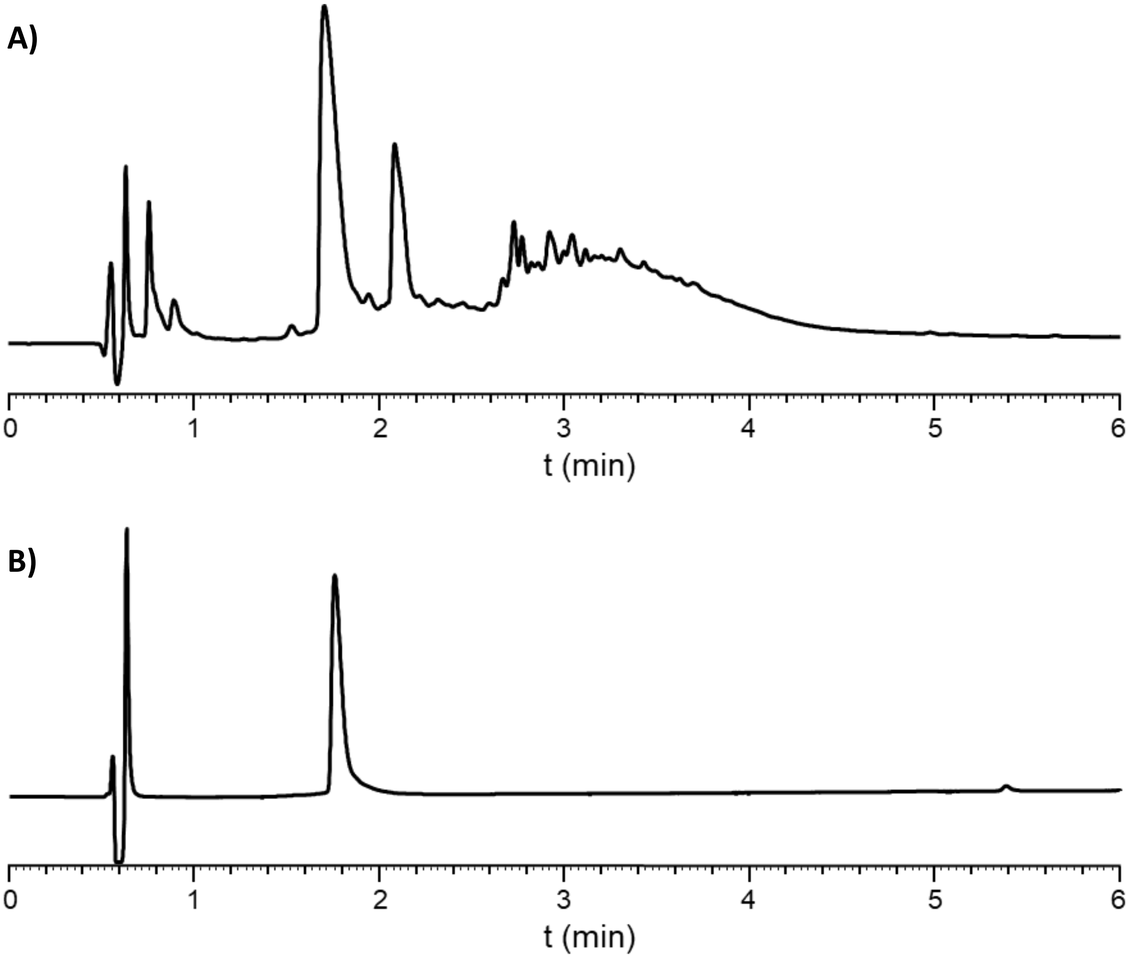

C)

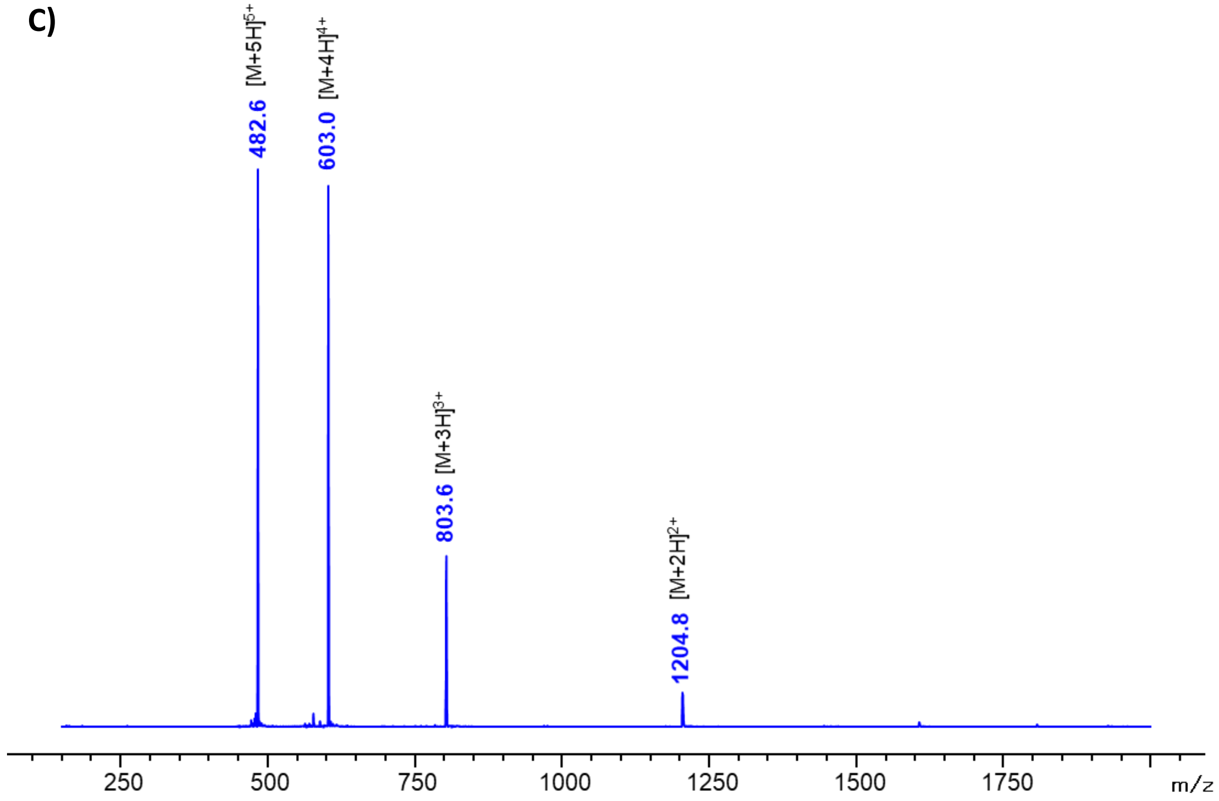

Figure S51. HPLC traces of (A) crude and (B) purified compound **4D** (λ = 214 nm). (C) ESI-MS spectrum of purified compound **4D**.

11-2 Cell-based ELISA assays

ELISA was performed using HEK293 cells transfected with a plasmid encoding the LINGO-1 protein using Lipofectamine™ 2000 (Thermo Fisher Scientific), according to the manufacturer's instructions. Approximately 40,000 cells (150 µL per well) were seeded into a 96-well plate (Greiner #655098) that had been pre-coated with collagen (Gibco™ A1048301). Day after, cells were washed carefully with PBS (100 µL) and were maintained in fresh DMEM medium with addition of 10% FBS (150 µL) for 24 h at 37 °C with 5% CO_2 atmosphere until experiments. Forty-eight hours after transfection, cells were blocked with PBS containing 1% BSA (200 µL) for 1 hour on ice. Following blocking, various concentrations of anti-LINGO-1 polyclonal antibody diluted in PBS–1% BSA were added (100 µL) and incubated for 2 hours on ice. Detection was performed using an anti-rabbit antibody (ThermoFisher #31460) conjugated to HRP (1:5000 dilution in PBS–1% BSA, 100 µL), incubated for 1 hour on ice. After incubation with the anti-LINGO-1 polyclonal antibody and subsequently with the secondary antibody, cells were washed with PBS containing 0.5% BSA (200 µL). Tween-20 was not used in the washing buffer to avoid potential cytotoxic effects on the live cells, which could compromise the integrity of the assay before its completion. Following the final wash, TMB substrate solution (100 µL) was added, and the plate was incubated at room temperature with gentle agitation, protected from light, until a blue color developed. Finally, reaction was stopped with 1 M sulfuric acid (100 µL), and the measurements of chromogenic reaction were read at 450 nm.

Table S7. Raw ELISA data underlying the graph presented in Figure 5A (N = 4 for each experiment).

Polyclonal IgG concentration (µg/ml)	LINGO-1 - expressing HEK-297 (Abs 450 nm)	Non-transfected HEK-297 (negative ctrl) (Abs 450 nm)
0	0	0
0.0005	0.077 ± 0.021	0 ± 0.077
0.0025	0.102 ± 0.031	0.011 ± 0.034
0.005	0.212 ± 0.027	0.067 ± 0.014
0.025	0.236 ± 0.044	0 ± 0.085
0.05	0.303 ± 0.026	0.062 ± 0.064
0.5	0.319 ± 0.025	0.081 ± 0.006

11-3 Western blot assays

HEK293 cells were transfected with plasmids encoding the HA-LINGO-1, HA-LINGO-2, HA-LINGO-3 and HA-LINGO-4 protein using Lipofectamine™ 2000 (Thermo Fisher Scientific), according to the manufacturer's instructions. Approximately 500,000 cells (2 mL per well) were seeded into a 6-well plate (Sigma Aldrich #Z707759-126EA) that had been pre-coated with collagen (Gibco™ A1048301). Day after, cells were washed carefully with PBS and were maintained in fresh DMEM medium with addition of 10% FBS for 24 h at 37 °C with 5% CO_2 atmosphere until experiments. Forty-eight hours after transfection, cells were washed twice with cold PBS and lysed on ice for 30 min in lysis buffer containing 50 mM Tris pH 7.5, 150 mM NaCl, 10 mM EDTA, 1 % Triton X-100 and a protease inhibitor cocktail (Santa Cruz Biotechnology, Cat. N° sc-24948A). Cell lysates were centrifuged at 10,000 x g for 10 min. Following cell lysis in RIPA buffer and centrifugation to recover the supernatant, protein concentrations were determined using the Bio-Rad DC Protein Assay (Bio-Rad Laboratories), according to the manufacturer's instructions. Supernatant was then solubilized in Laemmli buffer with 0.1% β-mercaptoethanol. Samples were resolved by electrophoresis on 10% SDS-PAGE and transferred electrophoretically to nitrocellulose membranes (GE Healthcare Life Sciences). Nitrocellulose membranes were washed in Tris-buffered saline (TBS; pH 7.4) containing 0.1% Tween-20 (TBS-T; 0.1%) and blocked with 5% (*w*/*v*) dry milk TBS-T 0.1% for 1h at RT. Blots were probed with commercial antibody anti-LING01 (R&D Systems, Cat. N° AF3086) (1:1000) or polyclonal anti-LINGO-1 obtained in this work (1:5000), anti-HA (Roche, Cat. N° 11867423001) (1:1000) and anti-GAPDH antibodies (Cell Signaling, Cat. N° #2118) (1:2000). Horseradish peroxidase–conjugated secondary antibodies (anti-rabbit, anti-goat, or anti-rat; 1:30,000) were used. Immunoreactive bands were detected using the Dura detection kit. Protein levels were quantified from immunoblots using Quantity One software (Bio-Rad).

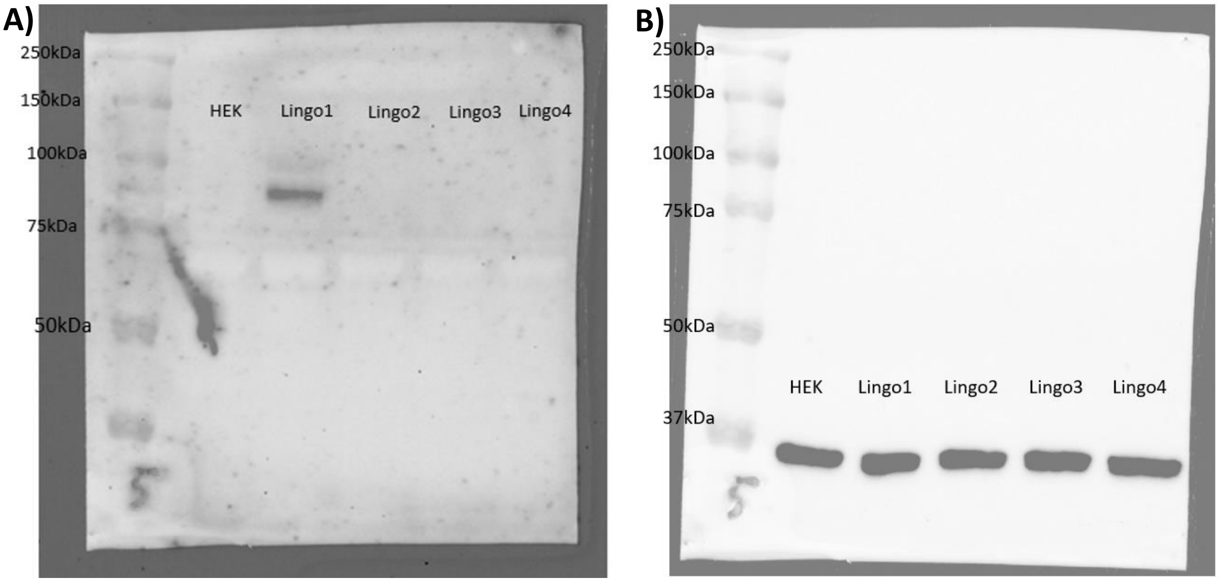

Anti-Lingo1 POLYCLONAL
(1/5000 TBST/milk)

Anti-GAPDH
(1/2000 TBST/milk)

Figure S52. Western blot analysis of HEK-293 cells transfected with LINGO-1–4 constructs. A distinct band was detected only for LINGO-1, demonstrating specific recognition of the generated polyclonal antibody. Equal protein amounts were loaded across all lanes based on pre-loading quantification, and GAPDH was used as a loading control to verify protein expression and transfer.

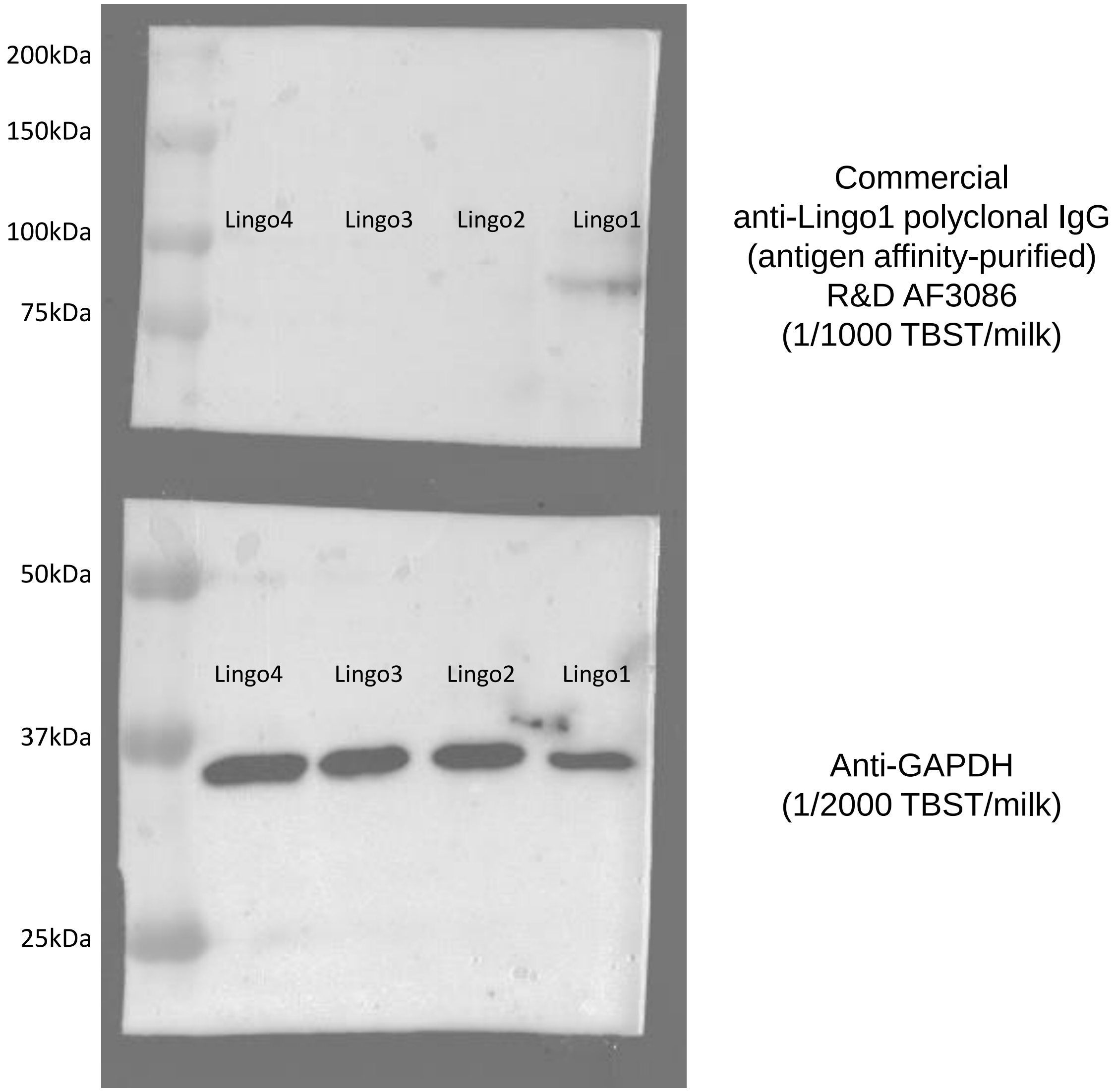

Figure S53. Western blot analysis of HEK-293 cells transfected with HA-LINGO-1–4 constructs, using a commercially available anti-LINGO-1 antibody (R&D Systems, Cat. N° AF3086) to confirm antibody specificity. Equal protein amounts were loaded across all lanes based on pre-loading quantification, and GAPDH was used as a loading control to verify protein expression and transfer. Notably, the commercial antibody was used at 1:1,000 dilution, whereas the generated polyclonal antibody achieved comparable efficacy at 1:5,000 dilution.

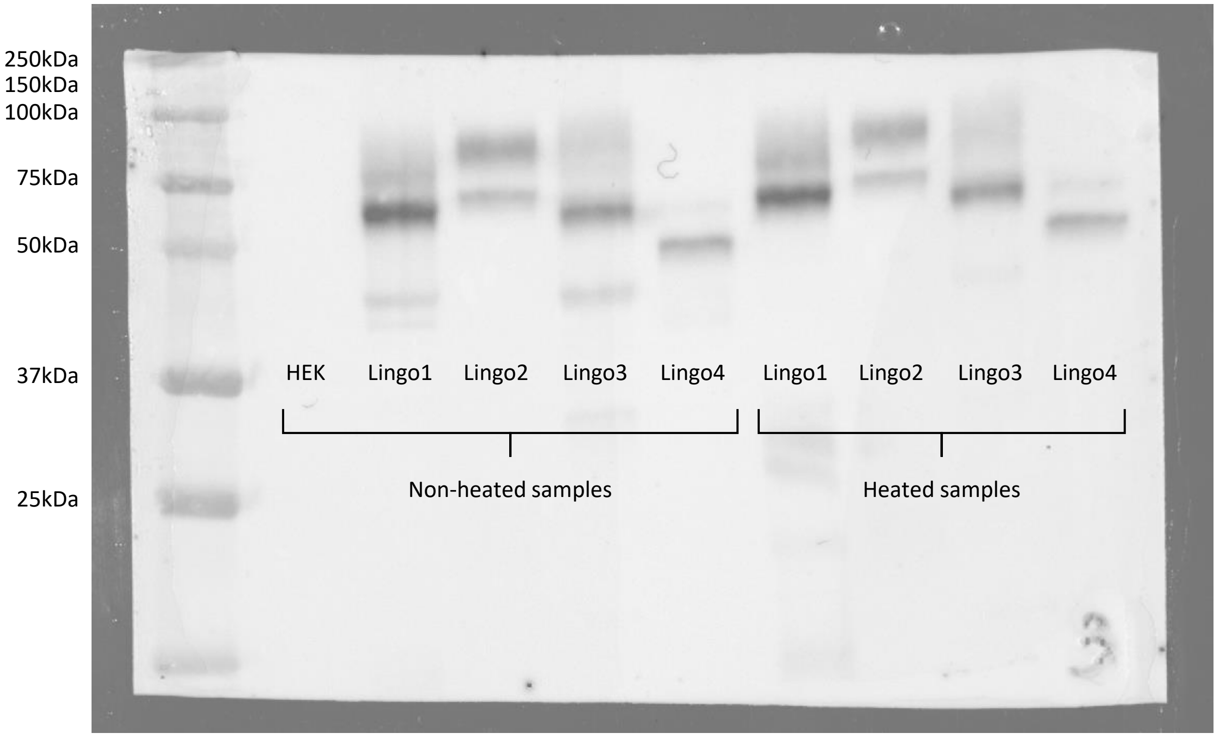

Figure S54. Western blot detection of HEK-293 cells transfected with HA-LINGO-1–4 constructs, using an anti-HA antibody to confirm expression of all LINGO proteins. To assess detectability, samples were analyzed under both heated and non-heated conditions.

12- References

[1] Schanda, P.; Kupce, E.; Brutscher, B., SOFAST-HMQC experiments for recording two dimensional heteronuclear correlation spectra of proteins within a few seconds. *J. Biomol. NMR.* **2005**, *33*, 199−211.

[2] Vranken, W. F.; Boucher W; Stevens TJ; Fogh, R. H.; Pajon, A.; Llinas, M.; Ulrich, E. L.; Markley, J. L.; Ionides, J.; Laue E. D., The CCPN data model for NMR spectroscopy: development of a software pipeline. *Proteins* **2005**, *59*, 687−696.

[3] Brunger, A. T.; Adams, P. D.; Clore, G. M.; DeLano, W. L.; Gros, P.; Grosse-Kunstleve, R. W.; Jiang, J. S.; Kuszewski, J.; Nilges, M.; Pannu, N. S.; Read, R. J.; Rice, L. M.; Simonson, T.; and Warren, G. L., Crystallography & NMR system: A new software suite for macromolecular structure determination. *Acta Crystallogr. D* **1998**, *54*, 905−921.

[4] Brunger, A. T., Version 1.2 of the Crystallography and NMR system. *Nat. Protoc.* **2007**, *2*, 2728−2733.

[5] Rieping, W.; Habeck, M.; Bardiaux, B.; Bernard, A.; Malliavin, T. E.; Nilges, M., ARIA2: Automated NOE assignment and data integration in NMR structure calculation. *Bioinformatics* **2007**, *23*, 381−382.

[6] Laskowski, R. A.; Rullmannn, J. A.; MacArthur, M. W.; Kaptein, R.; Thornton, J. M., AQUA and PROCHECK-NMR: Programs for checking the quality of protein structures solved by NMR. *J. Biomol. NMR* **1996**, *8*, 477−486.

[7] DeLano, W. L. (**2002**) The PyMOL Molecular Graphics System, DeLano Scientific, South San Francisco, CA.

[8] Song, Y.; DiMaio, F.; Yu-Ruei Wang, R.; Kim, D.; Miles, C.; Brunette, T.; Thompson, J.; Baker, D., High resolution comparative modeling with RosettaCM. *Structure* **2013**, *21*, 1735−1742.

[9] Laskowski, R. A., PDBsum 1 : A standalone program for generating PDBsum analyses. *Protein Sci.* **2022**, *31*, e4473.

[10) Goddard, T. D.; Huang, C. C.; Meng, E. C.; Pettersen, E. F.; Couch, G. S.; Morris, J. H.; Ferrin, T. E., UCSF ChimeraX: Meeting modern challenges in visualization and analysis. *Protein Sci.* **2018**, *27*, 14−25.

[11] Zhang, S.; Lin, F.; Hossain, M. A.; Shabanpoor, F.; Tregear, G. W.; Wade, J. D., Simultaneous Post-cysteine (S-Acm) Group Removal Quenching of Iodine and Isolation of Peptide by One Step Ether Precipitation. *Int. J. Pept. Res. Ther.* **2008**, *14*, 301−305.

[12] Landon, C.; Zhu, Y.; Mustafi, M.; Madinier, J.-B.; Lelièvre, D.; Aucagne, V.; Delmas, A. F.; Weisshaar, J. C., Real-Time Fluorescence Microscopy on Living E. coli Sheds New Light on the Antibacterial Effects of the King Penguin β-Defensin AvBD103b. *Int. J. Mol. Sci.* **2022**, *23*, 2057.

[13] Greenfield, N. J.; Fasman, G. D., Computed circular dichroism spectra for the evaluation of protein conformation. *Biochemistry* **1969**, *8*, 4108−4116.
